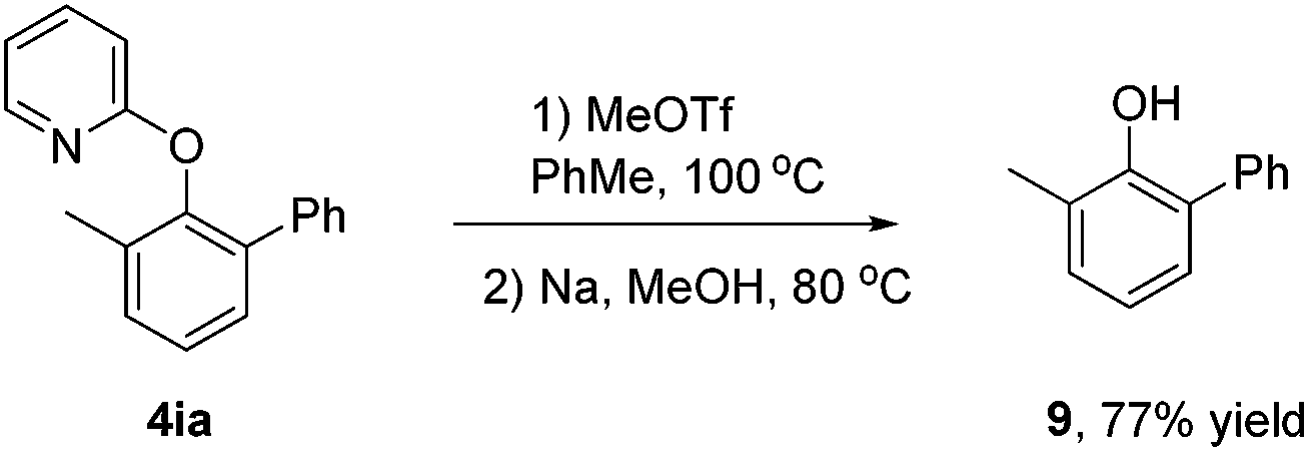# Stoichiometric to catalytic reactivity of the aryl cycloaurated species with arylboronic acids: insight into the mechanism of gold-catalyzed oxidative C(sp^2^)–H arylation†
†Electronic supplementary information (ESI) available: Detailed experimental procedures, analytical data. CCDC 1000469, 1007212 and 1019879. For ESI and crystallographic data in CIF or other electronic format see DOI: 10.1039/c4sc02070g


**DOI:** 10.1039/c4sc02070g

**Published:** 2014-10-09

**Authors:** Qian Wu, Chenglong Du, Yumin Huang, Xingyan Liu, Zhen Long, Feijie Song, Jingsong You

**Affiliations:** a Key Laboratory of Green Chemistry and Technology of Ministry of Education , College of Chemistry and State Key Laboratory of Biotherapy , West China Medical School , Sichuan University , 29 Wangjiang Road , Chengdu 610064 , PR China . Email: jsyou@scu.edu.cn ; Email: jingbolan@scu.edu.cn ; Fax: +86-28-85412203

## Abstract

Based on the well-defined five-membered aryl gold(iii) complexes, [Au(tpy)X_2_] (**3a** and **3b**) and [AuBr(Ph)(tpy)] (**7**), as well as the aryl gold(iii) complex [AuCl_2_(Ph)(tpy)] (**8**) (tpy = 2-(*o*-tolyl)pyridine) as reliable models, we present a detailed study of the mechanism for gold(iii)-catalyzed oxidative cross-coupling reactions between cycloaurable arenes and arylboronic acids. Here we report the direct evidence for a mechanistic proposal including arene C–H activation, transmetallation and biaryl reductive elimination. The chelation-assisted C–H activation strategy has been used for the development of the gold(iii)-catalyzed C–H bond arylation of arenes with aryl reagents to forge extended π-conjugated systems.

## Abstract 2

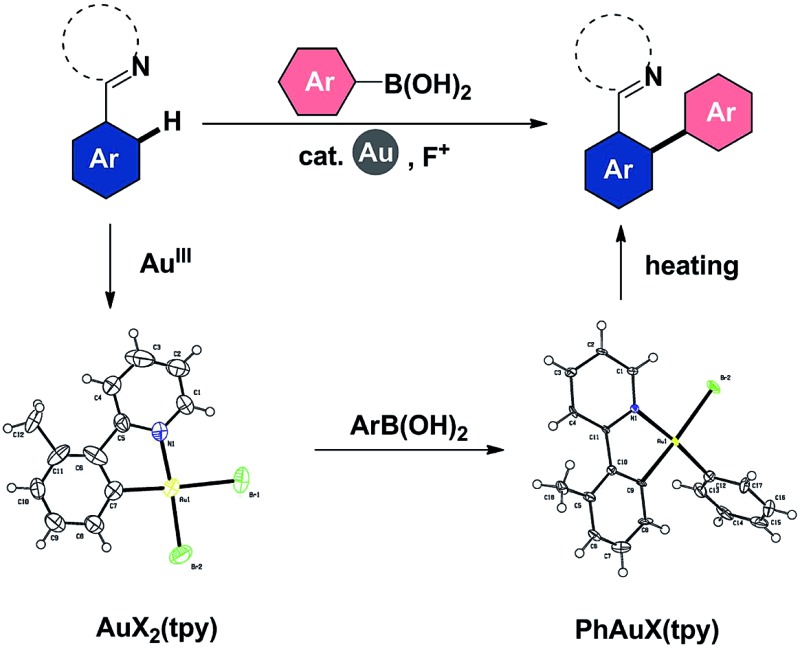
The aryl cycloaurated species are established as reliable models for understanding the gold(iii)-catalyzed oxidative cross-couplings between arenes and arylboronic acids.

## Introduction

Gold-catalyzed organic transformations have been placed under the spotlight during the past decade. Gold salts and complexes are most commonly used as a redox-neutral, carbophilic π acid for the activation of carbon–carbon multiple bonds such as alkynes, alkenes, and allenes towards nucleophilic attack.^1^ In contrast, the gold-catalyzed oxidative coupling reactions proposed to involve Au(i)/Au(iii) cycles are still underrepresented.^2^ In particular, gold-catalyzed C–H bond arylation of simple arenes to forge biaryl scaffolds is still in its infancy. In addition to a few examples of catalytic homo-coupling of simple arenes,^3^ stoichiometric homo- and hetero-coupling processes have nearly exclusively dominated the previous literature.^4–6^ It has proven to be a challenge to evolve a stoichiometric hetero-coupling between an unactivated arene and an aromatic nucleophile to a catalytic version. The only known catalytic scenario is the oxidative cross-coupling reaction between arylsilanes and simple arenes recently demonstrated by Lloyd-Jones, Russell and Ball.^7,8^ The development of such transformations may encounter two major obstacles: (1) the relatively high redox potential of the Au(i)/Au(iii) couple (*E* ^0^ = +1.41 V) traditionally precludes the catalytic cycles;^9^ and (2) there are considerable uncertainties in the reaction mechanism, due in part to the difficulties of accessing appropriate aryl Au(iii) models and the use of a strong external oxidant that complicates the catalytic system.

**Scheme 1:**
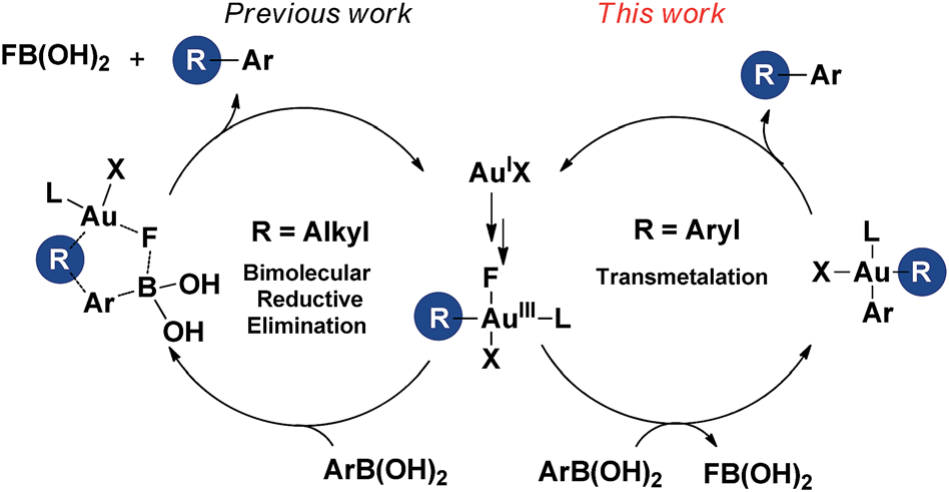
Reactivities of alkylgold(iii) (left) and arylgold(iii) (right) species with arylboronic acid.

In 2008, the seminal work of Gouverneur and co-workers demonstrated the potential of electrophilic fluorinating reagents in oxidizing Au(i) to Au(iii) species.^10^ Subsequently, substantial progress has been made in the gold-catalyzed oxidative heteroarylation (*e.g.* oxyarylation and aminoarylation) of alkenes with various arylating reagents, using electrophilic fluorinating reagents as oxidants,^11^ in which alkylgold(iii) fluorides have been proposed as the intermediates. These gold(iii) species undergo a bimolecular reductive elimination with arylboronic acid (*devoid of transmetallation*) by a concerted process wherein B–F and C–C bonds are formed simultaneously (Scheme 1, left).^11a,11c,11e,12^ In this work, we wish to establish well-defined aryl gold(iii) models, to comprehensively understand their elementary reactivities with arylboronic acids and gain further insight into the mechanism of gold-catalyzed oxidative C(sp^2^)–H arylation, which will benefit the maturation of catalytic biaryl couplings involving a gold(i)/gold(iii) redox cycle.

Gold actually has a rich history in organometallic chemistry. As early as 1931, a seminal work by Kharasch and Isbell illustrated the C–H bond activation of arenes with gold(iii) chloride to form aryl gold(iii) complexes.^13^ Thereafter, a variety of aryl gold(iii) complexes were prepared by C–H bond activation^14,15^ and transmetallation reactions with Au(iii) species.^16^ Inspired by chelation-assisted transition-metal catalyzed C–H bond arylation of arenes,^17,18^ we envisaged that the installation of a coordinating group on the aromatic ring would help us further understand the fundamental chemistry of aryl gold(iii) reactivity, particularly involving redox cycling. The introduction of a coordinating unit would have the following advantages: (1) the extra chelation could help activate the C(sp^2^)–H bond to form a cyclometalated Au(iii) complex and synchronously stabilize the resulting Au(iii) species; and (2) more importantly, the fixed coordinating group may partially avoid associative ligand/anion exchange, facilitating discrimination of the active Au(iii) species. Thus, the well-defined aryl cycloaurated complexes would allow us to gain more insights into the mechanism of transformations.

## Results and discussion

**Fig. 1:**
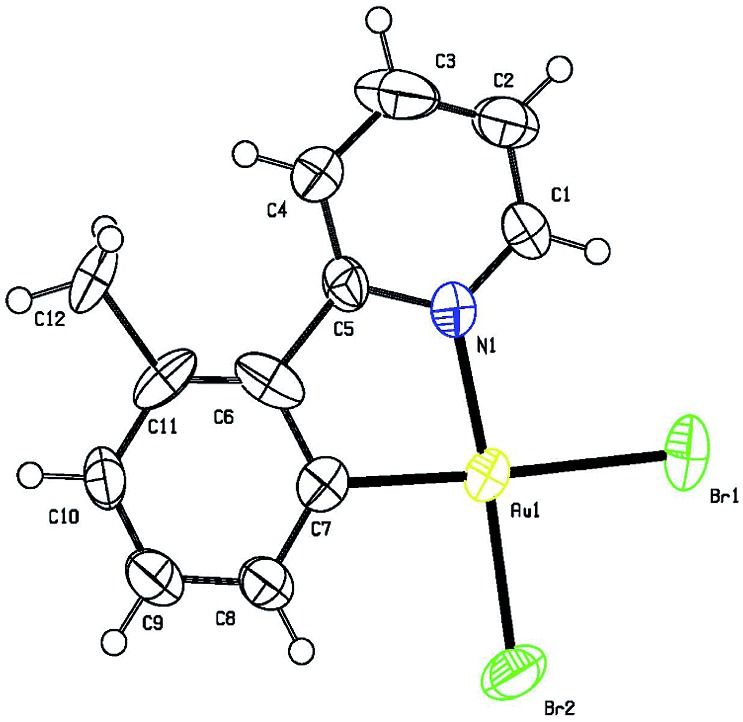
ORTEP diagram of **3b**. Thermal ellipsoids are shown at the 50% probability level.

The gold-catalyzed aryl C–H bond functionalization *via* a chelation-assisted strategy has never been reported before. Given that the 2-phenylpyridine derivatives are the most commonly used scaffolds for investigating transition metal-catalyzed directed C–H activation of arenes,^17a–c,e–f^ we herein took 2-(*o*-tolyl)pyridine **1a** as a molecular model to illuminate both the stoichiometric and catalytic reactivities^19^ of aryl gold(iii) species. Dihaloauracycles like [Au(py)X_2_] can be easily prepared by the cycloauration of the corresponding arenes with HAuCl_4_ or NaAuCl_4_ by Constable and Leese,^20^ and later by other researchers.^21^ By a modification of the above methods, stoichiometric AuX_3_ (X = Cl, Br) was first chosen to react with **1a** in *t*-BuOH at ambient temperature. The resulting coordination compounds **2a** and **2b** were heated in a mixture of acetonitrile and water at 130 °C, affording the five-membered *cis*-aryl gold(iii) complexes [Au(tpy)X_2_] **3a** and **3b**, respectively (eqn (1)). The cyclometalated Au(iii) structure of **3b** was demonstrated by X-ray crystallographic analysis (Fig. 1).† Similar to other cycloaurated [Au(C^N)Cl_2_] species,^16a,22^ complexes **3a** and **3b** are exceedingly stable towards water and air.1
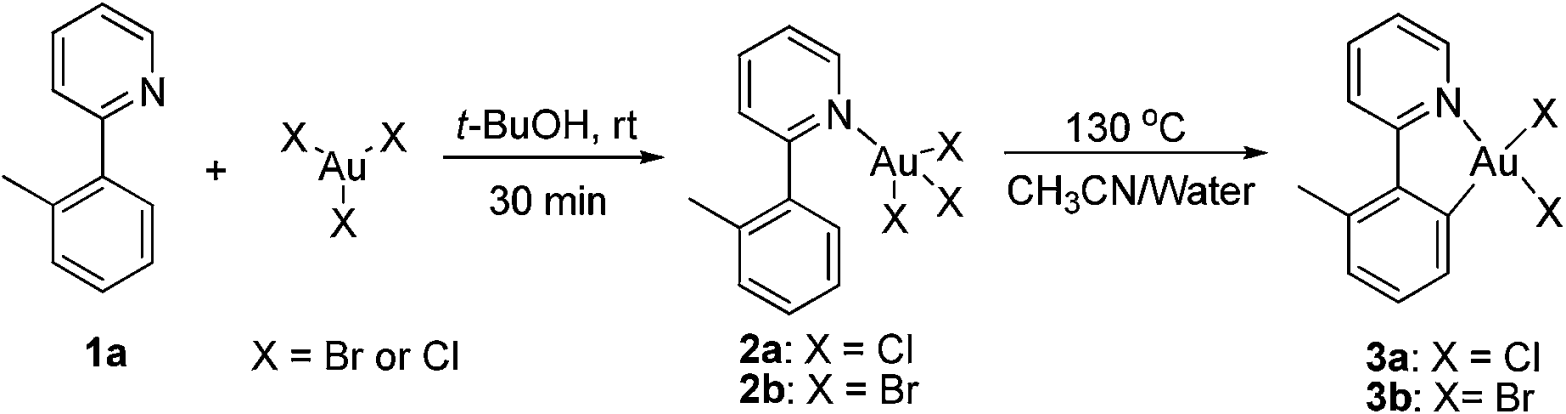



**Fig. 2:**
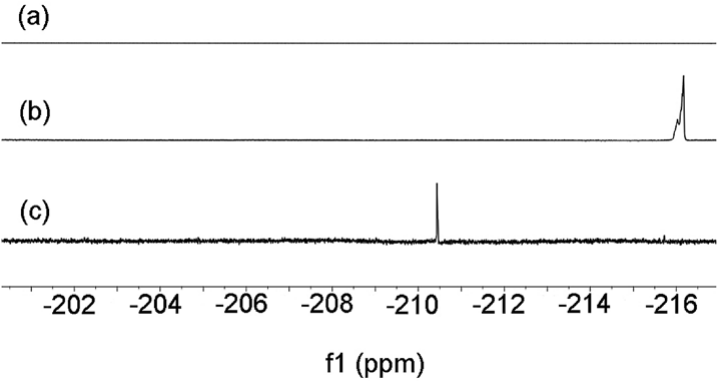
The ^19^F NMR spectra of (a) a mixture of **3a** and KF in DMSO-*d*_6_ in the presence or absence of phenylboronic acid, (b) a mixture of AuCl and NSFI in DMSO-*d*_6_, and (c) the catalytic reaction system in DMSO-*d*_6_.

With the isolated monometallic *cis*-aryl gold(iii) complexes **3a** and **3b** in hand, we wish to explore both stoichiometric and catalytic reactivities with arylboronic acids. A series of stoichiometric experiments was first conducted. The *cis*-aryl gold(iii) complexes **3a** and **3b** proved to be unreactive upon treatment with phenylboronic acid in *t*-BuOH at 130 °C. Encouraged by the role of fluoride in the gold-catalyzed oxidative heteroarylation of alkenes,^11^ potassium fluoride was added to the reaction mixture. Fortunately, the desired product **4aa** was obtained in excellent yields (for **3a**, 89%; for **3b**, 94%) (eqn (2)). To clarify the role of the fluoride anion, we subsequently investigated the reaction of **3a** with phenylboronic acid in the presence of either *t*-BuOK or KOH instead of KF. The coupled product **4aa** was obtained in 84% and 87% yields in *t*-BuOK and KOH, respectively. The ^19^F NMR spectra of a mixture of **3a** and KF, either in the presence or absence of phenylboronic acid in DMSO-*d*_6_ solution, did not exhibit the presence of a Au(iii)–F bond, which indicated that anion exchange between Cl^–^ or Br^–^ and F^–^ did not occur (Fig. 2a). On the basis of the above observations, we rationalized that the fluoride anion might perform as a Lewis base to cooperatively activate the C–B bond and further promote the cleavage of the C–B bond in the transmetallation, which was similar to that of the transition metal-catalyzed oxidative coupling reactions involving arylboronic acids.^23^
2
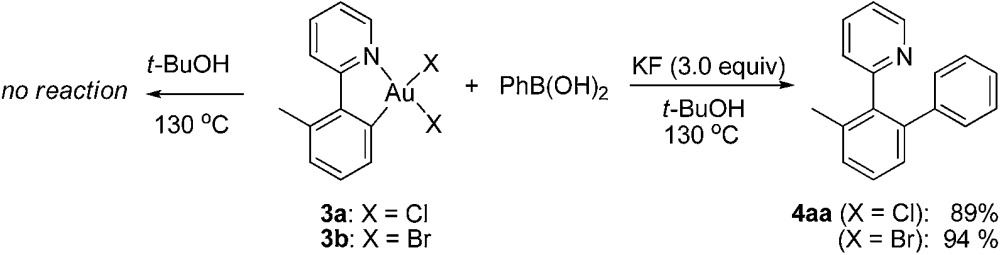

3
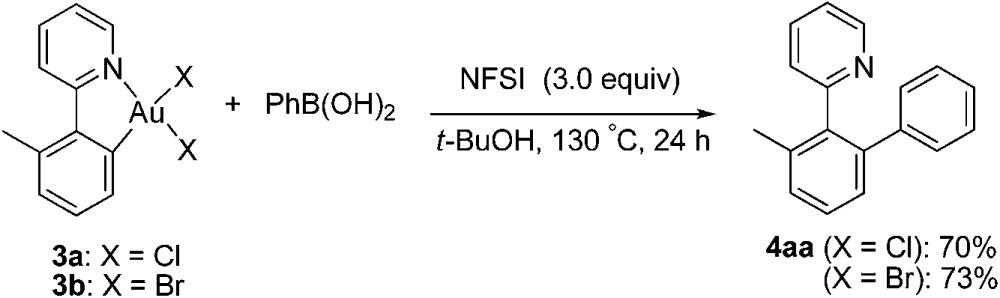

4
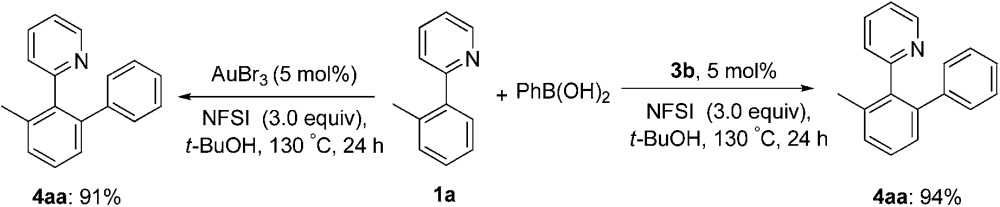



Next, we attempted to develop a catalytic version by using an external oxidant to access a gold(i)/gold(iii) catalytic cycle. It is known that electrophilic fluorinating reagents and iodine(iii) oxidants could efficiently accomplish the oxidation of Au(i) to Au(iii). In combination with the role of the fluoride anion as a Lewis base mentioned above, we herein concentrated on the fluorine-containing oxidants. The stoichiometric gold(iii) complexes **3a** and **3b** could react with phenylboronic acid (3.0 equiv.) to form the biaryl **4aa** in 70% (X = Cl) and 73% (X = Br) yields in the presence of *N*-fluorobenzenesulfonimide (NFSI) (eqn (3)), whereas PhI(OAc)_2_ failed to promote the coupling reaction (Table S1,† entry 11). Treatment of **3b** with phenylboronic acid and Selectfluor also produced **4aa** in 72% yield. Importantly, both the cyclometalated gold(iii) complex **3b** (5 mol%) and AuBr_3_ (5 mol%) could catalyze the reaction between **1a** and phenylboronic acid to form the cross-coupled **4aa** in 94% and 91% yields, respectively, which hinted that the five-membered aryl gold(iii) species might be a plausible intermediate in the catalytic cycle (eqn (4)). In addition, the yield of **4aa** was reduced to 58% in the presence of AuBr_3_ (5 mol%) when the amounts of phenylboronic acid were decreased from 3.0 equiv. to 1.0 equiv. GC-MS analysis of the stoichiometric reaction of **3a** with PhB(OH)_2_ in the presence of NFSI showed the formation of biphenyl. Further ^19^F NMR spectrum analysis of a mixture of NFSI and *t*-BuOH demonstrated a new signal at –39.3 ppm (^19^F NMR of NSFI: –169.2 ppm). These observations implied the fluoride equivalents might be produced from the reduction of NFSI by phenylboronic acid and/or *t*-BuOH in the reaction system.

**Fig. 3:**
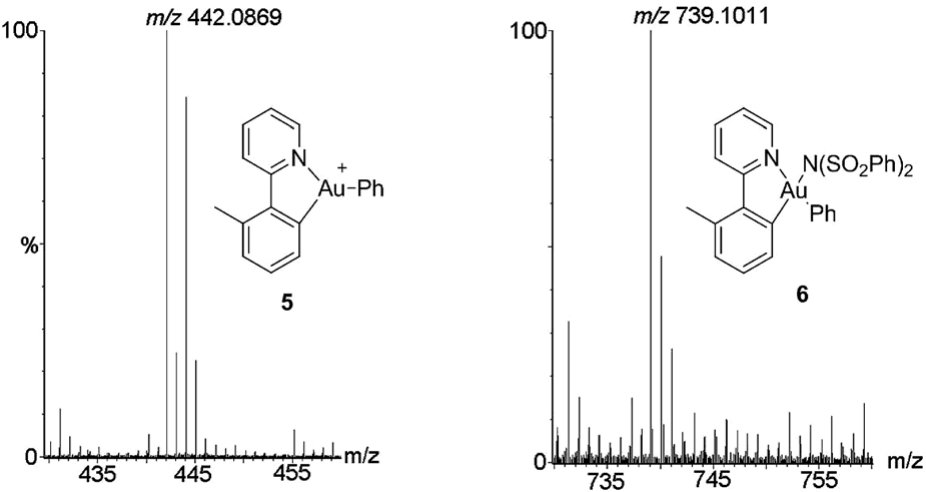
The ESI-HRMS analysis of the AuBr_3_-catalyzed reaction of **1a** with phenylboronic acid.

**Fig. 4:**
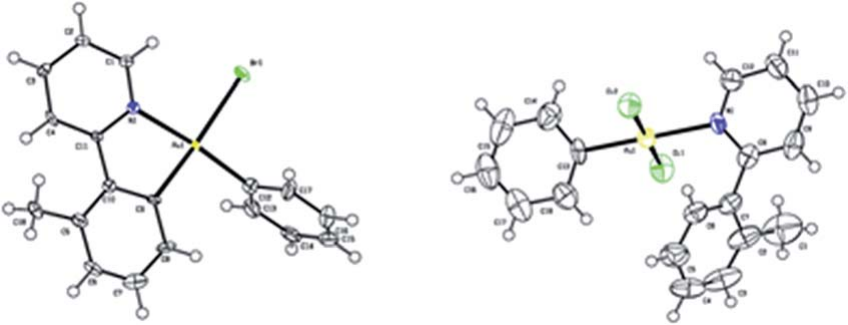
ORTEP diagrams of **7** (left) and **8** (right). Thermal ellipsoids are shown at the 50% probability level.

To further clarify the biaryl gold(iii) intermediates, the ESI-HRMS analysis of the reactions of **3b** or **1a**/AuBr_3_ with phenylboronic acid was performed in the presence of NSFI. Fortunately, the peaks at *m*/*z* 442.0869 and 739.1011, respectively, corresponding to the characteristic patterns of the five-membered biaryl gold(iii) species **5** ([M]^+^, MW 442.0870) and **6** ([M + H]^+^, MW 739.1000) were observed,^24^ which suggested a transmetallation process from boronic acid to gold(iii) (Fig. 3). Despite this evidence, attempts to separate the intermediate from the reaction system failed, probably due to the rapid reductive elimination at 130 °C. As an alternative, we turned to the synthesis of the cyclometalated biaryl gold(iii) species [AuBr(Ph)(tpy)] (**7**), allowing direct observation of the reaction intermediate. By a modified Tilset method,^25^ the sequential reaction of **1a** with Au(OAc)_3_ and PhMgBr afforded complex **7**, which was determined by single crystal X-ray diffraction (Fig. 4).† [AuBr(Ph)(tpy)] could give the hetero-coupled product **4aa** in 98% yield in *t*-BuOH at 80 °C (eqn (5)), showing that the biaryl reductive elimination step was similar to those of traditional palladium and rhodium-catalyzed biaryl coupling reactions. This observation also clearly disclosed that the presence of the Au(iii)–F bond was not indispensable in the reductive elimination step and was distinguished from the reactivity of alkylgold(iii) species with arylboronic acid (Scheme 1, left). Considering that the catalytic reaction required a high reaction temperature of 130 °C (eqn (4)) and failed to give the arylated product **4aa** at 80 °C, it was suggested that reductive elimination might not be the rate-determining step.5
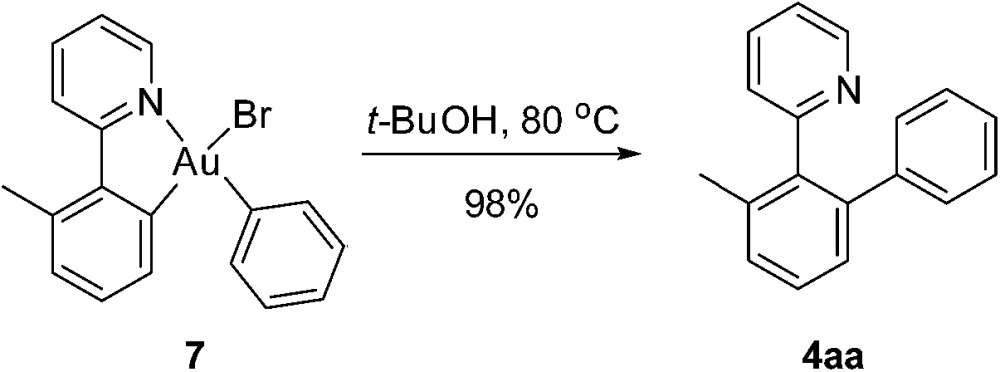



As the gold-catalyzed cross-coupling reaction between an arene and an arylboronic acid was demonstrated, this raised the question of whether the arene C–H auration preceded the C–B auration or *vice versa*. The reaction of [AuCl_2_(Ph)(tpy)] (**8**) could not discriminate between the two. The gold(iii) complex **8** could be prepared by the modified method of Fuchita *et al.* ^15*d*^ and Limbach *et al.*,^26^ involving the auration of benzene with AuCl_3_ and subsequent coordination with **1a**. The structure of complex **8** was demonstrated by X-ray crystallographic analysis (Fig. 4).† As a result, the stoichiometric reaction of **8** in *t*-BuOH did not afford the cross-coupled product either in the presence or absence of NFSI (eqn (6)). These results not only indicated that the C–B auration occurred after cyclometallation, but also precluded the possibility of C–B activation before the Au(i) was oxidized to Au(iii).6
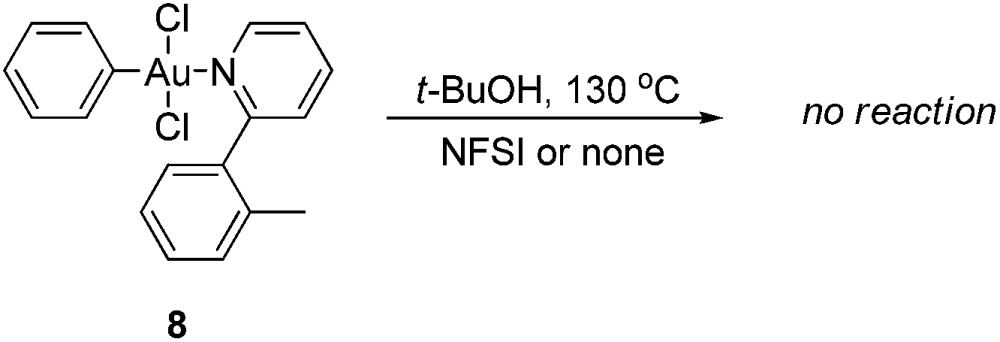



To elucidate whether a Au(iii)–F bond was formed in the catalytic cycle, the ^19^F NMR spectra were investigated. The ^19^F NMR spectrum of a mixture of AuCl and NSFI in *t*-BuOH at 130 °C exhibited a signal of –216.2 ppm (Fig. 2b). Indeed, AuCl (5.0 mol%) as the pre-catalyst could promote the reaction of **1a** with phenylboronic acid to produce **4aa** albeit in a lower yield of 38%. The catalytic system of **1a** and PhB(OH)_2_ showed a ^19^F NMR peak at 210.4 ppm (Fig. 2c). These results suggested that the Au(iii)–F bond might be generated in the catalytic reaction.^27^

Subsequently, the H/D exchange experiments were performed. Exposure of 2-(*o*-tolyl)pyridine **1a** to *t*-BuOD did not lead to the deuterated product, either in the presence or absence of phenylboronic acid, which indicated that the directed cleavage of the *ortho* C–H bond was actually an irreversible process (eqn (7)). A small H/D kinetic isotope effect (KIE) of 0.96 for two separate reactions with **1a** and [D]-**1a** revealed that the C(sp^2^)–H bond cleavage was not involved in the rate-determining step (eqn (8)).^28^ The observed *k*_H_/*k*_D_ value implied that the formation of a C–H(D) sigma–agostic complex might be rate limiting.^29^
7
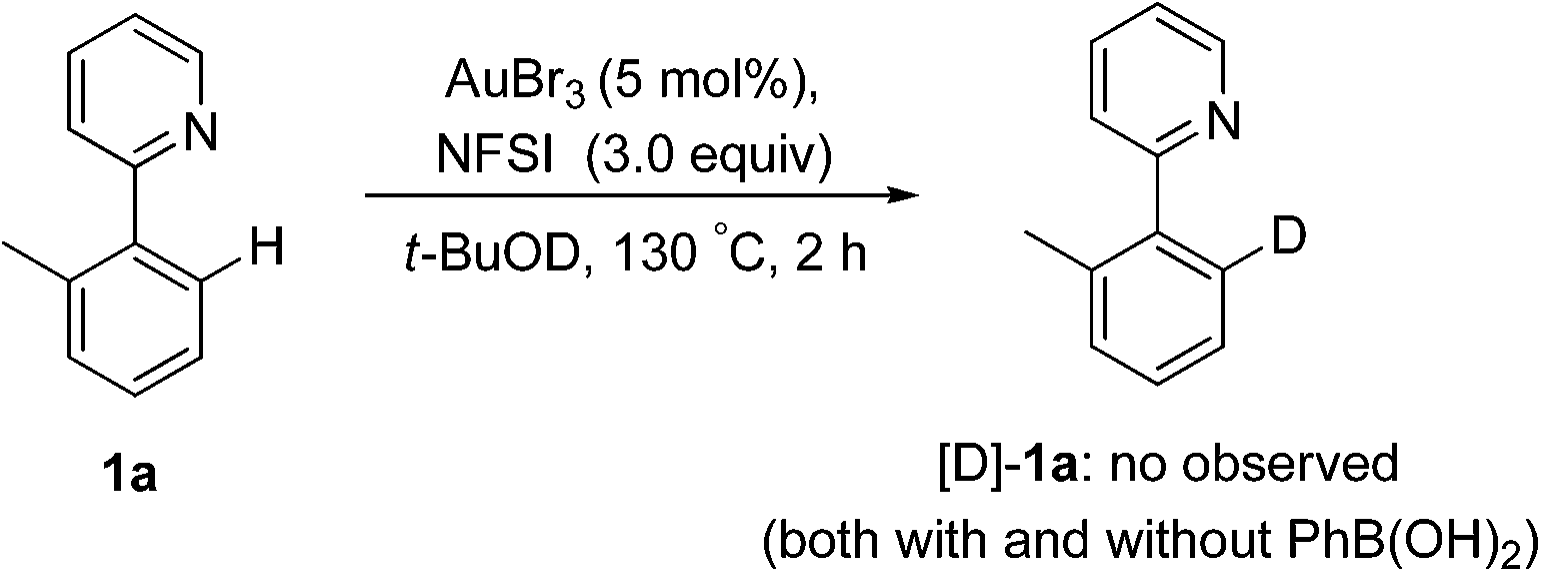

8
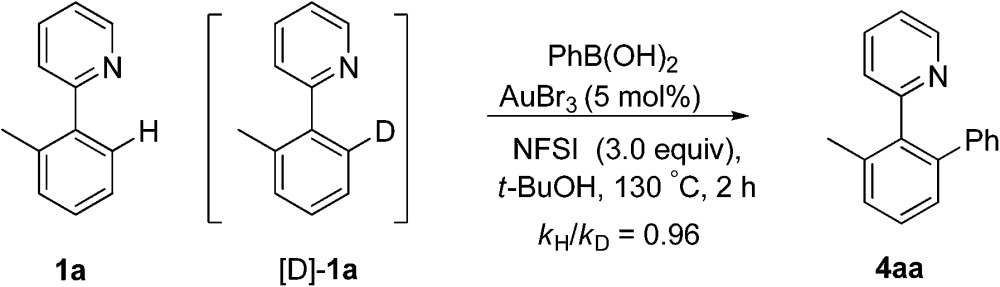



**Scheme 2:**
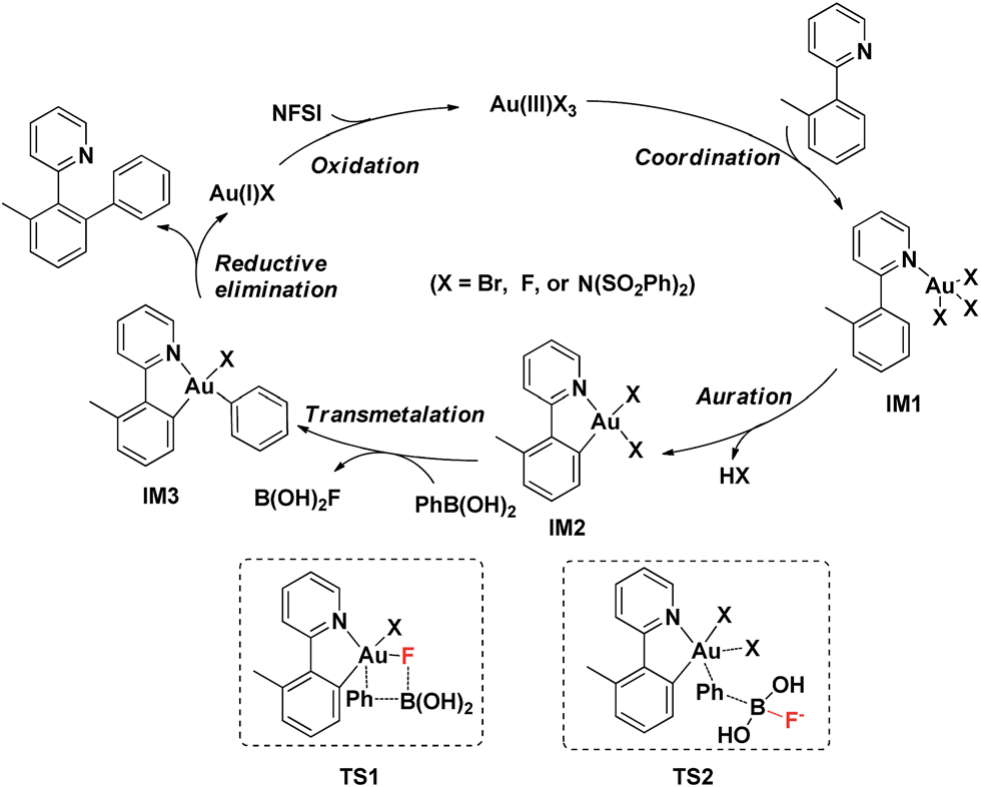
Proposed mechanism of gold(iii)-catalyzed C(sp^2^)–H bond arylation with arylboronic acid *via* a chelation-assisted strategy.

Based on the above observations, a plausible mechanistic pathway of the gold(iii)-catalyzed directed C–H bond arylation of an arene with an arylboronic acid was proposed. First, the intermediate **IM1** was formed by the rapid coordination of a gold(iii) species with **1a**, followed by an irreversible C–H bond auration to give the five-membered cycloaurated species **IM2**. The transmetallation from boronic acid to gold(iii) center might involve both scenarios **TS1** and **TS2**.^16d,16e,30^ In **TS1**, the Au(iii)–F bond formed *via* an oxidation of Au(i)X by NFSI^11a–c,12^ activated the C–B bond through a four-membered transition state (Fig. 2, right). In **TS2**, the non-coordinated fluoride anion promoted the cleavage of the C–B bond. In the coupling reaction, NFSI not only served as a strong oxidant, but also offered a fluoride source to promote the transmetallation process. The biaryl gold(iii) intermediate **IM3** underwent a reductive elimination to generate the desired biaryl product. Finally, the released gold(i) species was reoxidized by NFSI to complete the catalytic cycle (Scheme 2).

**Scheme 3:**
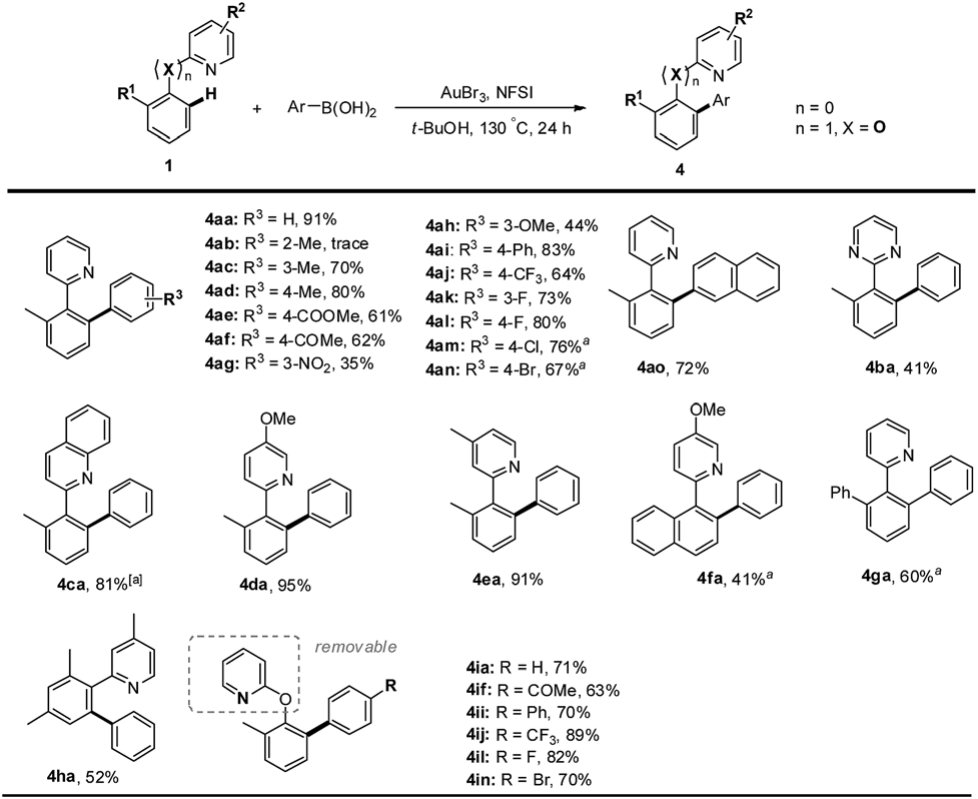
AuBr_3_-catalyzed directed C–H arylation of arenes with arylboronic acids. Reaction conditions: biaryl or biaryl ether (0.2 mmol), arylboronic acid (3.0 equiv.), gold(iii) bromide (5 mol%), and NFSI (3.0 equiv.) in *t*-BuOH (2 mL) at 130 °C under N_2_ for 24 h. ^*a*^At 140 °C.

Finally, we sought to examine the scope of this protocol with respect to both arylboronic acids and arenes (Scheme 3).^31^ We were pleased to observe that a series of *meta*- or *para*-substituted phenylboronic acids smoothly reacted with 2-(*o*-tolyl)pyridine **1a** to deliver the heterocoupled products in medium to excellent yields. However, the *ortho*-substituted phenylboronic acid gave a trace amount of product, presumably due to the steric effect (**4ab**). Furthermore, arylboronic acids bearing either electron-rich (**4ac**, **4ad** and **4ah**) or electron-deficient (**4ae–ag** and **4aj–al**) substituents proceeded smoothly. Other biaryls containing *N*-heteroarenes such as pyridine, quinoline and pyrimidine also underwent cross-coupling reactions with phenylboronic acid. The gold(iii)-catalyzed oxidative reaction was very tolerant of halogen groups such as Cl and Br (**4am**, **4an** and **4in**). In addition to heteroaryl-aryls proposed to form a five-membered auracycle, 2-(*o*-tolyloxy)pyridine also went through the arene C–H arylation with a variety of arylboronic acids (**4ia–4in**), which might involve a six-membered auracycle. The pyridyl group of the resulting biaryls could be removed to afford the 1,1′-biphenyl-2-ol derivatives.^32^

## Conclusions

In summary, taking advantage of chelation-assisted C–H bond activation, we have established the well-defined five-membered aryl gold(iii) complexes [Au(tpy)X_2_] (**3a** and **3b**) and [AuBr(Ph)(tpy)] (**7**), as well as the aryl gold(iii) complex [AuCl_2_(Ph)(tpy)] (**8**), as models for a comprehensive insight into the mechanism of gold-catalyzed oxidative C–H arylation of arenes. The stoichiometric behaviors of the complexes with arylboronic acids provide straightforward access to propel our nascent understanding of the immature redox chemistry of gold catalysts. Observable arene C–H activation, transmetallation and biaryl reductive elimination have provided direct evidence for a mechanistic hypothesis. In this work, the chelation-assisted C–H activation strategy has been used for the development of the gold(iii)-catalyzed oxidative cross-coupling reactions between arenes and arylating reagents. Compared with the works of Lloyd-Jones and Russell,^7,8^ the present work demonstrates the possibility of a different catalytic cycle in which (1) the auration reaction step could be replaced by a cycloauration reaction if the arene has an appropriate *ortho*-substituent, (2) the arylboronic acids can be used instead of aryl silanes as transmetallating agents, and (3) the oxidant can be NFSI instead of iodine(iii) oxidant.
