# Supplementary material for: Stoichiometric to catalytic reactivity of the aryl cycloaurated species with arylboronic acids: insight into the mechanism of gold-catalyzed oxidative C(sp2)–H arylation
Source: Chem Sci. 2014 Oct 9;6(1):288–93. doi: 10.1039/c4sc02070g (PMC5463920; doi:10.1039/c4sc02070g)
Supplement: Supplementary file 1 [file SC-006-C4SC02070G-s001.pdf]

Electronic Supplementary Material (ESI) for Chemical Science.
This journal is © The Royal Society of Chemistry 2014

Supporting Information

From stoichiometric to catalytic reactivity of the aryl cycloaurated species with arylboronic acids: insight into the mechanism of gold-catalyzed oxidative C(sp^2)–H arylation

Qian Wu, Chenglong Du, Yumin Huang, Xingyan Liu, Zhen Long, Feijie Song and Jingsong You*

Key Laboratory of Green Chemistry and Technology of Ministry of Education, College of Chemistry, and State Key Laboratory of Biotherapy, West China Medical School, Sichuan University, 29 Wangjiang Road, Chengdu 610064, PR China

Fax: 86-28-85412203; E-mail: jsyou@scu.edu.cn

Table of Contents

I. General remarks S3

II. Synthesis of gold(III) complexes and additional experiments...............................S3

III. ESI-HRMS detection...S11

IV. Kinetic isotope effect experiments S11

V. ORTEP diagrams of **3b**, **7** and **8** S15

VI. General procedure for gold-catalyzed directed C(sp^2)–H bond arylation with arylboronic acid S16

VII. Experimental data for the described substances S16

VIII. Removal of the directing group...S32

IX. References. S32

X. Copies of ^{1}H and ^{13}C NMR spectra. S33

I. General remarks

NMR spectra were obtained with a Bruker AV II-400 spectrometer. The ^{1}H NMR (400 MHz) chemical shifts and the ^{13}C NMR (100 MHz) chemical shifts were measured relative to $CDCl_3$ (H: δ = 7.26 ppm; C: δ = 77.16 ppm), DMSO-d_6 (H: δ = 2.50 ppm; C: δ = 39.52 ppm), and acetone-d_6 (H: δ = 2.05 ppm; C: δ = 29.84 ppm) as the internal references. High-resolution mass spectra (HRMS) were obtained with a Waters-Q-TOF-Premier (ESI). GC-Mass spectra were obtained with a Shimadzu-GCMS-QP 2010 SE (EI). X-Ray single-crystal diffraction data were collected on Oxford Xcalibur E and Xcalibur Gemini X-ray single crystal diffractometers. Elemental analysis data were obtained on EA FLASH 1112 SERIES. Melting points were determined with XRC-1 and are uncorrected.

Unless otherwise noted, all reagents were obtained from commercial suppliers and used without further purification. $AuCl_3$, $AuBr_3$, and $Au(OAc)_3$ were purchased from Across, Alfa Aesar and Adamas, respectively. 2-(*o*-Tolyl)quinoline,[1] arylpyridine derivatives,[2] and 2-(*o*-tolyloxy)pyridine[3] were prepared according to the literature procedure. Solvents were dried by refluxing over CaH_2 (for DCE), or sodium (for 1,4-dioxane, *t*-BuOH, *t*-AmylOH, diethyl ether, benzene, hexane, and THF), and freshly distilled prior to use. All syntheses and manipulations were carried out under N_2 atmosphere.

II. Synthesis of gold(III) complexes and additional experiments

(1) Synthesis of cyclometalated aryl gold(III) halides 3a and 3b[4]

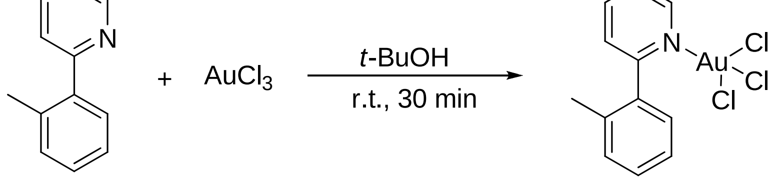

1a **2a**

To a solution of 2-(*o*-tolyl)pyridine (36.3 mg, 0.22 mmol) in *t*-BuOH (2 mL) was added $AuCl_3$ (60.3 mg, 0.2 mmol), and the yellow precipitate was formed immediately. This precipitate was filtered off, washed with petroleum ether, and dried in vacuum to afford the **2a** as a yellow solid in 93% yield (87.6 mg). M.p.: 134-136 °C. ^{1}H NMR (acetone-d_6, 400 MHz): δ = 2.28 (s, 3H), 7.44-7.48 (m, 2H), 7.55 (td, J = 7.6 Hz, J = 1.6 Hz, 1H), 7.71 (d, J = 7.6 Hz, 1H), 8.02-8.06 (m, 2H), 8.46 (dd, J = 7.6 Hz, J = 1.2 Hz, 1H), 9.30 (dd, J = 6.8 Hz, J = 1.2 Hz, 1H) ppm. ^{13}C NMR (acetone-d_6, 100 MHz): δ = 20.7, 126.9, 128.1, 131.0, 131.8, 132.1, 137.8, 143.3, 151.6, 160.1 ppm. Anal. Calcd for $C_{12}H_{11}AuCl_3N$ (%): C, 30.50; H, 2.35; N, 2.96, found: C, 30.36; H, 2.24; N, 2.90.

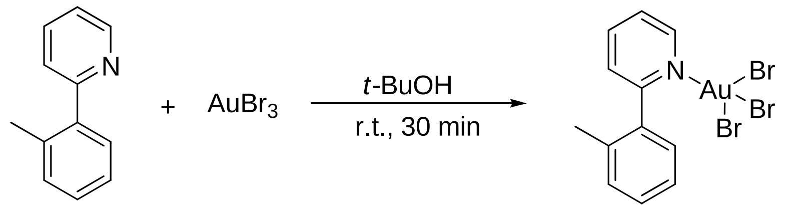

1a **2b**

To a solution of 2-(*o*-tolyl)pyridine (36.3 mg, 0.22 mmol) in *t*-BuOH (2 mL) was added $AuBr_3$ (86.7 mg, 0.2 mmol), and the red precipitate was formed immediately. This precipitate was filtered off, washed with petroleum ether, and dried in vacuum to afford the **2b** as a red solid in 88% yield (106.1 mg). M.p.: 187-189 °C. ^{1}H NMR (acetone-d_6, 400 MHz): δ = 2.31 (s, 3H), 7.42-7.44 (m, 2H), 7.51-7.55 (m, 1H), 7.80-7.82 (m, 1H), 7.98-8.01 (m, 2H), 8.43 (td, J = 8.0 Hz, J = 1.6 Hz, 1H), 9.23 (dd, J = 6.0 Hz, J = 0.8 Hz, 1H) ppm. ^{13}C NMR (acetone-d_6, 100 MHz): δ = 21.1, 126.7, 127.7, 131.0, 131.6, 131.8, 132.3, 137.6, 142.9, 152.3, 160.1 ppm. Anal. Calcd for $C_{12}H_{11}AuBr_3N$ (%): C, 23.79; H, 1.83; N, 2.31, found: C, 23.79; H, 1.50; N, 2.09.

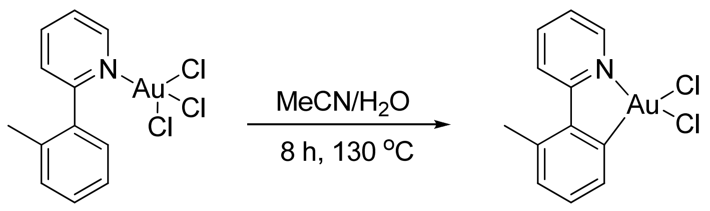

2a **3a**

A sealed tube with a magnetic stirring bar was charged with **2a** (47.1 mg, 0.1 mmol), MeCN (2.0 mL) and H_2O (2.0 mL) under N_2. A rubber septum was replaced with Teflon stopper, and the system was evacuated twice and back filled with N_2. Then the reaction mixture was stirred at 130 °C for 8 h. The white solid was filtered off, washed with the mixture of MeCN and H_2O (1/1, v/v), and dried in vacuum to afford the **3a** as a white solid in 53% yield (23.1 mg). M.p.: >250 °C. ^{1}H NMR (DMSO-d_6, 400 MHz): δ = 2.74 (s, 3H), 7.24 (t, J = 7.6 Hz, 1H), 7.34 (d, J = 7.2 Hz, 1H), 7.76-7.82 (m, 2H), 8.34-8.42 (m, 2H), 9.72 (dd, J = 6.0 Hz, J = 1.2 Hz, 1H) ppm. ^{13}C NMR (DMSO-d_6, 100 MHz): δ = 22.9, 124.7, 125.4, 128.0, 130.2, 133.4, 138.2, 140.5, 143.9, 148.9, 152.8, 163.9 ppm. HRMS (ESI$^+$): calcd for $C_{12}H_{10}AuClN$ $[M-Cl]^+$ 400.0167, found 400.0163. Anal. Calcd for $C_{12}H_{10}AuCl_2N$ (%): C, 33.05; H, 2.31; N, 3.21, found: C, 32.78; H, 2.43; N, 3.34.

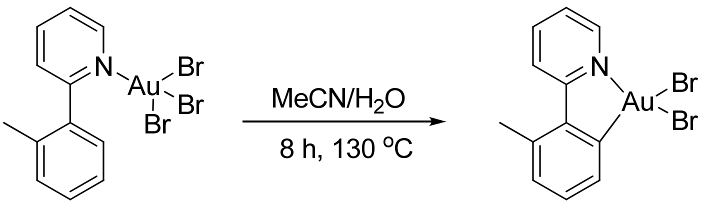

2b **3b**

A sealed tube with a magnetic stirring bar was charged with **2b** (60.3 mg, 0.1 mmol), MeCN (2.0 mL) and H_2O (2.0 mL) under N_2. A rubber septum was replaced with Teflon stopper, and the system was evacuated twice and back filled with N_2. Then the reaction mixture was stirred at 130 °C for 8 h. The white solid was filtered off, washed with the mixture of MeCN and H_2O (1/1, v/v), and dried in vacuum to afford the **3b** as a white solid in 34% yield (17.8 mg). M.p.: 225-227 °C. ^{1}H NMR (DMSO-d_6, 400 MHz): δ = 2.74 (s, 3H), 7.21 (t, J = 8.0 Hz, 1H), 7.34 (d, J = 7.2 Hz, 1H), 7.79 (t, J = 6.4 Hz, 1H), 8.14 (d, J = 8.0 Hz, 1H), 8.34-8.42 (m, 2H), 9.94 (d, J = 5.6 Hz, 1H) ppm. ^{13}C NMR (DMSO-d_6, 100 MHz): δ = 23.2, 124.8, 125.6, 130.2, 130.5, 133.2, 138.3, 141.2, 143.6, 150.0, 154.0, 163.9 ppm. HRMS (ESI$^+$): calcd for $C_{12}H_{10}AuBrN$ $[M-Br]^+$ 443.9662, found 443.9663. Anal. Calcd for $C_{12}H_{10}AuBr_2N$ (%): C, 27.45; H, 1.92; N, 2.67, found: C, 27.75; H, 1.58; N, 2.46.

(2) Reactivities of 3a and 3b with $PhB(OH)_2$

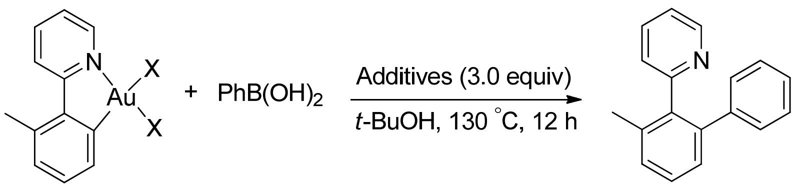

3a: X = Cl
3b: X = Br

4aa

A sealed tube with a magnetic stirring bar was charged with **3a** or **3b** (0.05mmol), phenylboronic acid (18.3 mg, 0.15 mmol, 3.0 equiv), additive (3.0 equiv), and *t*-BuOH (1 mL) under N_2. A rubber septum was replaced with Teflon stopper, and the system was evacuated twice and back filled with N_2. Then the reaction mixture was stirred at 130 °C for 12 h. The reaction mixture was then cooled to ambient temperature, diluted with 10 mL of CH_2Cl_2, filtered through a Celite pad, and washed with 10-20 mL of CH_2Cl_2. The combined organic extracts were concentrated and the resulting residue was purified by neutral alumina column chromatography (petroleum ether/acetone = 30:1, v/v) to provide product **4aa** as a white solid. ^{1}H NMR ($CDCl_3$, 400 MHz): δ = 2.20 (s, 3H), 6.89 (d, J = 7.6 Hz, 1H), 7.07-7.10 (m, 3H), 7.12-7.17 (m, 3H), 7.26-7.31 (m, 2H), 7.37 (t, J = 7.6 Hz, 1H), 7.44 (td, J = 7.6 Hz, J = 1.6 Hz, 1H), 8.63 (d, J = 4.8 Hz, 1H) ppm. ^{13}C NMR ($CDCl_3$, 100 MHz): δ = 20.6, 121.4, 125.7, 126.3, 127.68, 127.69, 128.1, 129.5, 129.7, 135.8, 136.8, 139.4, 141.4, 141.8, 148.9, 159.7 ppm. M.p.: 49 °C. HRMS (ESI$^+$): calcd for $C_{18}H_{16}N$ $[M+H]^+$ 246.1283, found 246.1284.

Table S1: Stoichiometric reaction of **3a** or **3b** with $PhB(OH)_2$.[a]

**Entry**	**X**	**Additive**	**Yield (%)**[b]
1	Cl	-	NR
2	Cl	*t*-BuOK	84%
3	Cl	KOH	87%
4	Cl	KF	89%
5[c]	Cl	NFSI	70%
6[d]	Cl	NFSI	NR
7	Br	-	NR

8	Br	KF	94%
9[c]	Br	NFSI	73%
10[c]	Br	Selectfluor	72%
11[c]	Br	$PhI(OAc)_2$	NR

[a] Reaction conditions: **3a** or **3b** (0.05 mmol), phenylboronic acid (0.15 mmol, 3.0 equiv), additive (0.15 mmol, 3.0 equiv), and *t*-BuOH (1.0 mL) at 130 °C for 12 h under N2. [b] Yields of isolated products. [c] For 24 h. [d] At 60 °C. NR = no reaction. Selectfluor = 1-chloromethyl-4-fluoro-1,4-diazoniabicyclo[2.2.2]octane bis(tetrafluoroborate). NFSI = *N*-Fluorobenzenesulfonimide.

(3) Gold-catalyzed reactions of 2-(*o*-tolyl)pyridine (1a) with phenylboronic acid

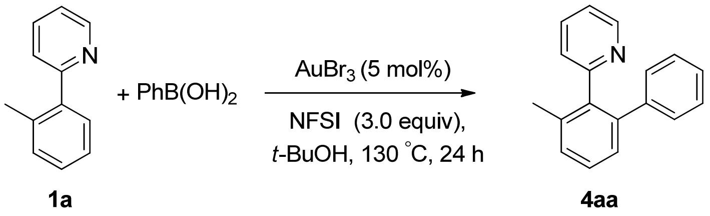

A sealed tube with a magnetic stirring bar was charged with 2-(*o*-tolyl)pyridine **1a** (33.8 mg, 0.2 mmol), phenylboronic acid (73.2 mg, 0.6 mmol, 3.0 equiv), gold(III) bromide (4.4 mg, 0.01 mmol, 0.05 equiv), NFSI (189.2 mg, 0.6 mmol, 3.0 equiv), and *t*-BuOH (2 mL) under N_2. A rubber septum was replaced with Teflon stopper, and the system was evacuated twice and back filled with N_2. Then the reaction mixture was stirred at 130 °C for 24 h. The reaction mixture was then cooled to ambient temperature, diluted with 10 mL of CH_2Cl_2, filtered through a Celite pad, and washed with 10-20 mL of CH_2Cl_2. The combined organic extracts were concentrated and the resulting residue was purified by column chromatography on neutral alumina (petroleum ether/acetone = 30:1, v/v) to provide product **4aa** as a white solid in 91% yield (44.6 mg).

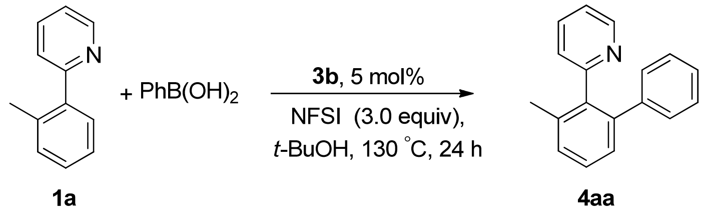

A sealed tube with a magnetic stirring bar was charged with 2-(*o*-tolyl)pyridine **1a** (33.8 mg, 0.2 mmol), phenylboronic acid (73.2 mg, 0.6 mmol, 3.0 equiv), **3b** (5.2 mg, 0.01 mmol, 0.05 equiv), NFSI (189.2 mg, 0.6 mmol, 3.0 equiv), and *t*-BuOH (2 mL) under N_2. A rubber septum was replaced with Teflon stopper, and the system was evacuated twice and back filled with N_2. Then the reaction mixture was stirred at 130 ^oC for 24 h. The reaction mixture was then cooled to ambient temperature, diluted with 10 mL of CH_2Cl_2, filtered through a Celite pad, and washed with 10-20 mL of CH_2Cl_2. The combined organic extracts were concentrated and the resulting residue was purified by neutral alumina column chromatography (petroleum ether/acetone = 30:1, v/v) to provide product **4aa** as a white solid in 94% yield (46.1 mg).

(4) Synthesis of cyclometalated biaryl gold(III) species 7[5]

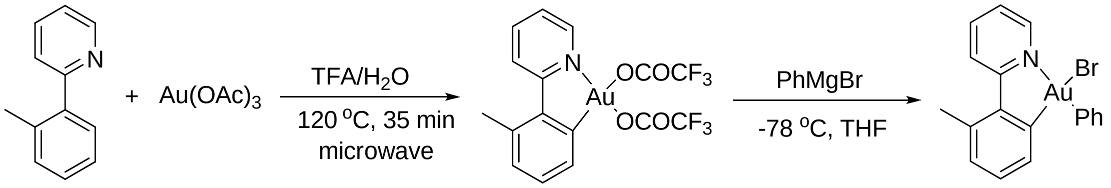

1a 7

A sealed tube with a magnetic stirring bar was charged with gold(III) acetate (262 mg, 0.7 mmol), 2-(*o*-tolyl)pyridine (125.5 mg, 0.74 mmol, 1.06 equiv), distilled water (10 mL) and trifluoroacetic acid (10 mL). Then the reaction mixture was heated in the microwave oven at 120 °C for 35 min. After the solution was cooled to room temperature, additional trifluoroacetic acid was added until the white precipitate was dissolved. Then the yellow solution was filtered through a Celite pad, and the product was precipitated by addition of water. The precipitated solid was filtered, washed with water and dried under vacuum to give $[Au(OCOCF_3)_2(tpy)]$ in 61% yield (252.4 mg). ^1H NMR (DMSO-d_6, 400 MHz): δ = 2.74 (s, 3H), 6.87 (d, J = 8.0 Hz, 1H), 7.24 (t, J = 8.0 Hz, 1H), 7.40 (d, J = 7.6 Hz, 1H), 7.76-7.80 (m, 1H), 8.34 (d, J = 8.0 Hz, 1H), 8.44-8.48 (m, 1H), 8.67 (dd, J = 6.0 Hz, J = 1.2 Hz, 1H) ppm. ^{13}C NMR (DMSO-d_6, 100 MHz): δ = 22.1, 125.0, 125.1, 125.8, 130.4, 134.1, 138.8, 139.5, 142.7, 145.1, 148.4, 158.7, 159.1, 163.2 ppm. HRMS (ESI$^+$): calcd for $C_{16}H_{10}AuF_6NNaO_4$ $[M+Na]^+$ 614.0077, found 614.0072.

A solution of $Au(OCOCF_3)_2$(tpy) (200.9 mg, 0.34 mmol) in dried THF (20 mL) at -78 °C was added PhMgBr (1.0 M in THF, 0.76 mL, 0.76 mmol, 2.2 equiv) under a flow of N_2. The reaction mixture was stirred for 1 h at -78 °C and then at room temperature for another hour. After the solvent was removed under vacuum, the resulting solid was dissolved in dichloromethane and washed with distilled water. The organic phase was dried over $MgSO_4$ and filtered through a pad of Celite. Then the dichloromethane was removed under vacuum to give cyclometalated biaryl gold(III) species **7** as an off-white solid in 83% yield (147.0 mg). M.p.: 159-161 °C. ^{1}H NMR (DMSO-d_6, 400 MHz): δ = 2.74 (s, 3H), 6.49 (d, J = 7.6 Hz, 1H), 7.05 (t, J = 8.0 Hz, 1H), 7.13 (d, J = 7.2 Hz, 1H), 7.19-7.23 (m, 3H), 7.37 (d, J = 6.8 Hz, 2H), 7.78 (t, J = 6.0 Hz, 1H), 8.31 (td, J = 8.4 Hz, J = 1.6 Hz, 1H), 8.37 (d, J = 8.0 Hz, 1H), 9.64 (d, J = 5.2 Hz, 1H) ppm. ^{13}C NMR (DMSO-d_6, 100 MHz): δ = 23.8, 124.5, 124.8, 125.3, 129.0, 130.0, 130.8, 132.3, 132.7, 137.3, 141.5, 142.1, 144.4, 149.4, 150.1, 161.5 ppm. HRMS (ESI$^+$): calcd for $C_{18}H_{15}AuN$ [M-Br]$^+$ 442.0870, found 442.0872. Anal. Calcd for $C_{18}H_{15}AuBrN$ (%): C, 41.40; H, 2.90; N, 2.68, found: C, 41.52; H, 3.23; N, 2.55.

(5) Reductive elimination of 7

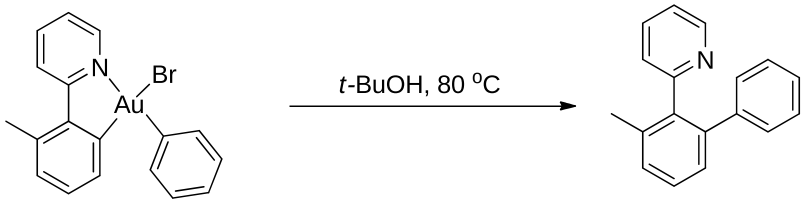

7 **4aa**

A sealed tube with a magnetic stirring bar was charged with **7** (26.1 mg. 0.05mmol) and *t*-BuOH (1 mL) under N_2. A rubber septum was replaced with Teflon stopper, and the system was evacuated twice and back filled with N_2. Then the reaction mixture was stirred at 80 °C for 12 h. The reaction mixture was then cooled to ambient temperature, diluted with 10 mL of CH_2Cl_2, filtered through a Celite pad, and washed with 10-20 mL of CH_2Cl_2. The combined organic extracts were concentrated and the resulting residue was purified by column chromatography on neutral alumina (petroleum ether/acetone = 30:1, v/v) to provide product **4aa** as a white solid in 98% yield or 96% yield in the presence of KF.

(6) Synthesis of [AuCl$_2$(Ph)(tpy)] (8)[6]

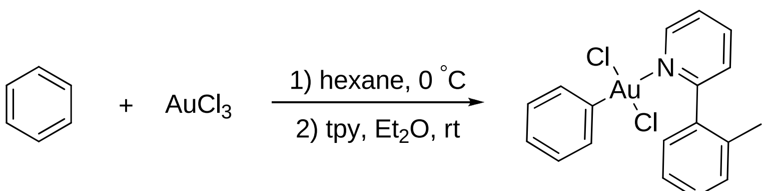

8

Under N_2, a mixture of benzene (1.06 mL, 12 mmol) and hexane (4 mL) was added dropwise to a hexane suspension of gold(III) chloride (181.1 mg, 0.6 mmol) at 0 °C. After being stirring at 0 °C for 1 h, the reaction mixture was added diethyl ether (4 mL), treated with diethyl ether solution (4 mL) of 2-(*o*-tolyl)pyridine (101.4 mg, 0.6 mmol, 1.0 equiv), and continued stirring at room temperature for 1 h. The resulting suspension was filtered through a pad of Celite, and washed with diethyl ether. The combined organic extracts were concentrated and the resulting residue was purified by silica gel chromatography (hexane/CH_2Cl_2 = 1/1, v/v) to provide [AuCl$_2$(Ph)(tpy)] (**8**) as a yellowish solid in 26% yield (80.0 mg). M.p.: 166-168 °C. ^{1}H NMR (CD_2Cl_2, 400 MHz): δ = 2.31 (s, 3H), 7.82-7.84 (m, 2H), 7.01-7.03 (m, 3H), 7.42 (d, J = 7.2 Hz, 1H), 7.47-7.55 (m, 2H), 7.64-7.67 (m, 2H), 7.78-7.79 (m, 1H), 8.02-8.06 (m, 1H), 8.79 (d, J = 5.2 Hz, 1H) ppm. ^{13}C NMR (CD_2Cl_2, 100 MHz): δ = 20.7, 125.2, 126.2, 127.0, 129.0, 129.3 130.3, 130.6, 131.1, 131.4, 132.3, 137.3, 138.0, 139.8, 149.5, 159.6 ppm. Anal. Calcd for $C_{18}H_{16}AuCl_2N$ (%): C, 42.04; H, 3.14; N, 2.72. Found: C, 42.37; H, 3.21; N, 2.67.

(7) **Stoichiometric reaction of [AuCl$_2$(Ph)(tpy)]** (**8**)

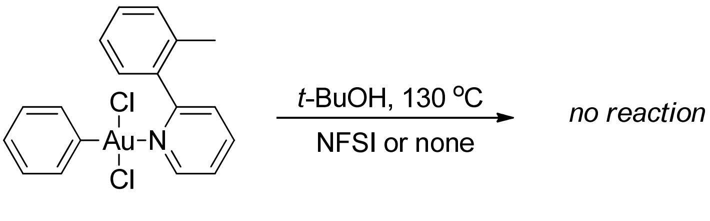

8

A sealed tube with a magnetic stirring bar was charged with [AuCl$_2$(Ph)(tpy)] **8** (22.6 mg, 0.05 mmol) and *t*-BuOH (1 mL) either in the presence or absence of NFSI (47.3 mg, 0.15 mmol) under N_2. A rubber septum was replaced with Teflon stopper, and the

system was evacuated twice and back filled with N_2. Then the reaction mixture was stirred at 130 °C for 12 h. The reaction mixture was then cooled to ambient temperature and diluted with 10 mL of CH_2Cl_2. Then the organic phase was detected by GC-Mass, and no arylated product **4aa** was observed.

III. ESI-HRMS detection[7]

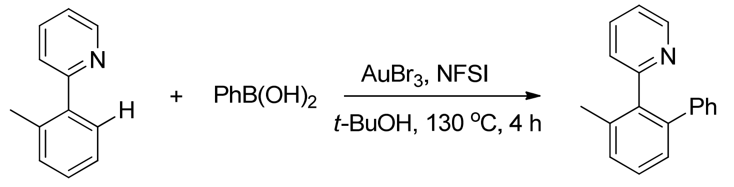

1a **4aa**

A sealed tube with a magnetic stirring bar was charged with $AuBr_3$ (2.2 mg, 0.005 mmol), 2-(*o*-tolyl)pyridine (16.9 mg, 0.1 mmol), phenylboronic acid (36.6 mg, 0.3 mmol), NFSI (94.6 mg, 0.3 mmol) and *t*-BuOH (1.0 mL) under N_2. A rubber septum was replaced with Teflon stopper, and the system was evacuated twice and back filled with N_2. After being stirred at 130 °C for 4 h, the reaction mixture was cooled to ambient temperature, diluted with methanol, and then detected by ESI-HRMS.

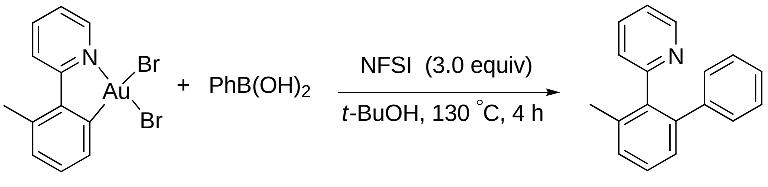

3b **4aa**

A sealed tube with a magnetic stirring bar was charged with **3b** (52.3 mg, 0.10 mmol), phenylboronic acid (36.6 mg, 0.3 mmol), NFSI (94.6 mg, 0.3 mmol) and *t*-BuOH (1.0 mL) under N_2. A rubber septum was replaced with Teflon stopper, and the system was evacuated twice and back filled with N_2. After being stirred at 130 °C for 4 h, the reaction mixture was cooled to ambient temperature, diluted with methanol, and then detected by ESI-HRMS.

IV. Kinetic isotope effect experiments

(1) Synthesis of [D]-1a[8]

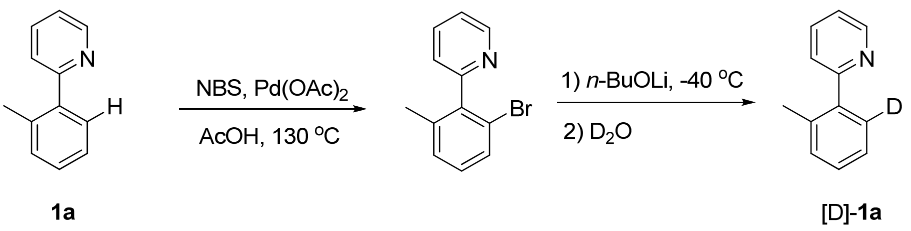

To a solution of 2-(*o*-tolyl)pyridine (933.4 mg, 5.52 mmol) in AcOH (30 mL) in a sealed tube was added *N*-bromosuccinimide (1.16 g, 6.62 mmol) and $Pd(OAc)_2$ (124 mg, 0.55 mmol). The reaction mixture was heated to 120 °C for 24 h. Then the reaction mixture was cooled to room temperature and solvent was removed in vacuum. The residue was purified by silica gel chromatography (EtOAc/hexanes = 1:10, v/v) to afford 2-(2-bromo-6-methylphenyl)pyridine as pale yellow oil in 51% yield (700 mg). ^{1}H NMR ($CDCl_3$, 400 MHz): δ = 2.09 (s, 3H), 7.14 (t, *J* = 7.6 Hz, 1H), 7.21 (d, *J* = 7.6 Hz, 1H), 7.27-7.31 (m, 2H), 7.49 (d, *J* = 8.0 Hz, 1H), 7.78 (td, *J* = 7.6 Hz, *J* = 1.2 Hz, 1H), 8.73 (d, *J* = 4.8 Hz, 1H) ppm. ^{13}C NMR ($CDCl_3$, 100 MHz): δ = 20.8, 122.4, 123.1, 124.8, 129.2, 129.4, 130.2, 136.4, 138.7, 141.2, 149.7, 159.2 ppm. HRMS (ESI$^+$): calcd for $C_{12}H_{11}BrN$ $[M+H]^+$ 248.0075, found 248.0070.

To the solution of 2-(2-bromo-6-methylphenyl)pyridine (123.5 mg, 0.5 mmol) in 8 mL of dry diethyl ether, *n*-butyl lithium (0.4 mL of 2.5 M in hexane, 1.0 mmol, 2 equiv) was added dropwise at -40 ºC under nitrogen. After stirring for 30 min, the reaction mixture was quenched with 0.8 mL of D_2O, and was continued stirring for another hour. The reaction mixture was diluted with 20 mL of ethyl acetate and washed with brine. The organic layer was dried over Na_2SO_4 and concentrated under vacuum. The residue was purified by column chromatography on silica gel (hexane/EtOAc = 10/1, v/v) to give [D]-**1a** as colorless oil in 82% yield (69.7 mg). ^{1}H NMR ($CDCl_3$, 400 MHz): δ = 2.30 (s, 3H), 7.16-7.25 (m, 4H), 7.33 (d, *J* = 8.0 Hz, 1H), 7.67 (td, *J* = 7.6 Hz, *J* = 2.0 Hz, 1H), 8.63 (d, *J* = 4.8 Hz, 1H) ppm. ^{13}C NMR ($CDCl_3$, 100 MHz): δ = 20.4, 121.7, 124.2, 125.9, 128.4, 130.8, 135.9, 136.2, 140.5, 149.4, 160.2 ppm. HRMS (ESI$^+$): calcd for $C_{12}H_{11}DN$ $[M+H]^+$ 171.1033, found 171.1027.

(2) Hydrogen-deuterium exchange experiment

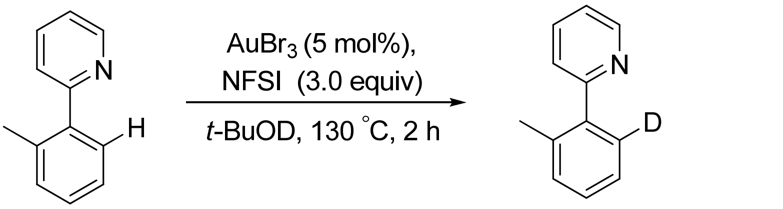

1a

[D]-**1a**: no observed
(both with and without $PhB(OH)_2$)

A sealed tube with a magnetic stirring bar was charged with $AuBr_3$ (2.2 mg, 0.005 mmol), 2-(*o*-tolyl)pyridine (16.6 mg, 0.1 mmol), phenylboronic acid (36.6 mg, 0.3 mmol), NFSI (94.6 mg, 0.3 mmol) and *t*-BuOD (1.0 mL) under N_2. A rubber septum was replaced with Teflon stopper, and the system was evacuated twice and back filled with N_2. The reaction mixture was refluxed at 130 °C in 2 h. The reaction mixture was then cooled to ambient temperature, diluted with 10 mL of CH_2Cl_2, filtered through a pad of Celite, and washed with 10-20 mL of CH_2Cl_2. The combined organic extracts were concentrated and the resulting residue was purified by neutral alumina column chromatography (petroleum ether/acetone = 30:1, v/v) to recover the starting material **1a**. ^1H NMR (acetone-d_6, 400 MHz) analysis showed that the hydrogen at the *ortho*-position of phenyl ring of 2-(*o*-tolyl)pyridine (**1a**) was not deuterated. No [D]-**1a** was observed when the reaction was carried out in the absence of $PhB(OH)_2$ in 2 h.

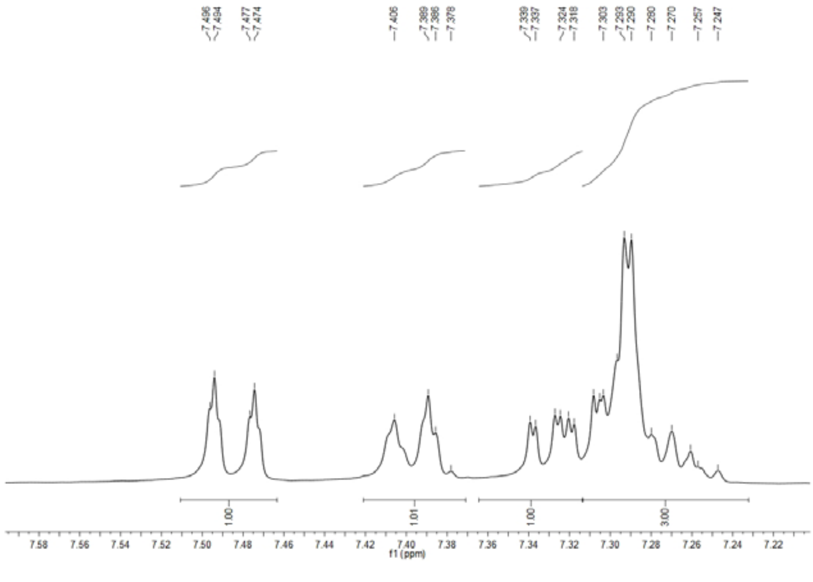

Figure S1. ^1H NMR spectrum of hydrogen-deuterium exchange experiment.

(3) Kinetic isotope effect[9]

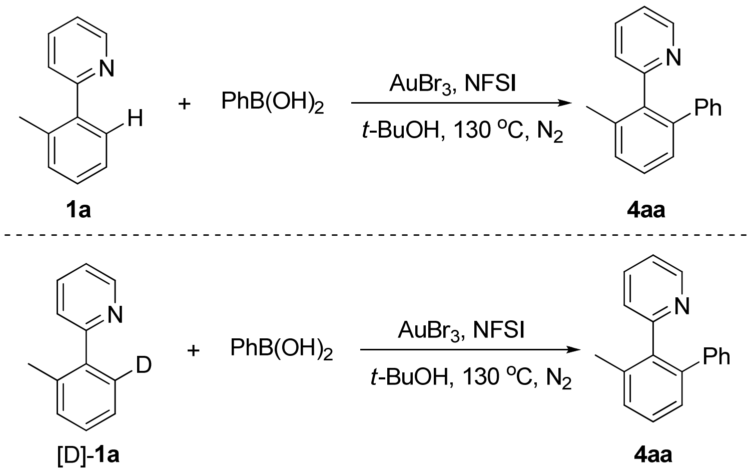

A sealed tube with a magnetic stirring bar was charged with **1a** or [D]-**1a** (0.1 mmol), phenylboronic acid (36.6 mg, 0.3 mmol), gold(III) bromide (2.2 mg, 0.005 mmol), NFSI (94.6 mg, 0.3 mmol), and *t*-BuOH (1 mL) under N_2. A rubber septum was replaced with Teflon stopper, and the system was evacuated twice and back filled with N_2. The resulting mixture was stirred at 130 °C for designated time (20 min, 40 min, 60 min, 80 min, and 120 min) and then an internal standard (1,1,2,2-tetrachloroethane, 10.5 μL, 0.1 mmol) were added. The solution was filtered through a pad of Celite and washed with 10 mL of dichloromethane. The filtrate were concentrated and subjected to ^1H NMR analysis to determine the concentration of product. KIE = 0.001371/0.001414 = 0.96.

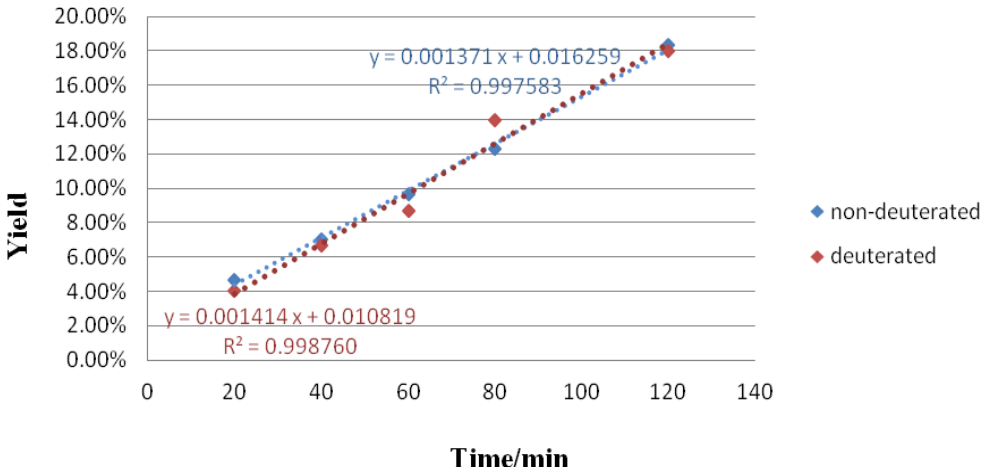

Figure S2. Plots of yield versus reaction time.

V. ORTEP diagrams of 3b, 7 and 8

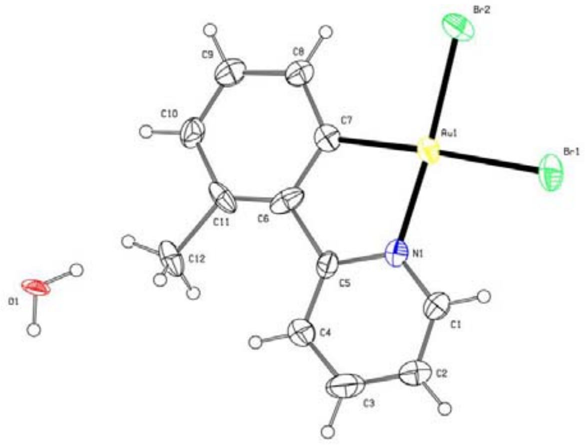

Figure S3. ORTEP drawing of **3b** with 50% probability thermal ellipsoids.

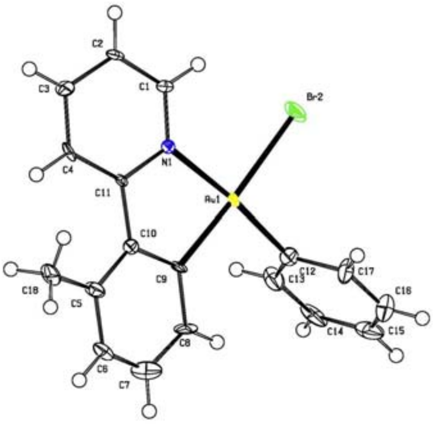

Figure S4. ORTEP drawing of **7** with 50% probability thermal ellipsoids.

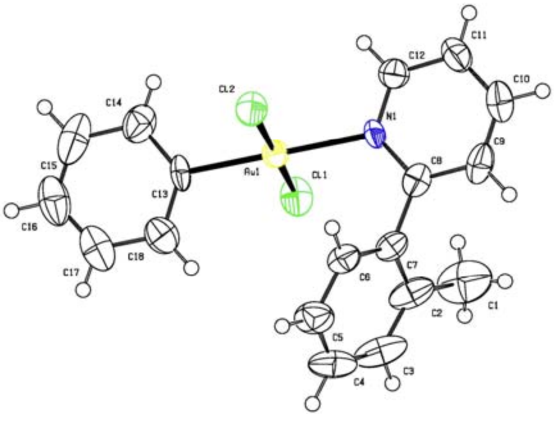

Figure S5. ORTEP drawing of **8** with 50% probability thermal ellipsoids.

VI. General procedure for gold-catalyzed directed C(sp^2)–H bond arylation with arylboronic acid

A sealed tube with a magnetic stirring bar was charged with $AuBr_3$ (4.4 mg, 0.01 mmol), *N*-heteroarene-containing arene (0.2 mmol), arylboronic acid (0.6 mmol, 3.0 equiv), NFSI (189.2 mg, 0.6 mmol) and *t*-BuOH (2.0 mL) under N_2. A rubber septum was replaced with Teflon stopper, and the system was evacuated twice and back filled with N_2. Then the reaction mixture was stirred at the indicated temperature for 24 h. The reaction mixture was then cooled to ambient temperature, diluted with 10 mL of CH_2Cl_2, filtered through a Celite pad, and washed with 10-20 mL of CH_2Cl_2. The combined organic extracts were concentrated and the resulting residue was purified by neutral alumina column chromatography to provide the desired product.

VII. Experimental data for the described substances

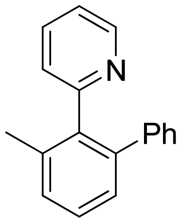

2-(3-Methylbiphenyl-2-yl)pyridine (4aa)

2-(*o*-Tolyl)pyridine (33.8 mg, 0.2 mmol), phenylboronic acid (73.2 mg, 0.6 mmol), $AuBr_3$ (4.4 mg, 0.01 mmol), NFSI (189.2 mg, 0.6 mmol) and *t*-BuOH (2.0 mL) at 130 °C for 24 h. Purification via neutral alumina column chromatography (petroleum ether/acetone = 30:1, v/v) afforded a white solid in 91% yield (44.6 mg). M.p.: 49-50 °C. ^{1}H NMR ($CDCl_3$, 400 MHz): δ = 2.20 (s, 3H), 6.89 (d, J = 7.6 Hz, 1H), 7.07-7.10 (m, 3H), 7.11-7.17 (m, 3H), 7.29 (t, J = 7.6 Hz, 2H), 7.37 (t, J = 7.6 Hz, 1H), 7.44 (td, J = 7.6 Hz, J = 1.6 Hz, 1H), 8.63 (d, J = 4.8 Hz, 1H) ppm. ^{13}C NMR ($CDCl_3$, 100 MHz): δ = 20.6, 121.4, 125.7, 126.3, 127.68, 127.69, 128.1, 129.5, 129.7, 135.8, 136.8, 139.4, 141.4, 141.8, 148.9, 159.7 ppm. HRMS (ESI$^+$): calcd for

$C_{18}H_{16}N$ $[M+H]^+$ 246.1283, found 246.1284. *Characterization data were consistent with literature values.*[10]

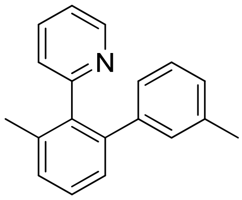

2-(3'-Methyl-3-methylbiphenyl-2-yl)pyridine (4ac)

2-(*o*-Tolyl)pyridine (33.8 mg, 0.2 mmol), 3-methylphenylboronic acid (81.6 mg, 0.6 mmol), $AuBr_3$ (4.4 mg, 0.01 mmol), NFSI (189.2 mg, 0.6 mmol) and *t*-BuOH (2.0 mL) at 130 °C for 24 h. Purification via neutral alumina column chromatography (petroleum ether/acetone = 30:1, v/v) afforded colorless oil in 70% yield (36.3 mg). ^{1}H NMR ($CDCl_3$, 400 MHz): δ = 2.19 (s, 3H), 2.20 (s, 3H), 6.86 (d, J = 7.6 Hz, 1H), 6.90 (d, J = 7.6 Hz, 1H), 6.92-6.94 (m, 2H), 7.01 (t, J = 8.0 Hz, 1H), 7.07-7.11 (m, 1H), 7.27-7.30 (m, 2H), 7.36 (t, J = 7.2 Hz, 1H), 7.45 (td, J = 7.6 Hz, J = 1.6 Hz, 1H), 8.64 (d, J = 4.0 Hz, 1H) ppm. ^{13}C NMR ($CDCl_3$, 100 MHz): δ = 20.6, 21.4, 121.3, 125.7, 126.8, 127.0, 127.5, 127.7, 128.1, 129.4, 130.6, 135.8, 136.7, 137.2, 139.4, 141.4, 141.6, 148.9, 159.8 ppm. HRMS (ESI$^+$): calcd for $C_{19}H_{18}N$ $[M+H]^+$ 260.1439, found 260.1438. *Characterization data were consistent with literature values.*[10]

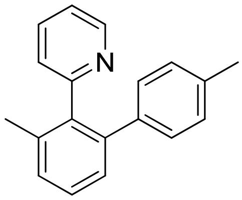

2-(3,4'-Dimethylbiphenyl-2-yl)pyridine (4ad)

2-(*o*-Tolyl)pyridine (33.8 mg, 0.2 mmol), 4-methylphenylboric acid (81.6 mg, 0.6 mmol), $AuBr_3$ (4.4 mg, 0.01 mmol), NFSI (189.2 mg, 0.6 mmol) and *t*-BuOH (2.0 mL) at 130 °C for 24 h. Purification via neutral alumina column chromatography (petroleum ether/acetone = 30:1, v/v) afforded colorless oil 80% yield (41.5 mg). ^{1}H NMR ($CDCl_3$, 400 MHz): δ = 2.20 (s, 3H), 2.28 (s, 3H), 6.91 (d, J = 7.6 Hz, 1H), 6.95-7.02 (m, 4H), 7.11-7.14 (m, 1H), 7.28-7.31 (m, 2H), 7.37 (t, J = 7.6 Hz, 1H),

7.48 (td, J = 8.0 Hz, J = 2.0 Hz, 1H), 8.66 (d, J = 4.8 Hz, 1H) ppm. ^{13}C NMR ($CDCl_3$, 100 MHz): δ = 20.6, 21.2, 121.4, 125.7, 127.8, 128.1, 128.5, 129.3, 129.6, 135.86, 135.93, 136.8, 138.8, 139.4, 141.3, 149.0, 159.9 ppm. HRMS (ESI$^+$): calcd for $C_{19}H_{18}N$ [M+H]$^+$ 260.1439, found 260.1440. *Characterization data were consistent with literature values.*[10]

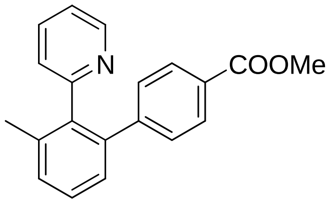

Methyl 3'-methyl-2'-(pyridin-2-yl)-[1,1'-biphenyl]-4-carboxylate (4ae)

2-(*o*-Tolyl)pyridine (33.0 mg, 0.2 mmol), 4-methoxycarbonylphenylboronic acid (108.0 mg, 0.6 mmol), $AuBr_3$ (4.4 mg, 0.01 mmol), NFSI (189.2 mg, 0.6 mmol) and *t*-BuOH (2.0 mL) at 130 °C for 24 h. Purification via neutral alumina column chromatography (petroleum ether/acetone = 20:1, v/v) afforded a white solid in 61% yield (37.0 mg). M.p.: 92-93 °C. ^{1}H NMR ($CDCl_3$, 400 MHz): δ = 2.18 (s, 3H), 3.86 (s, 3H), 6.88 (d, J = 7.6 Hz, 1H), 7.08-7.11 (m, 1H)，7.12-7.15 (m, 2H), 7.25-7.27 (m, 1H), 7.32 (d, J = 6.8 Hz, 1H), 7.37 (t, J = 7.6 Hz, 1H), 7.44 (td, J = 7.6 Hz, J = 2.0 Hz, 1H), 7.79-7.82 (m, 2H), 8.60-8.62 (m, 1H) ppm. ^{13}C NMR ($CDCl_3$, 100 MHz): δ = 20.6, 52.2, 121.7, 125.7, 127.5, 128.0, 128.3, 129.0, 129.7, 130.2, 136.0, 137.0, 139.4, 140.3, 146.7, 149.1, 159.2, 167.2 ppm. HRMS (ESI$^+$): calcd for $C_{20}H_{18}NO_2$ [M+H]$^+$ 304.1338, found 304.1343. Anal. Calcd for $C_{20}H_{17}NO_2$ (%): C, 79.19; H, 5.65; N, 4.62, found: C, 78.81; H, 5.71; N, 4.48.

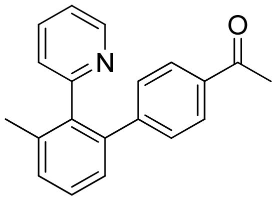

1-(3'-Methyl-2'-(pyridin-2-yl)-[1,1'-biphenyl]-4-yl)ethanone (4af)

2-(*o*-Tolyl)pyridine (33.8 mg, 0.2 mmol), 4-acetylphenylboronic acid (98.4 mg, 0.6 mmol), $AuBr_3$ (4.4 mg, 0.01 mmol), NFSI (189.2 mg, 0.6 mmol) and *t*-BuOH (2.0 mL)

at 130 °C for 24 h. Purification via neutral alumina column chromatography (petroleum ether/acetone = 20:1, v/v) afforded a white solid in 62% yield (35.6 mg). M.p.: 84-86 °C. ^{1}H NMR ($CDCl_3$, 400 MHz): δ = 2.18 (s, 3H), 2.53 (s, 3H), 6.89 (d, J = 7.6 Hz, 1H), 7.09-7.12 (m, 1H), 7.16 (d, J = 8.4 Hz, 2H), 7.26 (d, J = 7.6 Hz, 1H), 7.33 (d, J = 6.8 Hz, 1H), 7.38 (t, J = 7.6 Hz, 1H), 7.46 (td, J = 7.6 Hz, J = 1.6 Hz, 1H), 7.73 (d, J = 8.0 Hz, 2H), 8.62 (d, J = 4.8 Hz, 1H) ppm. ^{13}C NMR ($CDCl_3$, 100 MHz): δ = 20.6, 26.7, 121.7, 125.7, 127.5, 127.9, 128.3, 129.9, 130.2, 135.1, 136.0, 137.1, 139.4, 140.2, 146.9, 149.2, 159.2, 198.0 ppm. HRMS (ESI$^+$): calcd for $C_{20}H_{18}NO$ $[M+H]^+$ 288.1388, found 288.1386. *Characterization data were consistent with literature values.*[10]

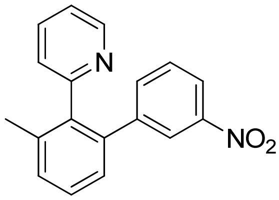

2-(3'-Nitro-3-methylbiphenyl-2-yl)pyridine (4ag)

2-(*o*-Tolyl)pyridine (33.8 mg, 0.2 mmol), 3-nitrophenylboronic acid (100.2 mg, 0.6 mmol), $AuBr_3$ (4.4 mg, 0.01 mmol), NFSI (189.2 mg, 0.6 mmol) and *t*-BuOH (2.0 mL) at 130 °C for 24 h. Purification via neutral alumina column chromatography (petroleum ether/acetone = 20:1, v/v) afforded yellow oil in 35% yield (20.3 mg). ^{1}H NMR ($CDCl_3$, 400 MHz): δ = 2.20 (s, 3H), 6.95 (d, J = 7.6 Hz, 1H), 7.10-7.14 (m, 1H), 7.26-7.29 (m, 2H), 7.35-7.43 (m, 3H), 7.51 (td, J = 7.6 Hz, J = 2.0 Hz, 1H), 7.96-8.00 (m, 2H), 8.62 (d, J = 4.8 Hz, 1H) ppm. ^{13}C NMR ($CDCl_3$, 100 MHz): δ = 20.5, 121.4, 121.9, 124.5, 125.6, 127.5, 128.5, 128.6, 130.6, 135.8, 136.2, 137.2, 138.9, 139.6, 143.4, 147.8, 149.4, 158.8 ppm. HRMS (ESI$^+$): calcd for $C_{18}H_{15}N_2O_2$ $[M+H]^+$ 291.1134, found 291.1139. *Characterization data were consistent with literature values.*[10]

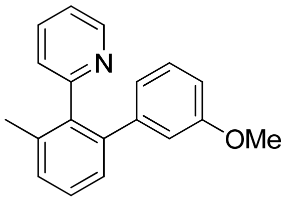

2-(3'-Methoxyl-3-methylbiphenyl-2-yl)pyridine (4ah)

2-(*o*-Tolyl)pyridine (33.8 mg, 0.2 mmol), 3-methoxylphenylboronic acid (91.2 mg, 0.6 mmol), $AuBr_3$ (4.4 mg, 0.01 mmol), NFSI (189.2 mg, 0.6 mmol) and *t*-BuOH (2.0 mL) at 130 °C for 24 h. Purification via neutral alumina column chromatography (petroleum ether/acetone = 20:1, v/v) afforded yellowish oil in 44% yield (24.2 mg). ^{1}H NMR ($CDCl_3$, 400 MHz): δ = 2.19 (s, 3H), 3.59 (s, 3H), 6.58-6.59 (m, 1H), 6.66-6.69 (m, 1H), 6.70-6.73 (m, 1H), 6.89-6.91 (m, 1H), 7.07 (t, J = 8.0 Hz, 1H), 7.09-7.13 (m, 1H), 7.28-7.31 (m, 2H). 7.36 (t, J = 7.6 Hz, 1H), 7.47 (td, J = 7.6 Hz, J = 1.6 Hz, 1H), 8.64-8.66 (m, 1H) ppm. ^{13}C NMR ($CDCl_3$, 100 MHz): δ = 20.6, 55.2, 112.9, 114.7, 121.5, 122.2, 125.7, 127.6, 128.2, 128.8, 129.6, 136.0, 136.9, 139.3, 141.2, 143.1, 148.9, 158.8, 159.8 ppm. calcd for $C_{19}H_{18}NO$ $[M+H]^+$ 276.1388, found 276.1385. Anal. Calcd for $C_{19}H_{17}NO$ (%): C, 82.88; H, 6.22; N, 5.09, found: C, 82.41; H, 6.08; N, 4.77.

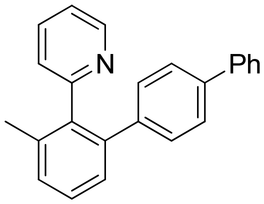

2-(3-Methyl-[1,1':4',1''-terphenyl]-2-yl)pyridine (4ai)

2-(*o*-Tolyl)pyridine (33.8 mg, 0.2 mmol), 4-biphenylboric acid (118.9 mg, 0.6 mmol), $AuBr_3$ (4.4 mg, 0.01 mmol), NFSI (189.2 mg, 0.6 mmol) and *t*-BuOH (2.0 mL) at 130 °C for 24 h. Purification via neutral alumina column chromatography (petroleum ether/acetone = 30:1, v/v) afforded a white solid in 83% yield (53.3 mg). M.p.: 109-111 °C. ^{1}H NMR ($CDCl_3$, 400 MHz): δ = 2.21 (s, 3H), 6.94 (d, J = 8.0 Hz, 1H) 7.09-7.12 (m, 1H), 7.14-7.17 (m, 2H), 7.31-7.34 (m, 3H), 7.37-7.42 (m, 5H), 7,47 (td, J = 7.6 Hz, J = 2.0 Hz, 1H), 7.54-7.56 (m, 2H), 8.65-7.67 (m, 1H) ppm. ^{13}C NMR

(CDCl$_3$, 100 MHz): δ = 20.6, 121.5, 125.8, 126.4, 127.0, 127.3, 127.7, 128.2, 128.8, 129.6, 130.2, 135.9, 136.9, 139.0, 139.5, 140.76, 140.82, 140.9, 149.0, 159.7 ppm. HRMS (ESI$^+$): calcd for $C_{24}H_{20}N$ $[M+H]^+$ 322.1596, found 322.1595. Anal. Calcd for $C_{24}H_{19}N$ (%): C, 89.68; H, 5.96; N, 4.36, found: C, 89.58; H, 6.20; N, 4.17.

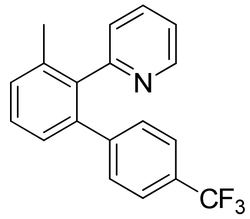

2-(4'-Trifluoromethyl-3-methylbiphenyl-2-yl)pyridine (4aj)

2-(*o*-Tolyl)pyridine (33.8 mg, 0.2 mmol), 4-trifluoromethylphenylboronic acid (114.0 mg, 0.6 mmol), $AuBr_3$ (4.4 mg, 0.01 mmol), NFSI (189.2 mg, 0.6 mmol) and *t*-BuOH (2.0 mL) at 130 °C for 24 h. Purification via neutral alumina column chromatography (petroleum ether/acetone = 30:1, v/v) afforded a white solid in 64% yield (40.1 mg). M.p.: 46-47 °C. ^{1}H NMR (CDCl$_3$, 400 MHz): δ = 2.19 (s, 3H), 6.89 (d, J = 7.6 Hz, 1H), 7.10-7.14 (m, 1H), 7.19 (d, J = 8.0 Hz, 2H), 7.25 (d, J = 6.8 Hz, 1H), 7.34 (d, J = 7.2 Hz, 1H), 7.37-7.40 (m, 3H), 7.48 (td, J = 8.0 Hz, J = 1.6 Hz, 1H), 8.62 (d, J = 4.4 Hz, 1H) ppm. ^{13}C NMR (CDCl$_3$, 100 MHz): δ = 20.5, 121.8, 124.62, 124.65, 124.69, 124.73, 125.7, 127.6, 128.4, 130.0, 130.3, 136.0, 137.1, 139.5, 140.0, 145.5, 149.2, 159.2 ppm. HRMS (ESI$^+$): calcd for $C_{19}H_{15}F_3N$ $[M+H]^+$ 314.1157, found 314.1153. *Characterization data were consistent with literature values.*[10]

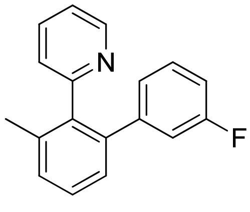

2-(3'-Fluoro-3-methylbiphenyl-2-yl)pyridine (4ak)

2-(*o*-Tolyl)pyridine (33.8 mg, 0.2 mmol), 3-fluorophenylboronic acid (84.0 mg, 0.6 mmol), $AuBr_3$ (4.4 mg, 0.01 mmol), NFSI (189.2 mg, 0.6 mmol) and *t*-BuOH (2.0 mL) at 130 °C for 24 h. Purification via neutral alumina column chromatography

(petroleum ether/acetone = 30:1, v/v) afforded colorless oil in 73% yield (38.4 mg). ^{1}H NMR ($CDCl_3$, 400 MHz): δ = 2.20 (s, 3H), 6.81-6.86 (m , 3H), 6.93 (d, J = 8.0 Hz, 1H), 7.07-7.15 (m, 2H), 7.28 (s, 1H), 7.33 (d, J = 6.8 Hz, 1H), 7.38 (t, J = 7.6 Hz, 1H), 7.50 (td, J = 7.6 Hz, J = 1.6 Hz, 1H), 8.65 (d, J = 5.6 Hz, 1H) ppm. ^{13}C NMR ($CDCl_3$, 100 MHz): δ = 20.6, 113.3 (d, J = 20.9 Hz), 116.6 (d, J = 21.6 Hz), 121.6, 125.57 (d, J = 2.8 Hz), 125.61, 127.6, 127.5, 128.2, 129.1 (d, J = 8.3 Hz), 130.0, 136.0, 137.0, 140.2, 144.1 (d, J = 7.8 Hz), 149.1, 159.3, 162.3 (d, J = 243.7 Hz) ppm. HRMS (ESI$^+$): calcd for $C_{18}H_{15}FN$ $[M+H]^+$ 264.1189, found 264.1185. Anal. Calcd for $C_{18}H_{14}FN$ (%): C, 82.11; H, 5.36; N, 5.32, found: C, 81.81; H, 5.42; N, 5.40.

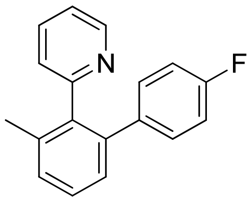

2-(4'-Fluoro-3-methylbiphenyl-2-yl)pyridine (4al)

2-(*o*-Tolyl)pyridine (33.8 mg, 0.2 mmol), 4-fluorophenylboronic acid (84.1 mg, 0.6 mmol), $AuBr_3$ (4.4 mg, 0.01 mmol), NFSI (189.2 mg, 0.6 mmol) and *t*-BuOH (2.0 mL) at 130 °C for 24 h. Purification via neutral alumina column chromatography (petroleum ether/acetone = 30:1, v/v) afforded a white solid in 80% yield (42.1 mg). M.p.: 68-70 °C. ^{1}H NMR ($CDCl_3$, 400 MHz): δ = 2.18 (s, 3H), 6.82 (t, J = 8.4 Hz, 2H), 6.88 (d, J = 7.6 Hz, 1H), 7.01-7.05 (m, 2H), 7.09-7.12 (m, 1H), 7.23 (d, J = 7.6 Hz, 1H), 7.29 (d, J = 7.6 Hz, 1H), 7.35 (t, J = 7.6 Hz, 1H), 7.47 (t, J = 7.6 Hz, 1H), 8.63 (d, J = 4.8 Hz, 1H) ppm. ^{13}C NMR ($CDCl_3$, 100 MHz): δ = 20.6, 114.6 (d, J = 21.1 Hz), 121.5, 125.7, 127.6, 128.2, 129.6, 131.2 (d, J = 7.6 Hz), 136.0, 136.9, 137.7 (d, J = 3.3 Hz), 139.5, 140.3, 149.1, 159.5, 160.6 (d, J = 244.0 Hz) ppm. HRMS (ESI$^+$): calcd for $C_{18}H_{15}FN$ $[M+H]^+$ 264.1189, found 264.1185. *Characterization data were consistent with literature values.*[10]

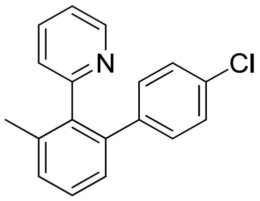

2-(4'-Chloro-3-methylbiphenyl-2-yl)pyridine (4am)

2-(*o*-Tolyl)pyridine (33.8 mg, 0.2 mmol), 4-chlorophenylboronic acid (93.6 mg, 0.6 mmol), $AuBr_3$ (4.4 mg, 0.01 mmol), NFSI (189.2 mg, 0.6 mmol) and *t*-BuOH (2.0 mL) at 140 °C for 24 h. Purification via neutral alumina column chromatography (petroleum ether/acetone = 30:1, v/v) afforded a yellowish solid in 76% yield (42.4 mg). M.p.: 54-56 °C. ^{1}H NMR ($CDCl_3$, 400 MHz): δ = 2.17 (s, 3H), 6.89 (d, J = 8.0 Hz, 1H), 7.00 (d, J = 8.4 Hz, 2H), 7.09-7.13 (m, 3H), 7.23 (d, J = 7.6 Hz, 1H), 7.30 (d, J = 7.2 Hz, 1H), 7.36 (t, J = 7.6 Hz, 1H), 7.49 (td, J = 8.0 Hz, J = 2.0 Hz, 1H), 8.63 (d, J = 4.8 Hz, 1H) ppm. ^{13}C NMR ($CDCl_3$, 100 MHz): δ = 20.6, 121.6, 125.6, 127.6, 127.9, 128.3, 129.8, 131.0, 132.5, 136.0, 137.0, 139.4, 140.1, 140.3, 149.1, 159.4 ppm. HRMS (ESI$^+$): calcd for $C_{18}H_{15}ClN$ $[M+H]^+$ 280.0893, found 280.0891. *Characterization data were consistent with literature values.*[10]

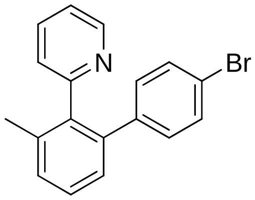

2-(4'-Bromo-3-methylbiphenyl-2-yl)pyridine (4an)

2-(*o*-Tolyl)pyridine (33.0 mg, 0.2 mmol), 4-bromophenylboronic acid (120.0 mg, 0.6 mmol), $AuBr_3$ (4.4 mg, 0.01 mmol), NFSI (189.2 mg, 0.6 mmol) and *t*-BuOH (2.0 mL) at 140 °C for 24 h. Purification via neutral alumina column chromatography (petroleum ether/acetone = 30:1, v/v) afforded a white solid in 67% yield (43.3 mg). M.p.: 68-70 °C. ^{1}H NMR ($CDCl_3$, 400 MHz): δ = 2.20 (s, 3H), 6.91 (d, J = 7.6 Hz, 1H), 6.97 (d, J = 8.4 Hz, 2H), 7.13-7.16 (m, 1H), 7.24-7.29 (m, 3H), 7.33 (d, J = 6.8 Hz, 1H), 7.38 (t, J = 7.6 Hz, 1H), 7.51 (td, J = 7.6 Hz, J = 1.6 Hz, 1H), 8.65 (d, J = 4.4 Hz, 1H) ppm. ^{13}C NMR ($CDCl_3$, 100 MHz): δ = 20.6, 120.7, 121.6, 125.6, 127.5,

128.3, 129.9, 130.9, 131.4, 136.0, 137.0, 139.4, 140.1, 140.7, 149.1, 159.4 ppm. HRMS (ESI^+): calcd for $C_{18}H_{15}BrN$ $[M+H]^+$ 324.0388, found 324.0388. *Characterization data were consistent with literature values.*[10]

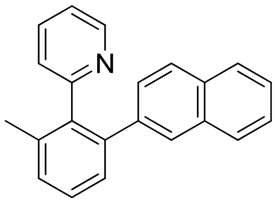

2-(2-Methyl-6-(naphthalen-2-yl)phenyl)pyridine (4ao)

2-(*o*-Tolyl)pyridine (33.8 mg, 0.2 mmol), 2-naphthaleneboronic acid (103.2 mg, 0.6 mmol), $AuBr_3$ (4.4 mg, 0.01 mmol), NFSI (189.2 mg, 0.6 mmol) and *t*-BuOH (2.0 mL) at 130 °C for 24 h. Purification via neutral alumina column chromatography (petroleum ether/acetone = 30:1, v/v) afforded colorless oil in 72% yield (42.5 mg). ^1H NMR ($CDCl_3$, 400 MHz): δ = 2.22 (s, 3H), 6.91 (d, J = 8.0 Hz, 1H), 7.04-7.07 (m, 1H), 7.15 (dd, J = 8.4 Hz, J = 1.6 Hz, 1H), 7.33-7.42 (m, 6H), 7.56 (d, J = 8.4 Hz,1H), 7.64 (s, 1H), 7.68-7.74 (m, 2H), 8.63-8.65 (m, 1H) ppm. ^{13}C NMR ($CDCl_3$, 100 MHz): δ = 20.6, 121.4, 125.77, 125.81, 126.0, 127.0, 127.6, 128.05, 128.08, 128.1, 128.2, 128.6, 129.7, 131.9, 133.2, 135.9, 137.0, 139.4, 139.6, 141.2, 149.0, 159.6 ppm. HRMS (ESI^+): calcd for $C_{22}H_{18}N$ $[M+H]^+$ 296.1439, found 296.1440. *Characterization data were consistent with literature values.*[11]

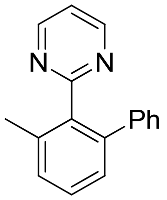

2-(3-Methylbiphenyl-2-yl)pyrimidine (4ba)

2-(*o*-Tolyl)pyrimidine (34.0 mg, 0.2 mmol), phenylboronic acid (73.2 mg, 0.6 mmol), $AuBr_3$ (4.4 mg, 0.01 mmol), NFSI (189.2 mg, 0.6 mmol) and *t*-BuOH (2.0 mL) at 130 °C for 24 h. Purification via neutral alumina column chromatography (petroleum ether/acetone = 20:1, v/v) afforded a white solid in 41% yield (20.2 mg). M.p.: 97-98 °C. ^1H NMR ($CDCl_3$, 400 MHz): δ = 2.21 (s, 3H), 7.07-7.15 (m, 6H), 7.29 (d, J

= 7.6 Hz, 2H), 7.39 (t, *J* = 7.6 Hz, 1H), 8.66 (d, *J* = 4.8 Hz, 2H) ppm. ^{13}C NMR ($CDCl_3$, 100 MHz): δ = 20.2, 118.6, 126.4, 127.8, 127.9, 128.7, 129.2, 129.5, 136.1, 138.4, 141.2, 141.7, 156.5, 168.4 ppm. HRMS (ESI$^+$): calcd for $C_{17}H_{15}N_2$ [M+H]$^+$ 247.1235, found 247.1236. Anal. Calcd for $C_{17}H_{14}N_2$ (%): C, 82.90; H, 5.73; N, 11.37, found: C, 83.02; H, 5.99; N, 11.23.

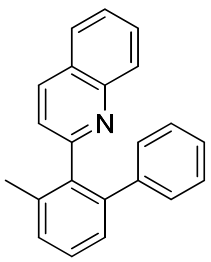

2-(3-Methyl-[1,1'-biphenyl]-2-yl)quinoline (4ca)

2-(*o*-Tolyl)quinoline (43.8 mg, 0.2 mmol), phenylboronic acid (73.2 mg, 0.6 mmol), $AuBr_3$ (4.4 mg, 0.01 mmol), NFSI (189.2 mg, 0.6 mmol) and *t*-BuOH (2.0 mL) at 140 °C for 24 h. Purification via neutral alumina column chromatography (petroleum ether/acetone = 30:1, v/v) afforded yellow oil in 81% yield (47.8 mg). ^{1}H NMR ($CDCl_3$, 400 MHz): δ = 2.23 (s, 3H), 6.98 (d, *J* = 8.4 Hz, 1H), 7.06-7.08 (m, 3H), 7.12-7.14 (m, 2H), 7.33 (t, *J* = 7.6 Hz, 2H), 7.41 (t, *J* = 7.6 Hz, 1H), 7.53 (t, *J* = 7.2 Hz, 1H), 7.70-7.76 (m, 2H), 7.89 (d, *J* = 8.4 Hz, 1H), 8.15 (d, *J* = 8.4 Hz, 1H) ppm. ^{13}C NMR ($CDCl_3$, 100 MHz): δ = 20.7, 123.9, 126.48, 126.54, 127.7, 127.8, 127.9, 128.4, 129.5, 129.9, 135.6, 137.0, 139.5, 141.3, 141.6, 147.7, 160.2 ppm. HRMS (ESI$^+$): calcd for $C_{22}H_{18}N$ [M+H]$^+$ 296.1439, found 260.1438. Anal. Calcd for $C_{22}H_{17}N$ (%): C, 89.46; H, 5.80; N, 4.74, found: C, 89.17; H, 6.14; N, 4.33.

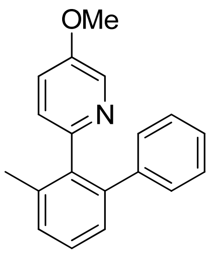

5-Methoxy-2-(3-methyl-[1,1'-biphenyl]-2-yl)pyridine (4da)

5-Methoxy-2-(*o*-tolyl)pyridine (39.8 mg, 0.2 mmol), phenylboronic acid (73.2 mg, 0.6 mmol), $AuBr_3$ (4.4 mg, 0.01 mmol), NFSI (189.2 mg, 0.6 mmol) and *t*-BuOH (2.0 mL) at 130 °C for 24 h. Purification via neutral alumina column chromatography (hexane/acetone = 30:1, v/v) afforded colorless oil in 95% yield (52.3 mg). ^{1}H NMR ($CDCl_3$, 400 MHz): δ = 2.22 (s, 3H), 3.85 (s, 3H), 6.82 (d, J = 8.4 Hz, 1H), 7.00 (dd, J = 8.4 Hz, J = 2.8 Hz, 1H), 7.10-7.12 (m, 2H), 7.15-7.18 (m, 3H), 7.30 (t, J = 7.2 Hz, 2H), 7.37 (t, J = 7.6 Hz, 1H), 8.36 (d, J = 2.8 Hz, 1H) ppm. ^{13}C NMR ($CDCl_3$, 100 MHz): δ = 20.7, 55.6, 120.5, 125.9, 126.2, 127.70, 127.72, 128.0, 129.5, 129.7, 136.2, 137.3, 139.0, 141.6, 142.0, 151.8, 153.8 ppm. HRMS (ESI$^+$): calcd for $C_{19}H_{18}NO$ $[M+H]^+$ 276.1388, found 276.1390. Anal. Calcd for $C_{19}H_{17}NO$ (%): C, 82.88; H, 6.22; N, 5.09, found: C, 82.73; H, 6.12; N, 4.81.

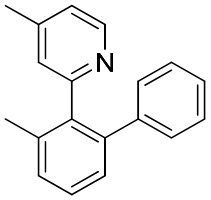

4-Methyl-2-(3-methyl-[1,1'-biphenyl]-2-yl)pyridine (4ea)

4-Methyl-2-(*o*-tolyl)pyridine (36.6 mg, 0.2 mmol), phenylboronic acid (73.2 mg, 0.6 mmol), $AuBr_3$ (4.4 mg, 0.01 mmol), NFSI (189.2 mg, 0.6 mmol) and *t*-BuOH (2.0 mL) at 130 °C for 24 h. Purification via neutral alumina column chromatography (hexane/acetone = 30:1, v/v) afforded colorless oil in 91% yield (47.2 mg). ^{1}H NMR ($CDCl_3$, 400 MHz): δ = 2.17 (s, 3H), 2.21 (s, 3H), 6.74 (s, 1H), 6.92 (dd, J = 4.8 Hz, J = 0.8 Hz, 1H), 7.10-7.17 (m, 5H), 7.28-7.31 (m, 2H), 7.37 (t, J = 7.6 Hz, 1H), 8.49 (d, J = 4.0 Hz, 1H) ppm. ^{13}C NMR ($CDCl_3$, 100 MHz): δ = 20.6, 21.0, 122.4, 126.3, 126.6, 127.62, 127.65, 128.0, 129.4, 129.7, 136.8, 139.6, 141.4, 141.9, 146.8, 148.7, 159.4 ppm. HRMS (ESI$^+$): calcd for $C_{19}H_{18}N$ $[M+H]^+$ 260.1439, found 260.1434.

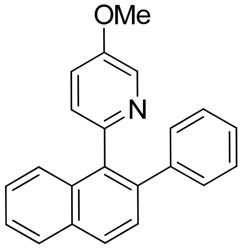

5-Methoxy-2-(2-phenylnaphthalen-1-yl)pyridine (4fa)

5-Methoxy-2-(naphthalen-1-yl)pyridine (47.0 mg, 0.2 mmol), phenylboronic acid (73.2 mg, 0.6 mmol), $AuBr_3$ (4.4 mg, 0.01 mmol), NFSI (189.2 mg, 0.6 mmol) and *t*-BuOH (2.0 mL) at 140 °C for 24 h. Purification via neutral alumina column chromatography (petroleum ether/acetone = 30:1, v/v) afforded a yellowish solid in 45% yield (28.0 mg). M.p.: 134-136 °C. ^{1}H NMR ($CDCl_3$, 400 MHz): δ = 3.76 (s, 3H), 6.65 (dd, J = 8.4 Hz, J = 2.8 Hz, 1H), 6.74 (d, J = 8.4 Hz, 1H), 6.88-7.06 (m, 5H), 7.45 (d, J = 7.2 Hz, 1H), 7.54-7.62 (m, 3H), 7.94-8.02 (m, 3H) ppm. ^{13}C NMR ($CDCl_3$, 100 MHz): δ = 55.8, 119.8, 125.3, 125.6, 126.0, 127.4, 128.7, 129.3, 129.4, 130.8, 131.0, 135.5, 135.9, 138.8, 140.1, 143.6, 152.9, 153.4 ppm. HRMS (ESI$^+$): calcd for $C_{22}H_{18}NO$ $[M+H]^+$ 312.1388, found 312.1382. Anal. Calcd for $C_{22}H_{17}NO$ (%): C, 84.86; H, 5.50; N, 4.50, found: C, 84.73; H, 5.63; N, 4.53.

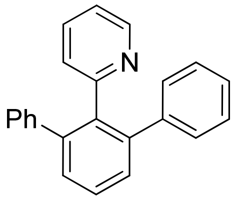

2-([1,1':3',1''-Terphenyl]-2'-yl)pyridine (4ga)

2-((1,1'-Biphenyl)-2-yl)pyridine (46.2 mg, 0.2 mmol), phenylboronic acid (73.2 mg, 0.6 mmol), $AuBr_3$ (4.4 mg, 0.01 mmol), NFSI (189.2 mg, 0.6 mmol) and *t*-BuOH (2.0 mL) at 140 °C for 24 h. Purification via neutral alumina column chromatography (petroleum ether/acetone = 30:1, v/v) afforded a white solid in 60% yield (36.9 mg). M.p.: 124-126 °C. ^{1}H NMR ($CDCl_3$, 400 MHz): δ = 6.87-6.92 (m, 2H), 7.09-7.18 (m, 10H), 7.30 (td, J = 8.0 Hz, J = 2.0 Hz, 1H), 7.44-7.46 (m, 2H), 7.52 (td, J = 8.4 Hz, J = 6.4 Hz, 1H), 8.31 (dd, J = 4.8 Hz, J = 0.8 Hz, 1H) ppm. ^{13}C NMR ($CDCl_3$, 100 MHz): δ = 121.0, 126.4, 126.9, 127.8, 128.3, 129.6, 129.8, 135.0, 138.7, 141.7, 142.0, 148.6, 159.1 ppm. HRMS (ESI$^+$): calcd for $C_{23}H_{17}NNa$ $[M+Na]^+$ 330.1259, found 330.1252. *Characterization data were consistent with literature values.*[10]

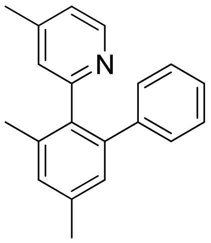

2-(3,5-Dimethyl-[1,1'-biphenyl]-2-yl)-4-methylpyridine (4ha)

2-(2,4-Dimethylphenyl)-4-methylpyridine (39.4 mg, 0.2 mmol), phenylboronic acid (73.2 mg, 0.6 mmol), $AuBr_3$ (4.4 mg, 0.01 mmol), NFSI (189.2 mg, 0.6 mmol) and *t*-BuOH (2.0 mL) at 130 °C for 24 h. Purification via neutral alumina column chromatography (petroleum ether/acetone = 30:1, v/v) afforded colorless oil in 52% yield (28.4 mg). ^{1}H NMR ($CDCl_3$, 400 MHz): δ = 2.13 (s, 3H), 2.15 (s, 3H), 2.40 (s, 3H), 6.69 (s, 1H), 6.89 (d, J = 5.2 Hz, 1H), 7.07-7.14 (m, 7H), 8.46 (d, J = 4.8 Hz, 1H) ppm. ^{13}C NMR ($CDCl_3$, 100 MHz): δ = 20.5, 21.0, 21.3, 122.2, 126.2, 126.8, 127.6, 128.4, 129.7, 130.2, 136.7, 136.9, 137.5, 141.3, 142.0, 146.6, 148.6, 159.5 ppm. HRMS (ESI$^+$): calcd for $C_{20}H_{20}N$ $[M+H]^+$ 274.1596, found 274.1596. Anal. Calcd for $C_{20}H_{19}N$ (%): C, 87.87; H, 7.01; N, 5.12, found: C, 87.53; H, 7.04; N, 5.05.

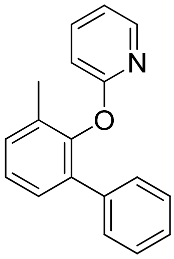

2-((3-Methyl-[1,1'-biphenyl]-2-yl)oxy)pyridine (4ia)

2-(*o*-Tolyloxy)pyridine (37.0 mg, 0.2 mmol), phenylboronic acid (73.2 mg, 0.6 mmol), $AuBr_3$ (4.4 mg, 0.01 mmol), NFSI (189.2 mg, 0.6 mmol) and *t*-BuOH (2.0 mL) at 130 °C for 24 h. Purification via neutral alumina column chromatography (CH_2Cl_2/hexane = 3:1, v/v) afforded a white solid in 71% yield (37.1 mg). M.p.: 57-59 °C. ^{1}H NMR ($CDCl_3$, 400 MHz): δ = 2.22 (s, 3H), 6.70 (d, J = 8.4 Hz, 1H), 6.84 (dd, J = 6.4 Hz, J = 5.2 Hz, 1H), 7.22-7.33 (m, 6H), 7.45 (d, J = 6.8 Hz, 2H), 7.51-7.56 (m, 1H), 8.09 (dd, J = 4.8 Hz, J = 1.2 Hz, 1H) ppm. ^{13}C NMR ($CDCl_3$, 100

MHz): δ = 17.0, 110.3, 117.6, 125.7, 127.0, 128.0, 128.9, 129.2, 130.6, 132.1, 135.6, 138.4, 139.1, 147.7, 149.0, 163.4 ppm. HRMS (ESI$^+$): calcd for $C_{18}H_{15}NNaO$ [M+Na]$^+$ 284.1051, found 284.1050. Anal. Calcd for $C_{18}H_{15}NO$ (%): C, 82.73; H, 5.79; N, 5.36, found: C, 82.71; H, 5.84; N, 5.32.

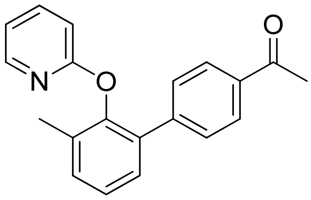

1-(3'-Methyl-2'-(pyridin-2-yloxy)-[1,1'-biphenyl]-4-yl)ethanone (4if)

2-(*o*-Tolyloxy)pyridine (37.0 mg, 0.2 mmol), 4-acetylphenylboronic acid (98.4 mg, 0.6 mmol), $AuBr_3$ (4.4 mg, 0.01 mmol), NFSI (189.2 mg, 0.6 mmol) and *t*-BuOH (2.0 mL) at 130 °C for 24 h. Purification via neutral alumina column chromatography (CH_2Cl_2/hexane = 3:1, v/v) afforded a white solid in 63% yield (38.2 mg). M.p.: 156-158 °C. ^{1}H NMR ($CDCl_3$, 400 MHz): δ = 2.21 (s, 3H), 2.58 (s, 3H), 6.70 (d, J = 8.4 Hz, 1H), 6.84 (dd, J = 6.4 Hz, J = 5.2 Hz, 1H), 7.26-7.35 (m, 3H), 7.52-7.56 (m, 3H), 7.87 (d, J = 8.4 Hz, 2H), 8.06 (dd, J = 5.2 Hz, J = 1.6 Hz, 1H) ppm. ^{13}C NMR ($CDCl_3$, 100 MHz): δ = 17.0, 26.7, 110.3, 117.8, 125.9, 128.2, 128.6, 129.4, 131.4, 132.4, 134.6, 135.7, 139.3, 143.5, 147.7, 149.0, 163.2, 198.0 ppm. HRMS (ESI$^+$): calcd for $C_{20}H_{18}NO_2$ [M+H]$^+$ 304.1338, found 304.1335. Anal. Calcd for $C_{20}H_{17}NO_2$ (%): C, 79.19; H, 5.65; N, 4.62, found: C, 79.34; H, 5.67; N, 4.66.

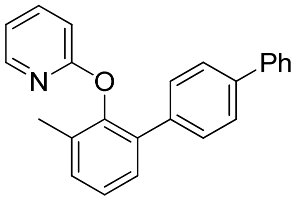

2-((3-Methyl-[1,1':4',1''-terphenyl]-2-yl)oxy)pyridine (4ii)

2-(*o*-Tolyloxy)pyridine (37.0 mg, 0.2 mmol), [1,1'-biphenyl]-4-ylboronic acid (118.9 mg, 0.6 mmol), $AuBr_3$ (4.4 mg, 0.01 mmol), NFSI (189.2 mg, 0.6 mmol) and *t*-BuOH (2.0 mL) at 130 °C for 24 h. Purification via neutral alumina column chromatography (CH_2Cl_2/hexane = 3:1, v/v) afforded colorless oil in 70% yield (47.2 mg). ^{1}H NMR

($CDCl_3$, 400 MHz): δ = 2.21 (s, 3H), 6.71 (dd, J = 8.0 Hz, J = 0.8 Hz, 1H), 6.81-6.84 (m, 1H), 7.25-7.36 (m, 4H), 7.40-7.44 (m, 2H), 7.50-7.54 (m, 5H), 7.57-7.59 (m, 2H), 8.08-8.09 (m, 1H) ppm. ^{13}C NMR ($CDCl_3$, 100 MHz): δ = 17.1, 110.3, 117.6, 125.8, 126.7, 127.1, 127.3, 128.8, 129.6, 130.7, 132.2, 135.2, 137.4, 139.2, 139.8, 140.9, 147.8, 149.2, 163.5 ppm. HRMS (ESI^+): calcd for $C_{24}H_{20}NO$ $[M+H]^+$ 338.1545, found 338.1540. Anal. Calcd for $C_{24}H_{19}NO$ (%): C, 85.43; H, 5.68; N, 4.15, found: C, 85.05; H, 5.82; N, 4.08.

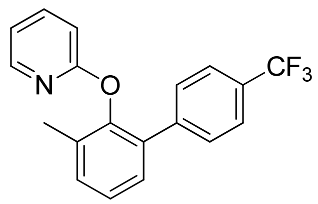

2-((3-Methyl-4'-(trifluoromethyl)-[1,1'-biphenyl]-2-yl)oxy)pyridine (4ij)

2-(*o*-Tolyloxy)pyridine (37.0 mg, 0.2 mmol), 4-trifluoromethylphenylboronic acid (114.0 mg, 0.6 mmol), $AuBr_3$ (4.4 mg, 0.01 mmol), NFSI (189.2 mg, 0.6 mmol) and *t*-BuOH (2.0 mL) at 130 °C for 24 h. Purification via neutral alumina column chromatography (CH_2Cl_2/hexane = 3:1, v/v) afforded a white solid in 89% yield (58.6 mg). M.p.: 85-87 °C. ^1H NMR ($CDCl_3$, 400 MHz): δ = 2.21 (s, 3H), 6.71 (d, J = 8.4 Hz, 1H), 6.85-6.88 (m, 1H), 7.29 (d, J = 8.4 Hz, 2H), 7.32-7.37 (m, 1H), 7.52-7.58 (m, 5H), 8.06-8.08 (m, 1H) ppm. ^{13}C NMR ($CDCl_3$, 100 MHz): δ = 17.0, 110.4, 117.9, 124.91, 124.94, 124.98, 125.0, 125.9, 128.7, 129.5, 131.4, 132.4, 134.4, 139.4, 142.2, 147.7, 149.1, 163.2 ppm. HRMS (ESI^+): calcd for $C_{19}H_{15}F_3NO$ $[M+H]^+$ 330.1106, found 330.1106. Anal. Calcd for $C_{19}H_{14}F_3NO$ (%): C, 69.30; H, 4.28; N, 4.25, found: C, 69.25; H, 3.95; N, 4.35.

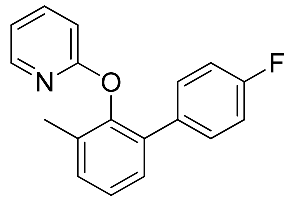

2-((4'-Fluoro-3-methyl-[1,1'-biphenyl]-2-yl)oxy)pyridine (4il)

2-(*o*-Tolyloxy)pyridine (37.0 mg, 0.2 mmol), 4-fluorophenylboronic acid (84.0 mg, 0.6 mmol), $AuBr_3$ (4.4 mg, 0.01 mmol), NFSI (189.2 mg, 0.6 mmol) and *t*-BuOH (2.0 mL) at 130 °C for 24 h. Purification via neutral alumina column chromatography (CH_2Cl_2/hexane = 3:1, v/v) afforded a white solid in 82% yield (45.8 mg). M.p.: 87-89 °C. ^1H NMR ($CDCl_3$, 400 MHz): δ = 2.20 (s, 3H), 6.68 (d, J = 8.4 Hz, 1H), 6.85 (dd, J = 6.8 Hz, J = 5.2 Hz, 1H), 6.93-6.99 (m, 2H), 7.23-7.32 (m, 3H), 7.37-7.42 (m, 2H), 7.52-7.56 (m, 1H), 8.07 (dd, J = 4.8 Hz, J = 1.6 Hz, 1H) ppm. ^{13}C NMR ($CDCl_3$, 100 MHz): δ = 17.0, 110.3, 114.9 (d, J = 21.2 Hz), 117.7, 125.8, 128.7, 130.8 (d, J = 4.9 Hz), 130.9, 132.2, 134.4 (d, J = 3.2 Hz), 134.7, 139.2, 147.7, 149.1, 162.1 (d, J = 244.3 Hz), 163.4 ppm. HRMS (ESI$^+$): calcd for $C_{18}H_{15}FNO$ $[M+H]^+$ 280.1138, found 280.1136. Anal. Calcd for $C_{18}H_{14}FNO$ (%): C, 77.40; H, 5.05; N, 5.01, found: C, 77.17; H, 5.00; N, 5.09.

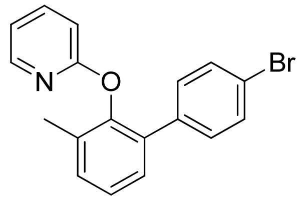

2-((4'-Bromo-3-methyl-[1,1'-biphenyl]-2-yl)oxy)pyridine (4in)

2-(*o*-Tolyloxy)pyridine (37.0 mg, 0.2 mmol), 4-bromophenylboronic acid (120.0 mg, 0.6 mmol), $AuBr_3$ (4.4 mg, 0.01 mmol), NFSI (189.2 mg, 0.6 mmol) and *t*-BuOH (2.0 mL) at 130 °C for 24 h. Purification via neutral alumina column chromatography (CH_2Cl_2/hexane = 3:1, v/v) afforded a white solid in 70% yield (47.5 mg). M.p.: 115-116 °C. ^1H NMR ($CDCl_3$, 400 MHz): δ = 2.17 (s, 3H), 6.67 (d, J = 8.4 Hz, 1H), 6.82-6.85 (m, 1H), 7.24 (d, J = 4.8 Hz, 2H), 7.28-7.30 (m, 3H), 7.37-7.39 (m, 2H), 7.51-7.56 (m, 1H), 8.04 (dd, J = 4.8 Hz, J = 1.2 Hz, 1H) ppm. ^{13}C NMR ($CDCl_3$, 100 MHz): δ = 17.0, 110.3, 117.8, 121.4, 125.8, 128.6, 130.9, 131.0, 131.2, 132.3, 134.5, 137.4, 139.3, 147.7, 149.0, 163.3 ppm. HRMS (ESI$^+$): calcd for $C_{18}H_{15}BrNO$ $[M+H]^+$ 340.0337, found 340.0338. Anal. Calcd for $C_{18}H_{14}BrNO$ (%): C, 63.55; H, 4.15; N, 4.12, found: C, 63.75; H, 4.16; N, 4.15.

VIII. Removal of the directing group[12]

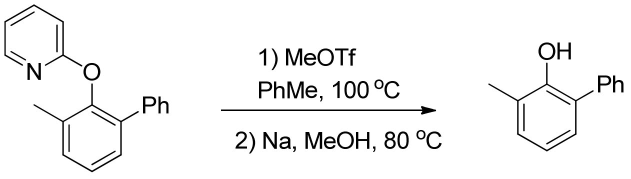

4ia **9**

A three necked flask with a magnetic stirring bar was charged with **4ia** (65.5 mg. 0.25 mmol), trifluoromethanesulfonate (100 μL, 0.88 mmol) and toluene under N_2. Then the reaction mixture was stirred at 100 °C for 2 h. The reaction mixture was then cooled to ambient temperature and the solvent was evaporated in vacuum to give a white solid. Under an N_2 atmosphere, the solid in methanol (2.5 mL) was added to a solution of sodium (150 mg, 12 mmol) in methanol (8 mL). The reaction mixture was stirred at 80 °C for 30 min. After the reaction mixture was cooled to room temperature, methanol was removed in vacuum. The resulting reaction mixture was added water (40 mL), extracted with EtOAc and dried over Na_2SO_4. The organic layer was concentrated and the resulting residue was purified by silica gel column chromatography (petroleum ether/EtOAc = 15/1, v/v) to provide the desired product **9** as colorless oil in 77% yield (35.3 mg). ^{1}H NMR ($CDCl_3$, 400 MHz): δ = 2.33 (s, 3H), 5 26 (s, 1H), 6.91 (d, J = 7.6 Hz, 1H), 7.09 (dd, J = 7.6 Hz, J = 1.2 Hz, 1H), 7.16 (d, J = 8.0 Hz, 1H), 7.39-7.44 (m, 1H), 7.46-7.53 (m, 4H) ppm. The product **9** was characterized by comparison of the ^{1}H NMR data with those reported previously.

IX. References

(1) H. V. Mierde, P. Voort and F. van der Verpoort, *Tetrahedron Lett.*, 2008, **49**, 6893.

(2) X. Rao, C. Liu, J. Qiu and Z. Jin, *Org. Biomol. Chem.*, 2012, **10**, 7875.

(3) D. Maiti and S. L. Buchwald, *J. Org. Chem.*, 2010, **75**, 1791.

(4) E. C. Constable and T. A. Leese, *J. Organomet. Chem.*, 1989, **363**, 419.

(5) E. Langseth, C. H. Görbitz, R. H. Heyn and M. Tilset, *Organometallics*, 2012, **31**, 6567.

(6) Y. Fuchita, Y. Utsunomiya and M. Yasutake, *J. Chem. Soc*., *Dalton Trans*., 2001, 2330.

(7) J. Lindh, P. J. R. Sjöberg and M. Larhed, *Angew. Chem., Int. Ed.*, 2010, **49**, 7733.

(8) D. C. Powers, M. A. L. Geibel, J. E. M. N. Klein and T. Ritter, *J. Am. Chem. Soc.*, 2009, **131**, 17050.

(9) J. Y. Kim, S. H. Park, J. Ryu, S. H. Cho, S. H. Kim and S. Chang, *J. Am. Chem. Soc.*, 2012, **134**, 9110.

(10) H. Li, W. Wei, Y. Xu, C. Zhang and X. Wan, *Chem. Commun.*, 2011, **47**, 1497.

(11) Z. Qi and X. Li, *Angew. Chem., Int. Ed.*, 2013, **52**, 8995.

(12) K. Yoshida, R. Narui and T. Imamoto, *Chem. –Eur. J.*, 2008, **14**, 9706.

X. Copies of ^{1}H and ^{13}C NMR spectra

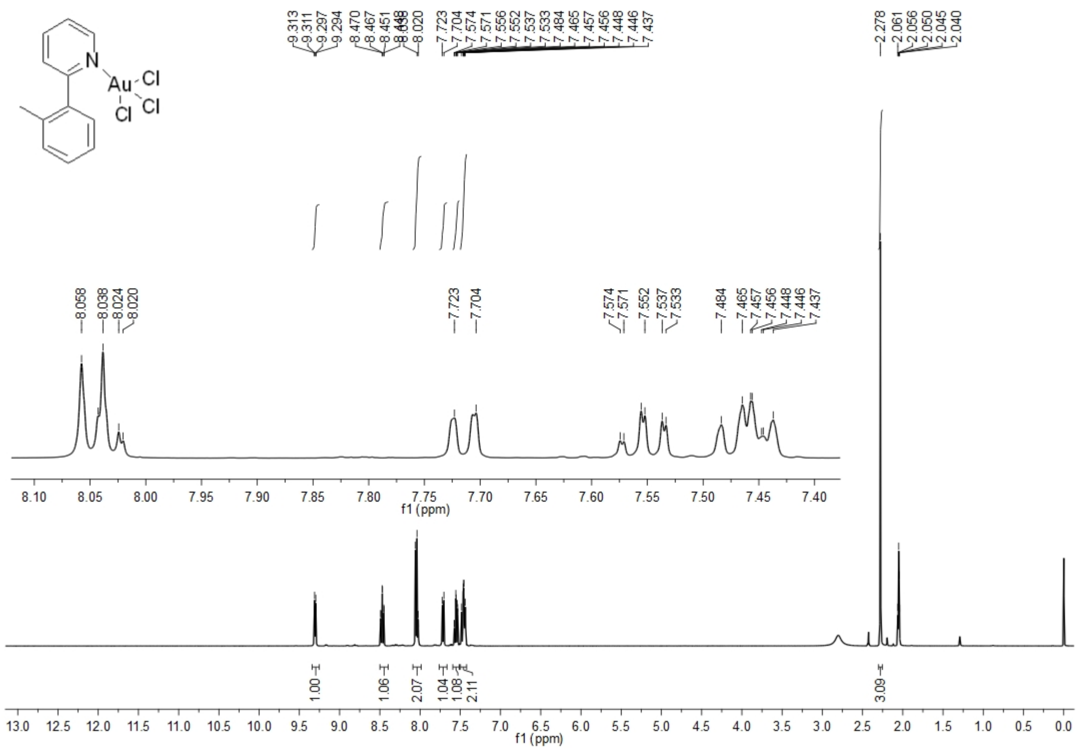

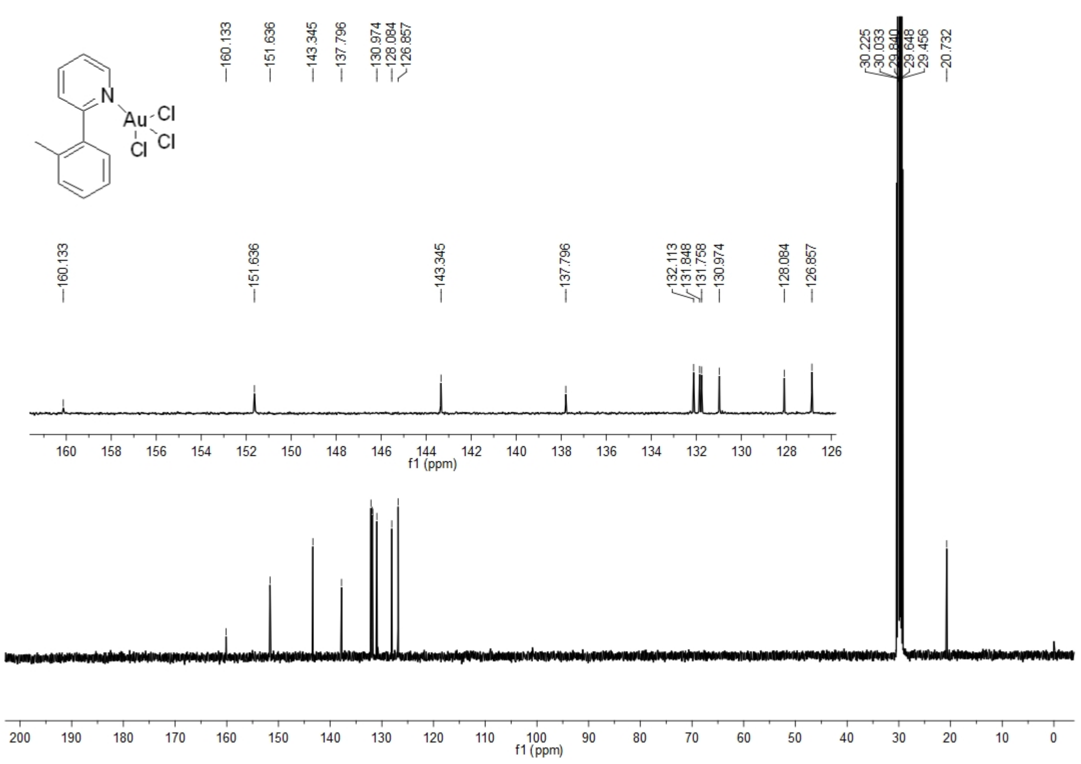

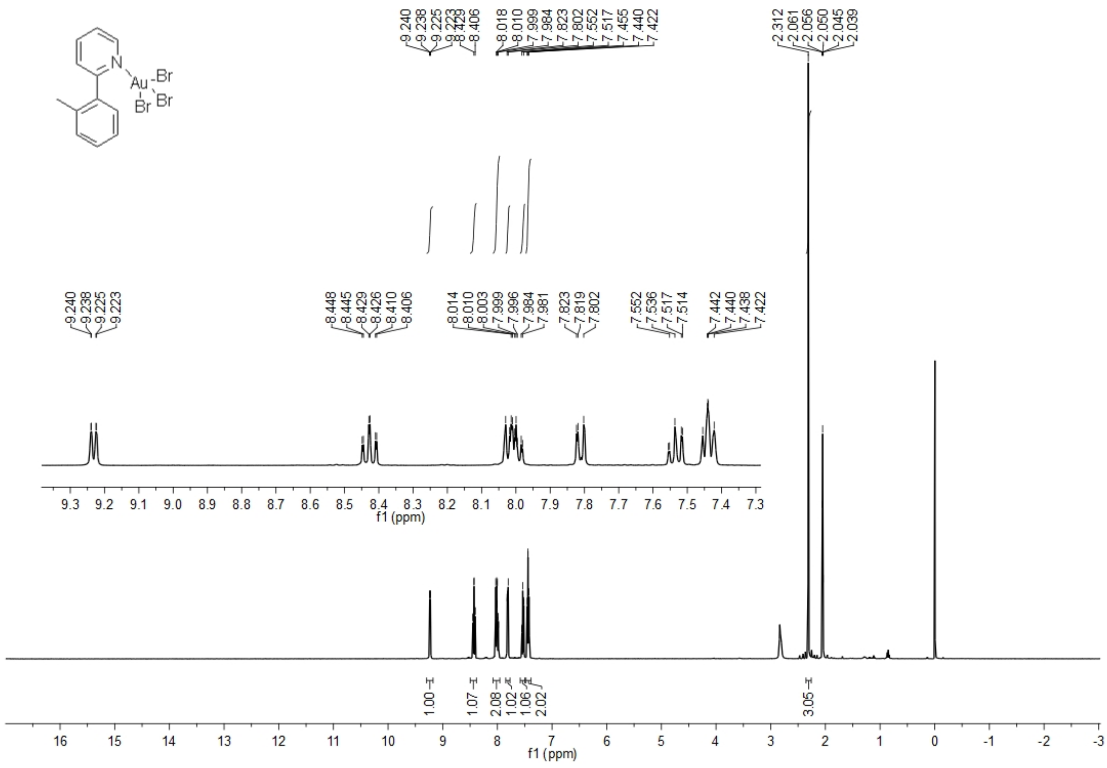

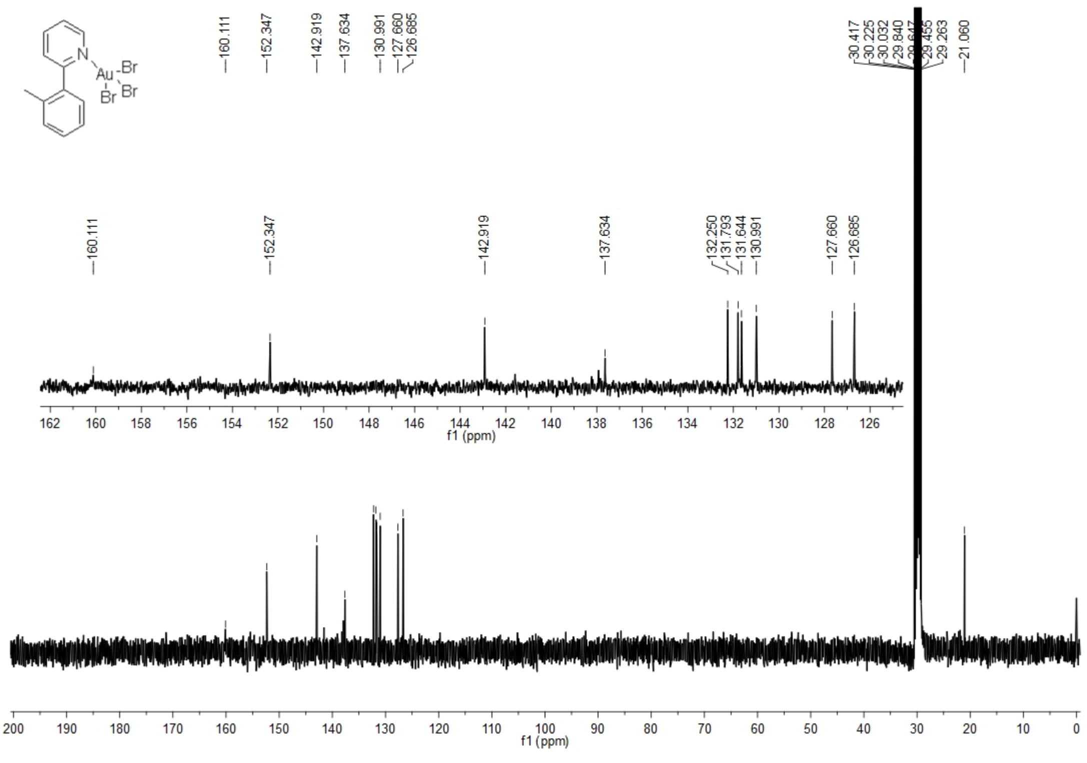

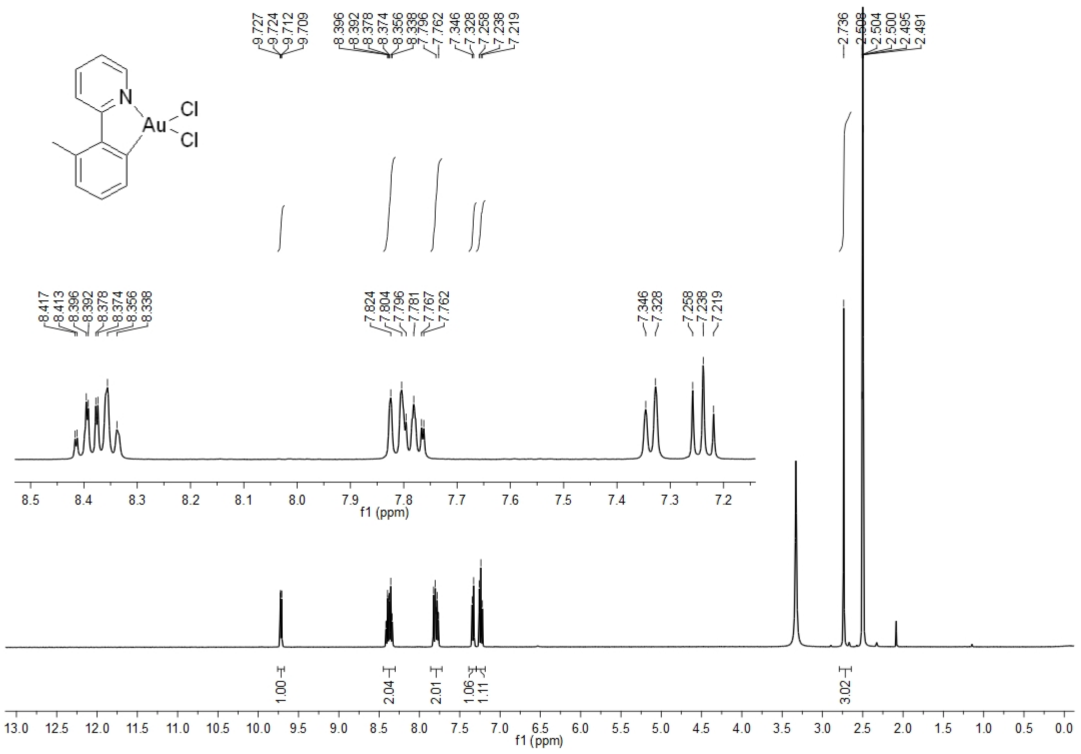

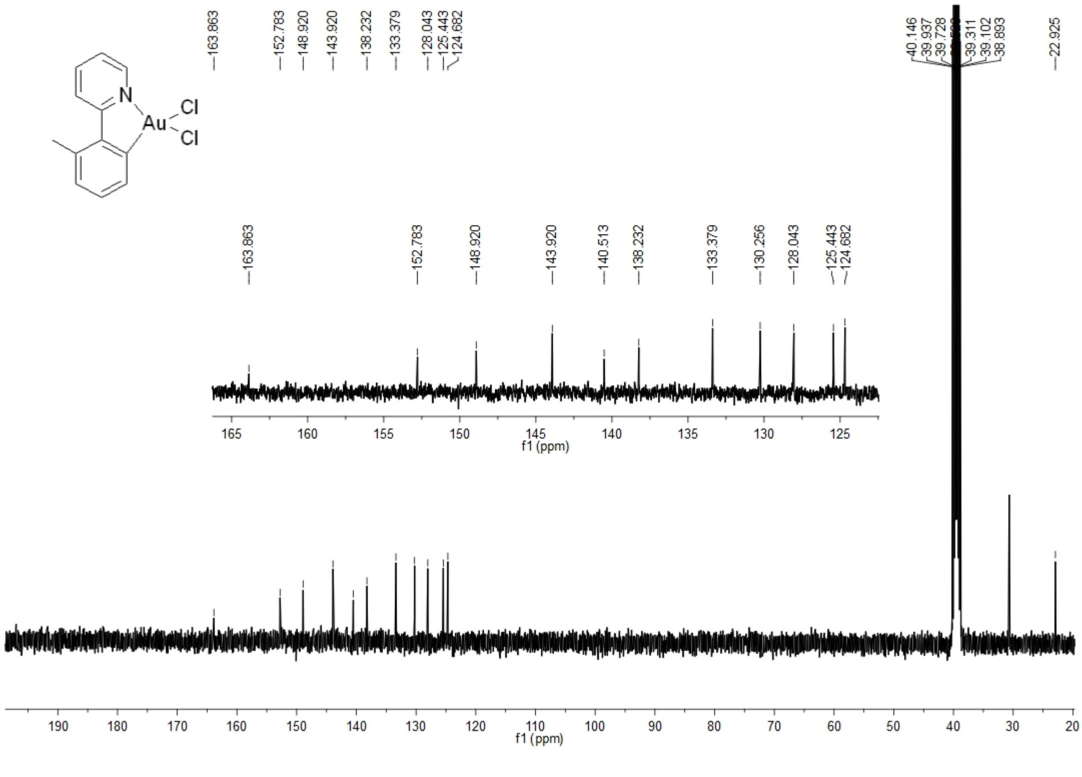

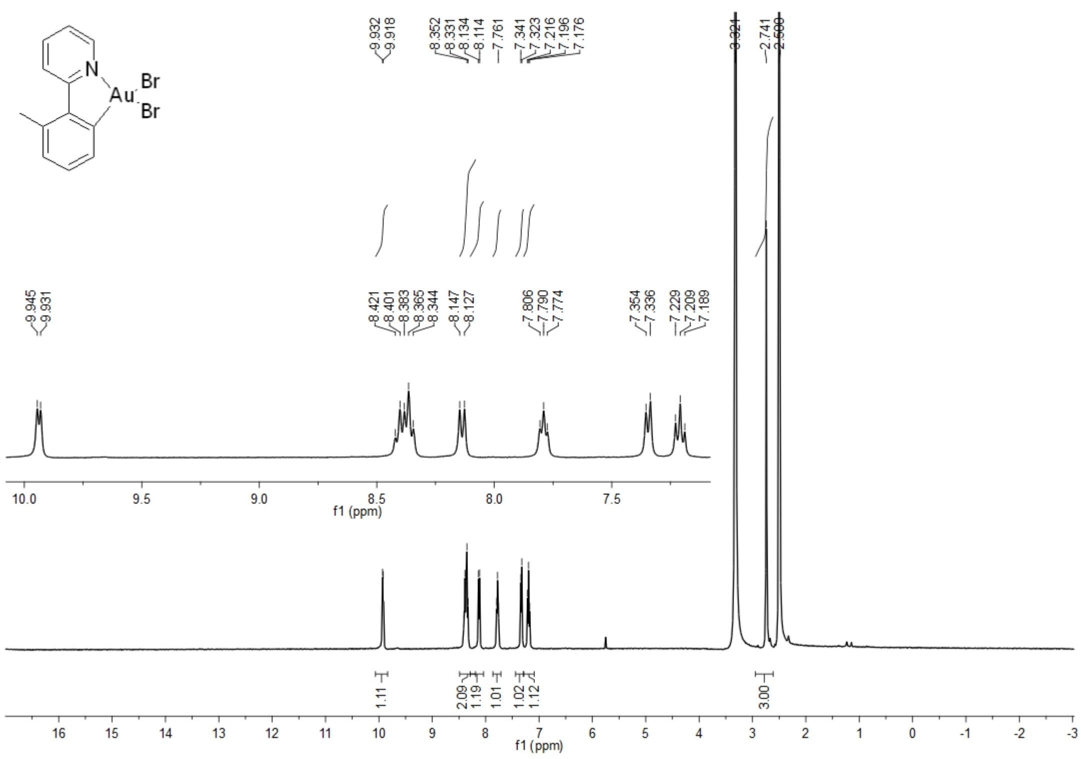

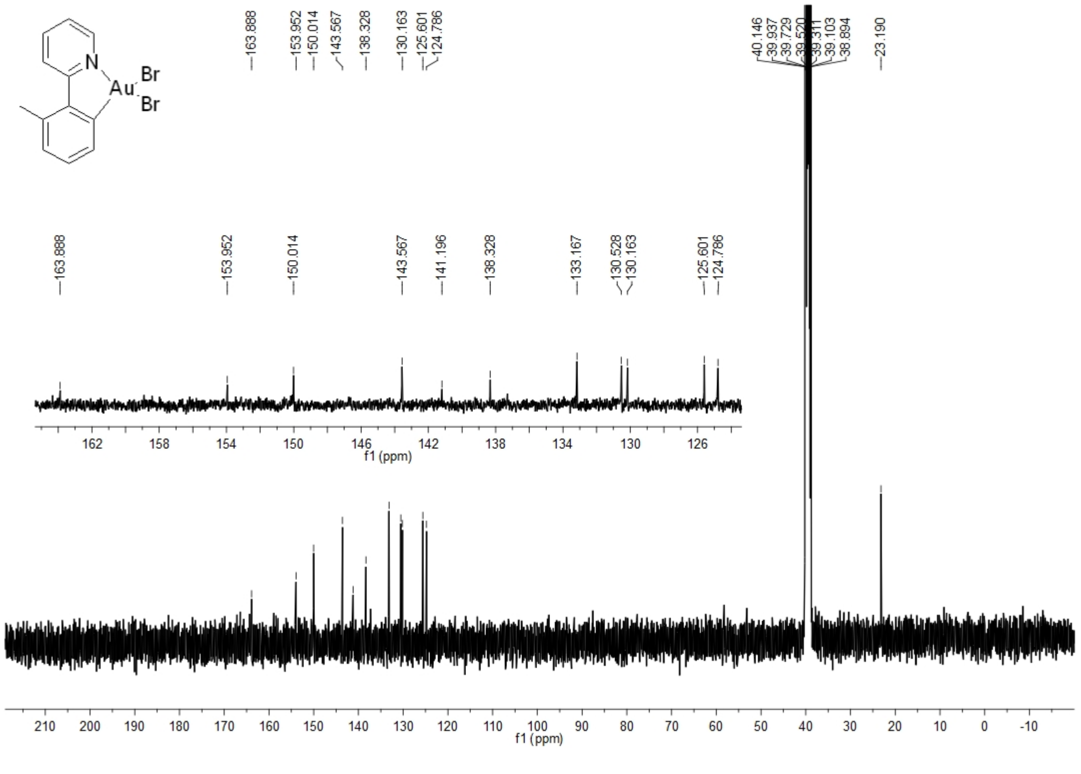

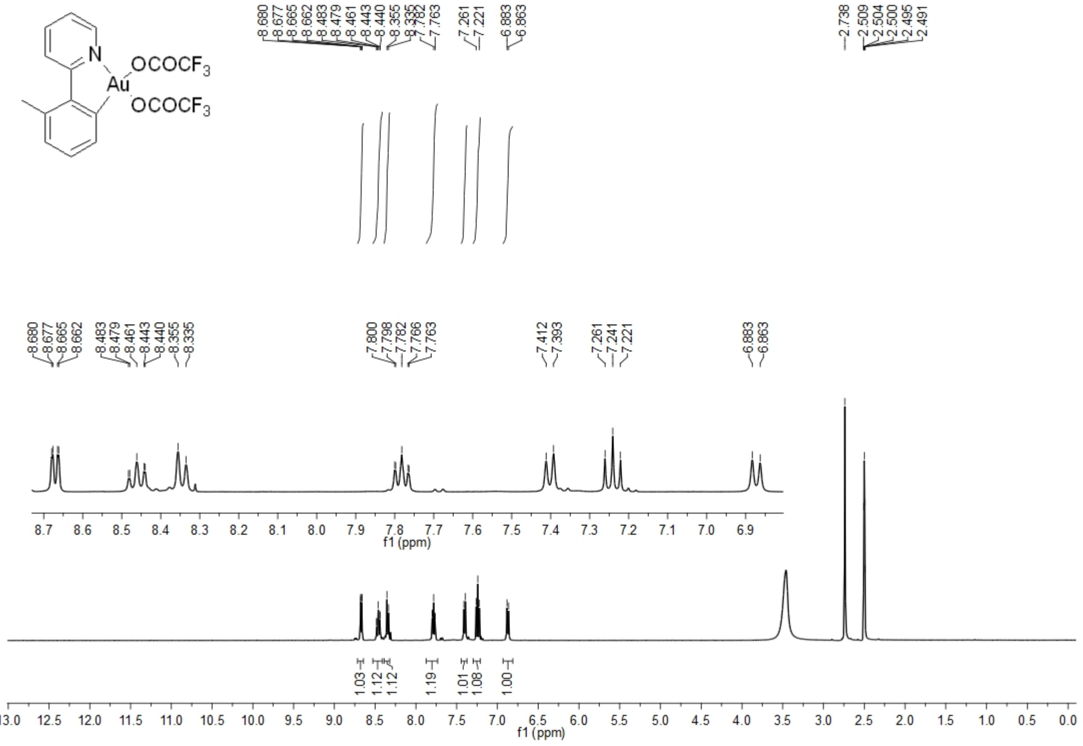
N
Au
OCOCF3
OCOCF3
8.680
8.677
8.665
8.662
8.483
8.479
8.461
8.443
8.440
8.355
8.335
7.782
7.763
7.261
7.221
6.883
6.863
2.738
2.509
2.504
2.500
2.495
2.491
8.680
8.677
8.665
8.662
8.483
8.479
8.461
8.443
8.440
8.355
8.335
7.800
7.798
7.782
7.766
7.763
7.412
7.393
7.261
7.241
7.221
6.883
6.863
f1 (ppm)
1.03
1.12
1.12
1.19
1.01
1.08
1.00
f1 (ppm)

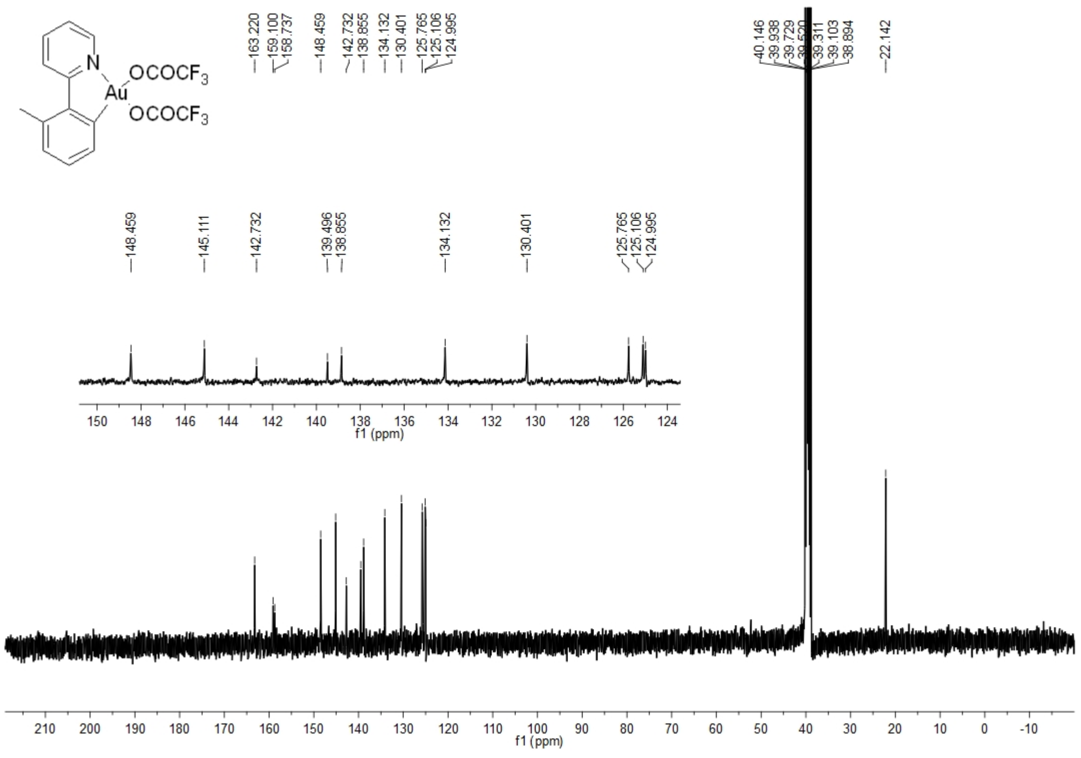
N
Au
OCOCF3
OCOCF3
163.220
159.100
158.737
148.459
142.732
138.855
134.132
130.401
125.765
125.106
124.995
40.146
39.938
39.729
39.311
39.103
38.894
22.142
148.459
145.111
142.732
139.496
138.855
134.132
130.401
125.765
125.106
124.995
f1 (ppm)
f1 (ppm)

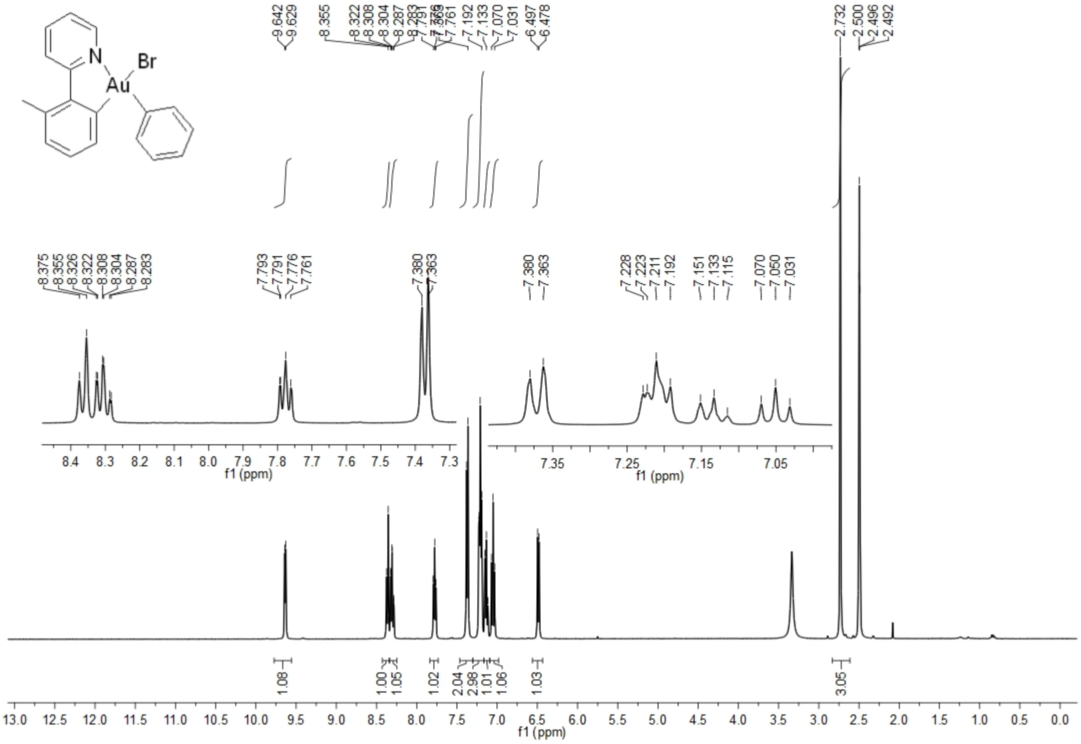
N
Br
Au
9.642
9.629
8.355
8.322
8.308
8.304
8.287
8.283
7.791
7.761
7.192
7.133
7.070
7.031
6.497
6.478
2.732
2.500
2.496
2.492
8.375
8.355
8.326
8.322
8.308
8.304
8.287
8.283
7.793
7.791
7.776
7.761
7.380
7.363
7.380
7.363
7.228
7.223
7.211
7.192
7.151
7.133
7.115
7.070
7.050
7.031
1.08
1.00
1.05
1.02
2.04
2.98
1.01
1.06
1.03
3.05
f1 (ppm)

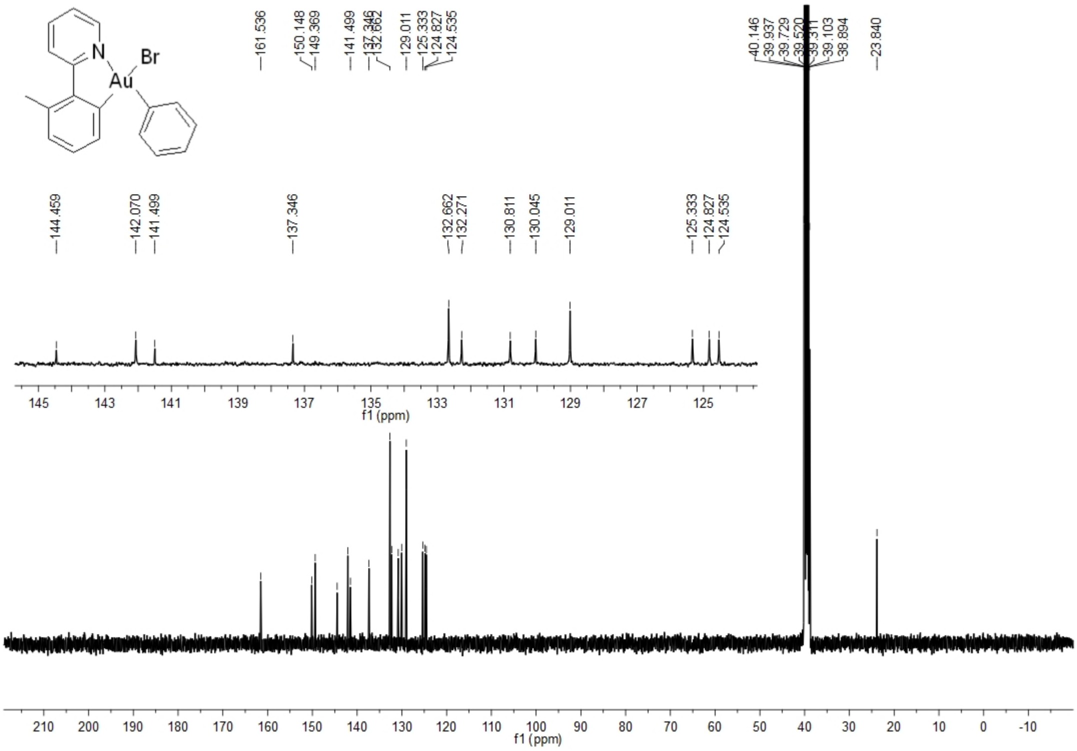
N
Br
Au
161.536
150.148
149.369
141.499
137.346
132.662
129.011
125.333
124.827
124.535
40.146
39.937
39.729
39.520
39.311
39.103
38.894
23.840
144.459
142.070
141.499
137.346
132.662
132.271
130.811
130.045
129.011
125.333
124.827
124.535
f1 (ppm)

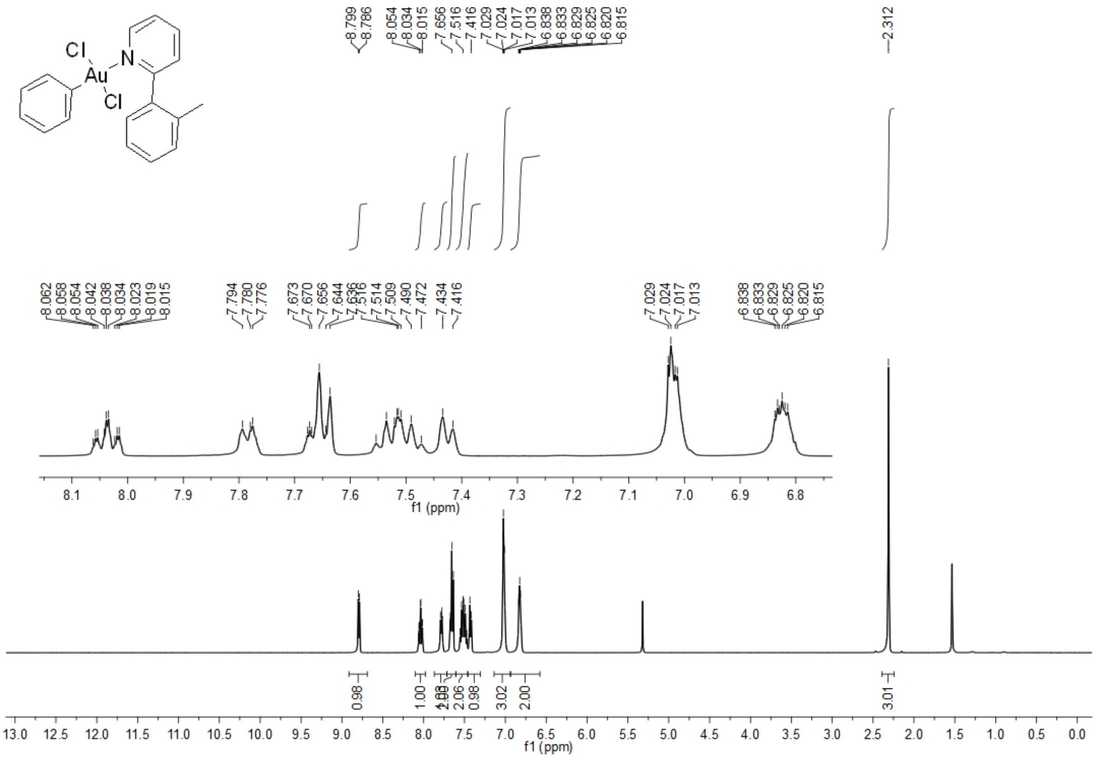
Cl
Au
N
Cl
8.799
8.786
8.054
8.034
8.015
7.656
7.516
7.416
7.029
7.024
7.017
7.013
6.838
6.833
6.829
6.825
6.820
6.815
2.312
8.062
8.058
8.054
8.042
8.038
8.034
8.023
8.019
8.015
7.794
7.780
7.776
7.673
7.670
7.656
7.644
7.636
7.516
7.514
7.509
7.490
7.472
7.434
7.416
f1 (ppm)
0.98
1.00
2.06
0.98
3.02
2.00
3.01
f1 (ppm)

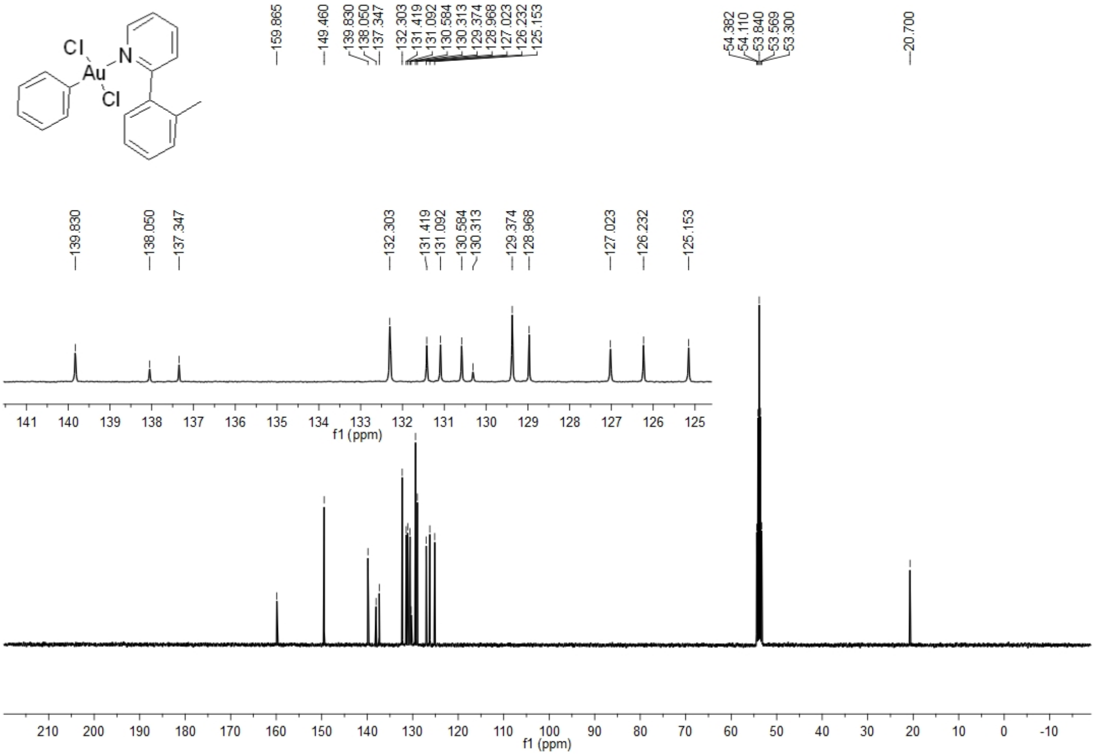
Cl
Au
N
Cl
159.865
149.460
139.830
138.050
137.347
132.303
131.419
131.092
130.584
130.313
129.374
128.968
127.023
126.232
125.153
54.382
54.110
53.840
53.569
53.300
20.700
f1 (ppm)
f1 (ppm)

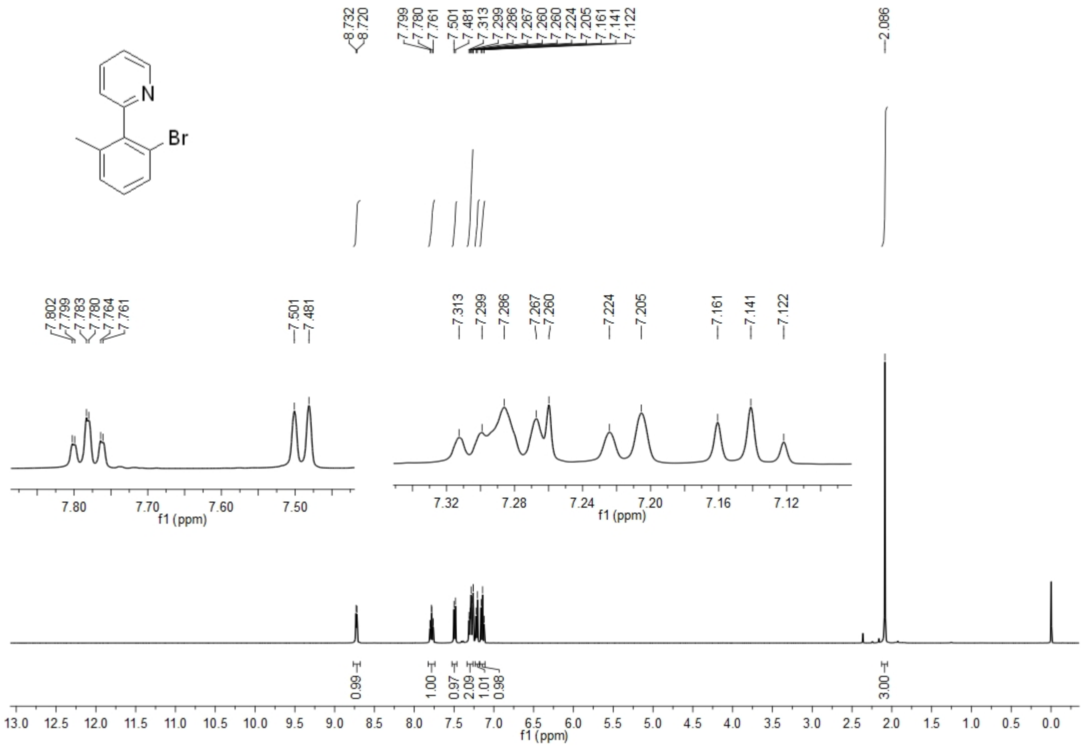

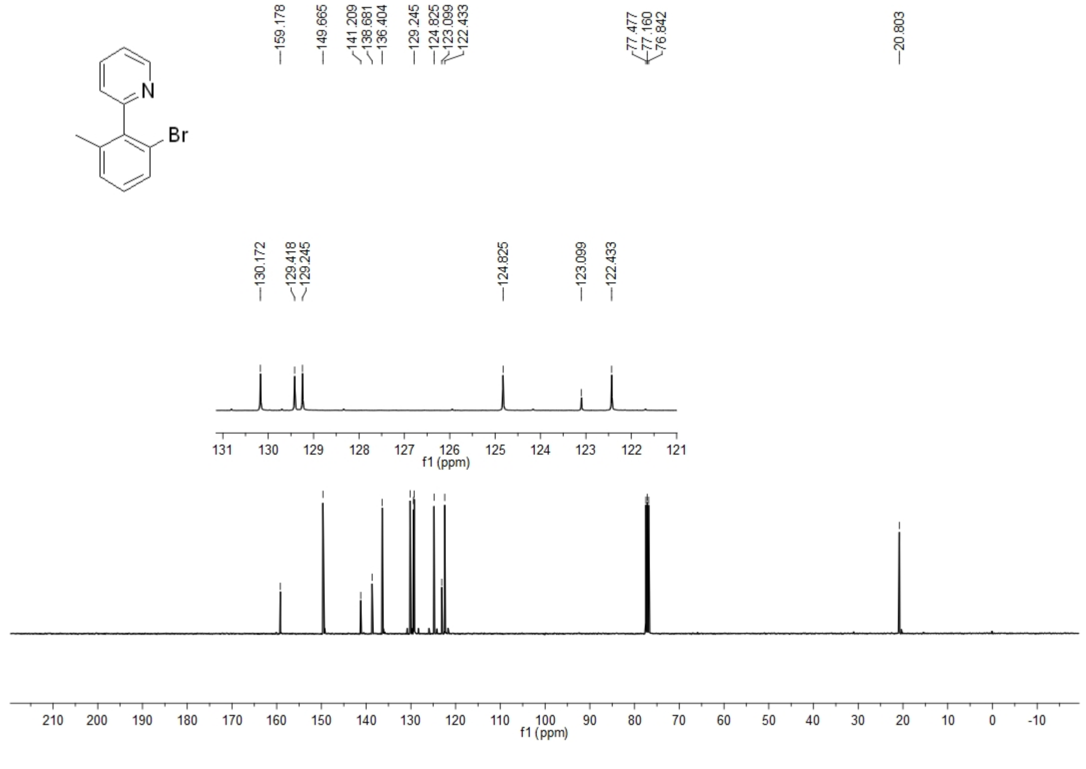

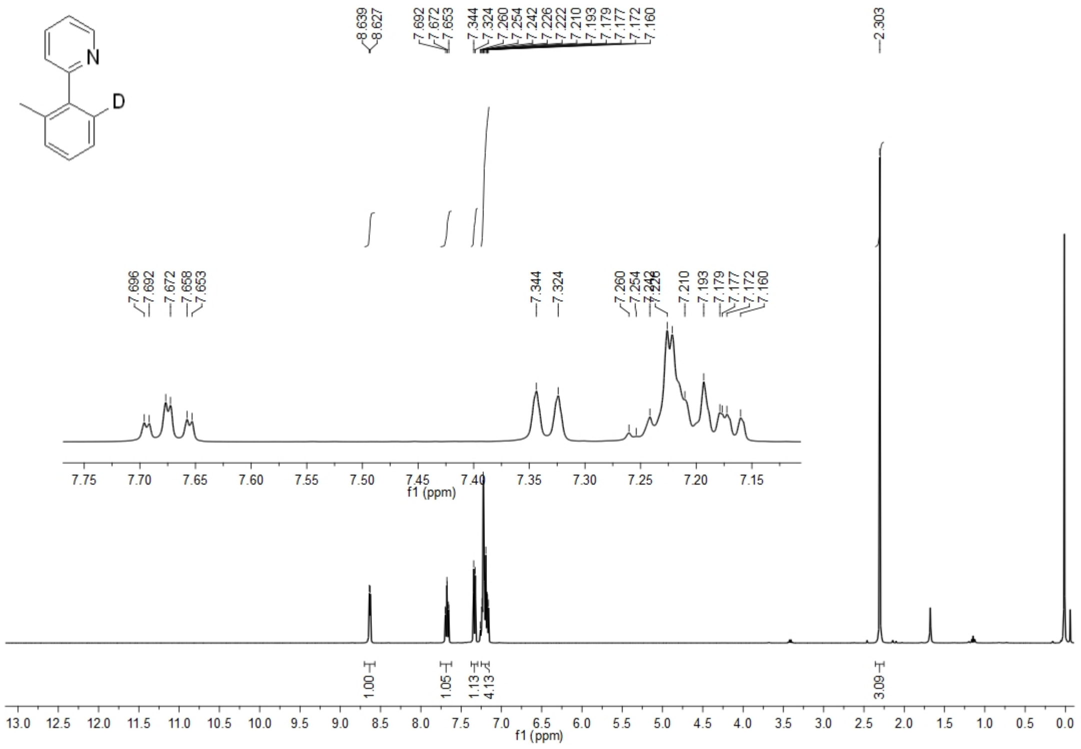

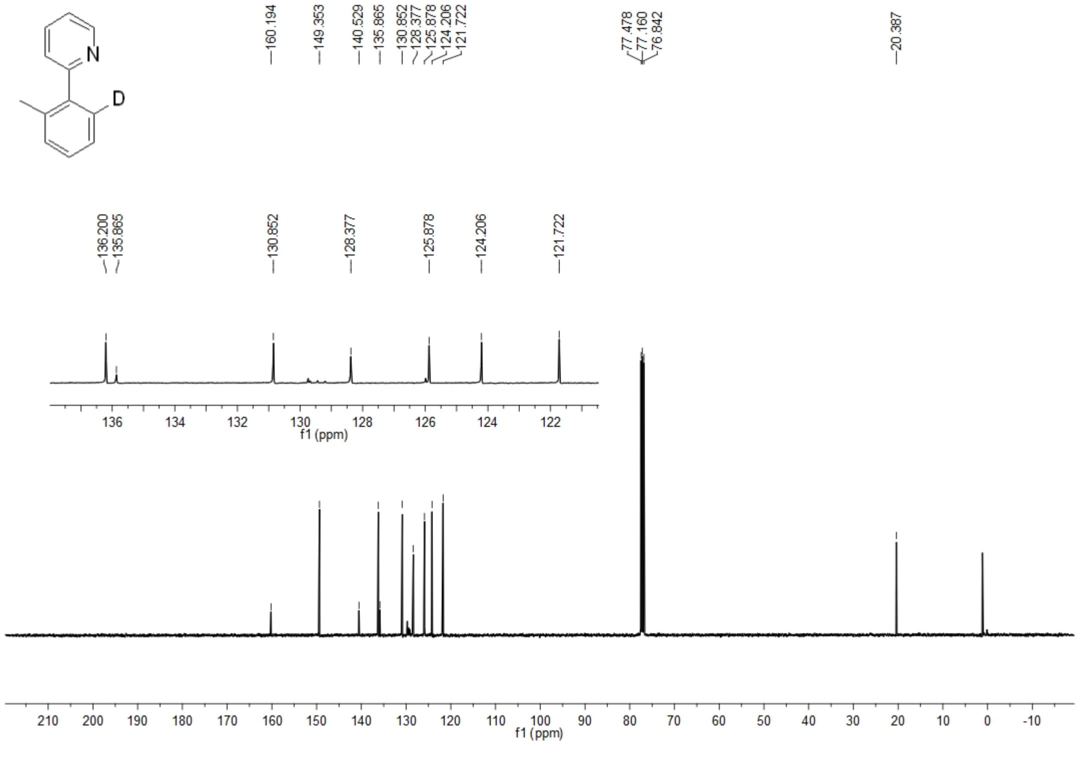

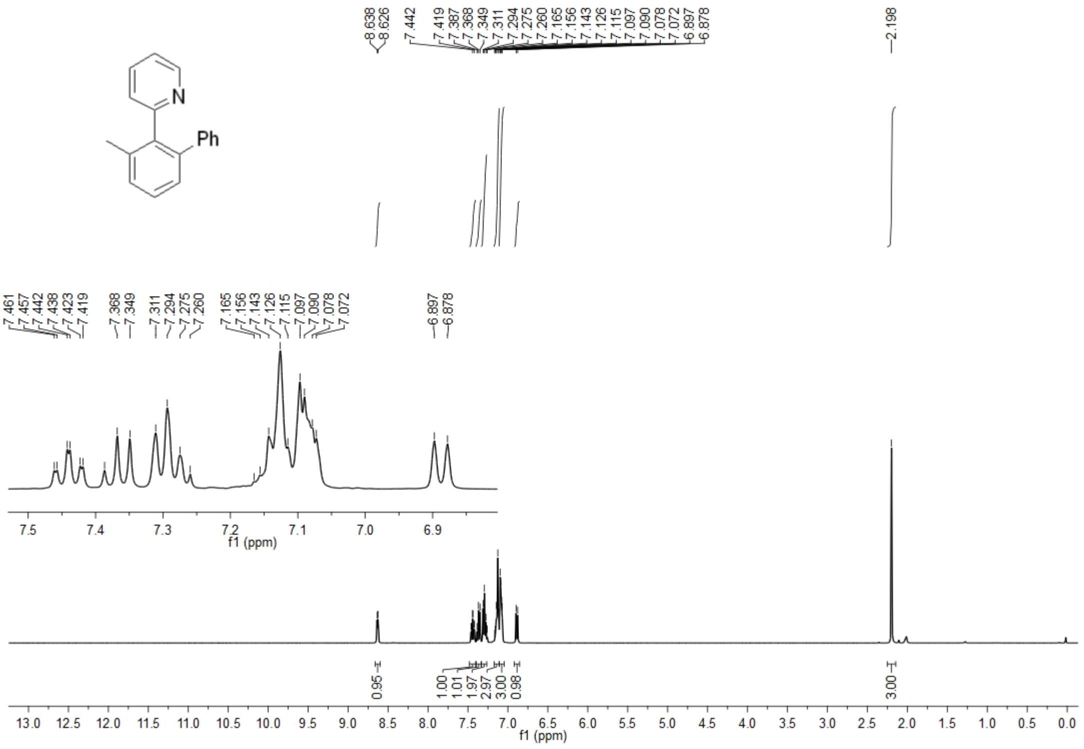

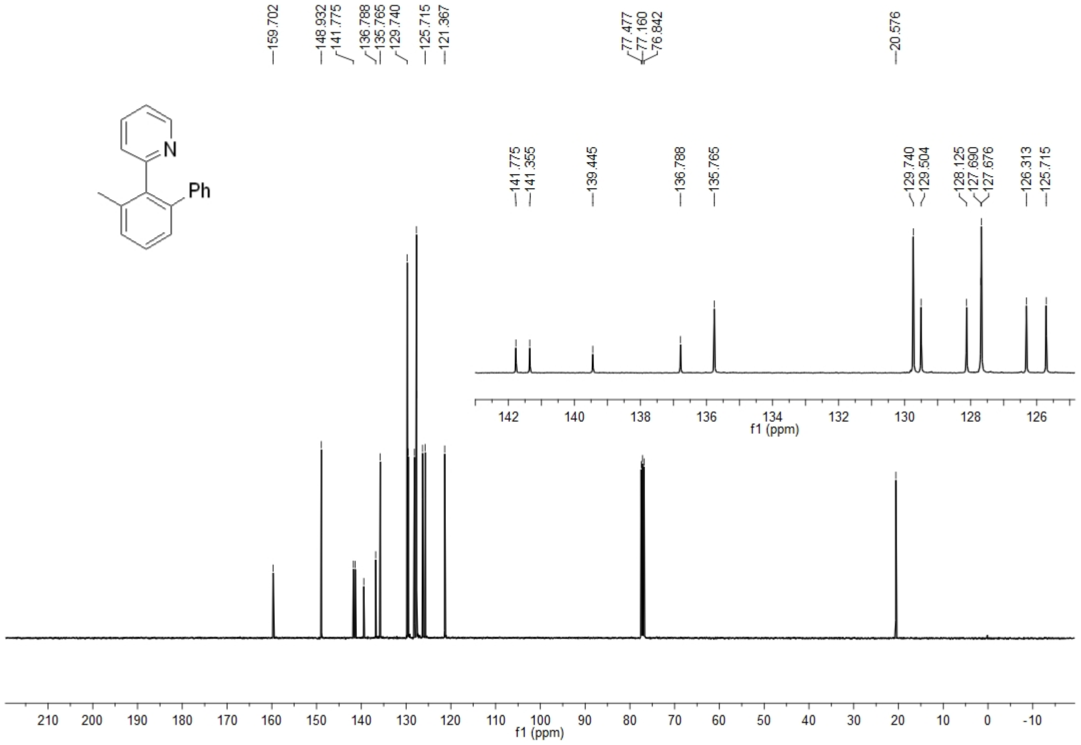

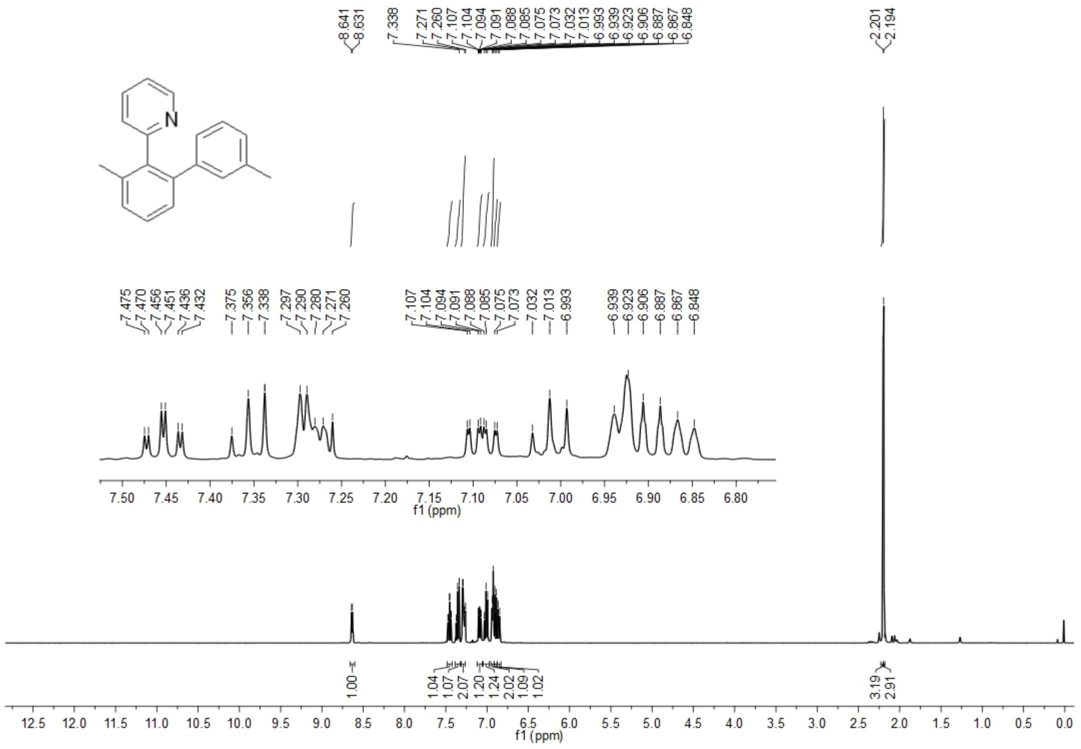

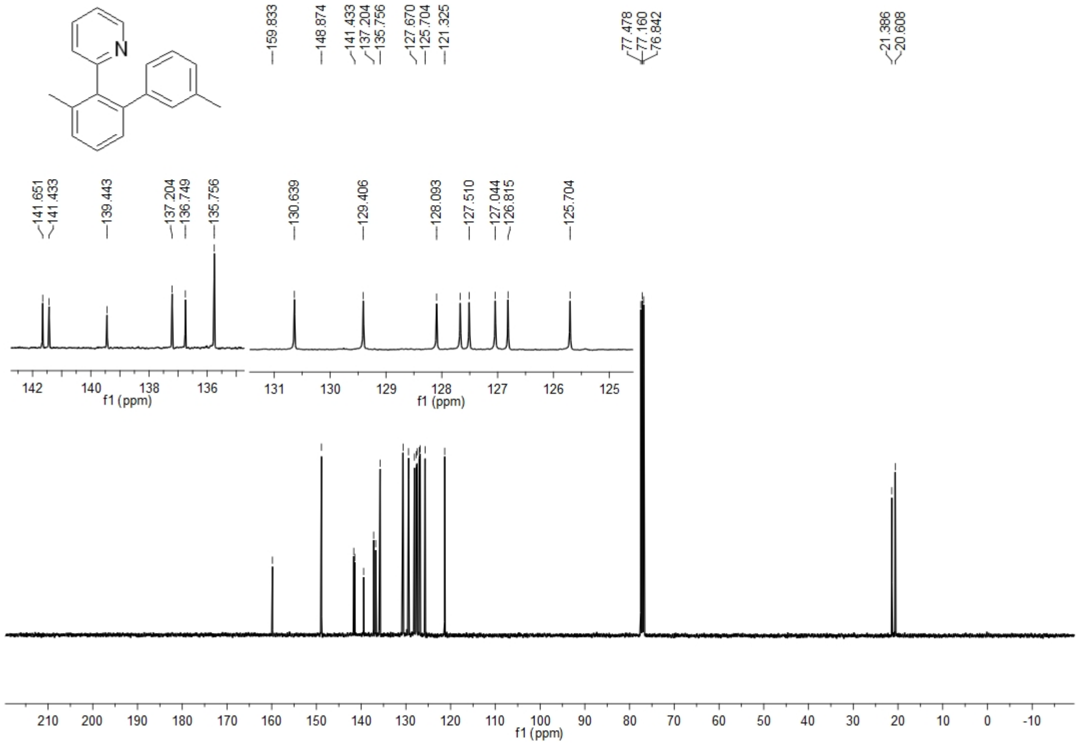

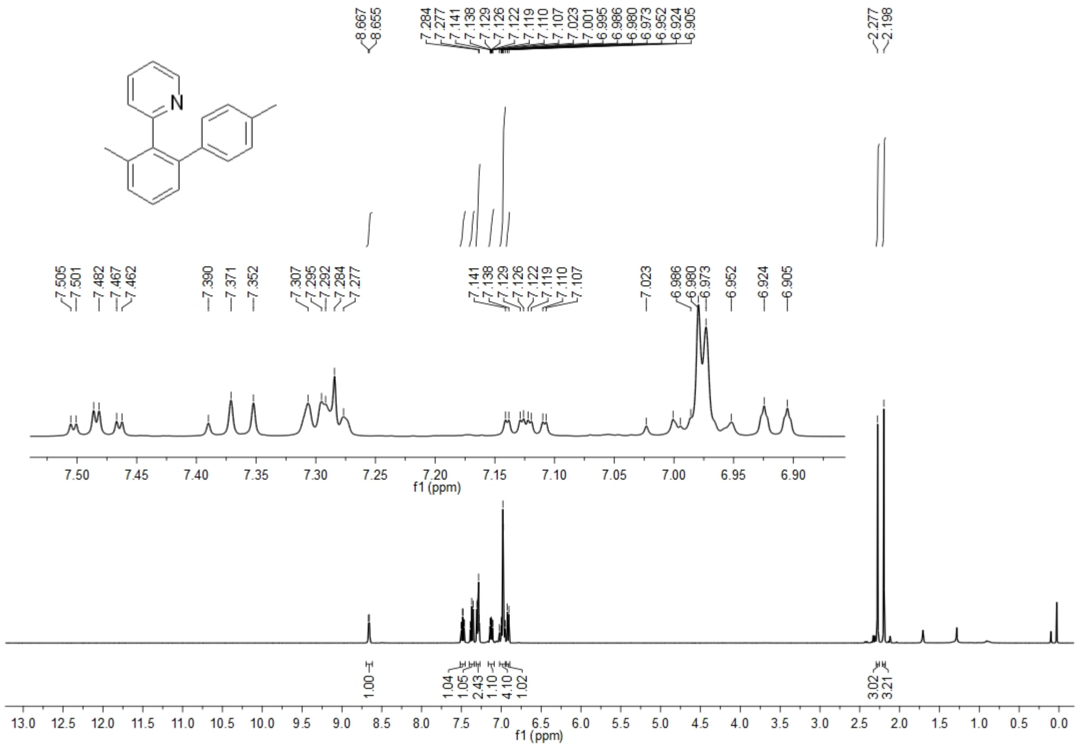
N
8.667
8.655
7.284
7.277
7.141
7.138
7.129
7.126
7.122
7.119
7.110
7.107
7.023
7.001
6.995
6.986
6.980
6.973
6.952
6.924
6.905
2.277
2.198
7.505
7.501
7.482
7.467
7.462
7.390
7.371
7.352
7.307
7.295
7.292
7.284
7.277
7.141
7.138
7.129
7.126
7.122
7.119
7.110
7.107
7.023
6.986
6.980
6.973
6.952
6.924
6.905
f1 (ppm)
1.00
1.04
1.05
2.43
1.10
4.10
1.02
3.02
3.21
f1 (ppm)

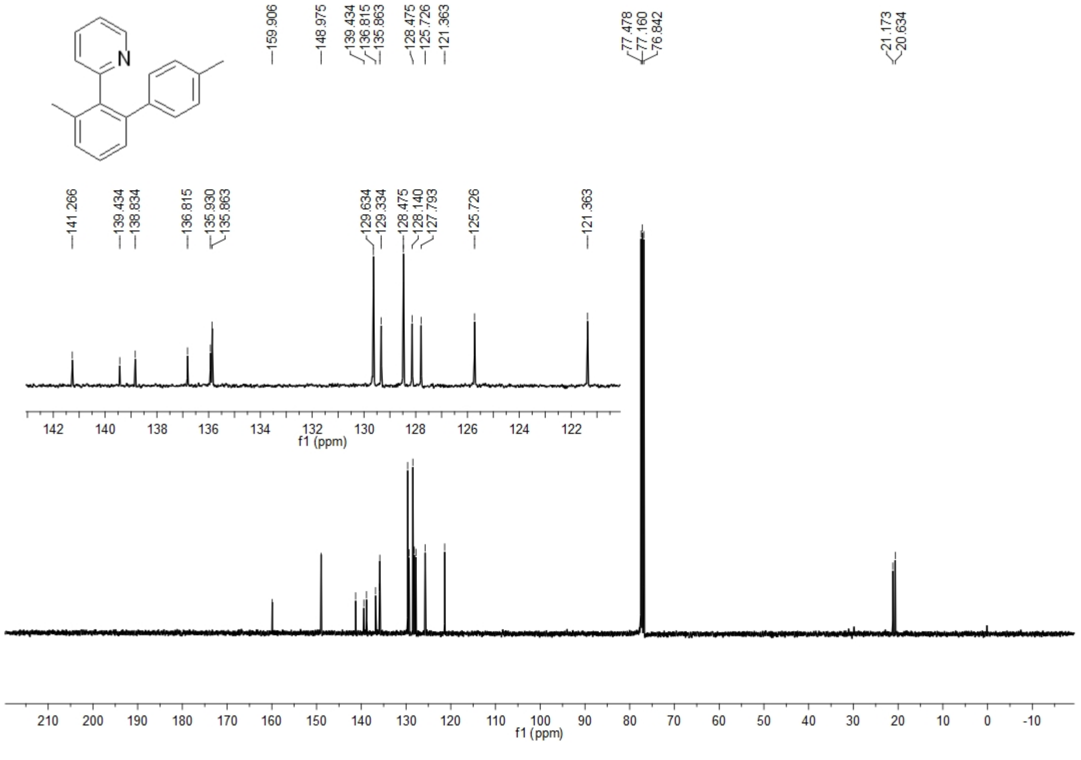
N
159.906
148.975
139.434
136.815
135.863
128.475
125.726
121.363
77.478
77.160
76.842
21.173
20.634
141.266
139.434
138.834
136.815
135.930
135.863
129.634
129.334
128.475
128.140
127.793
125.726
121.363
f1 (ppm)
f1 (ppm)

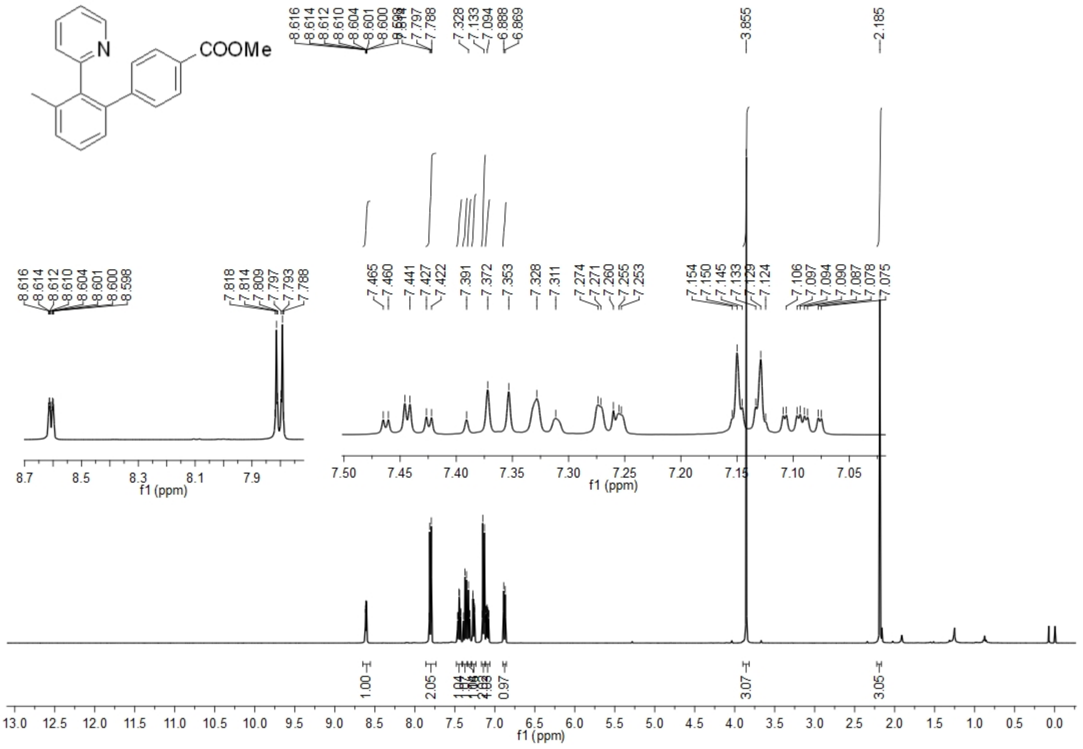

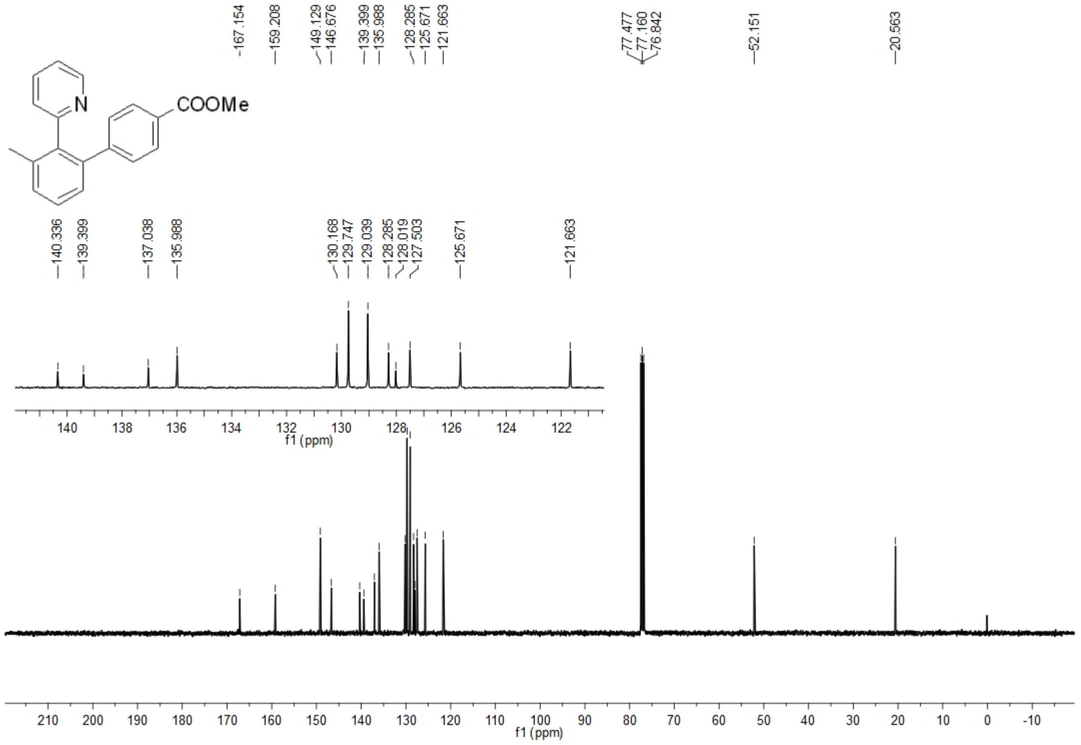

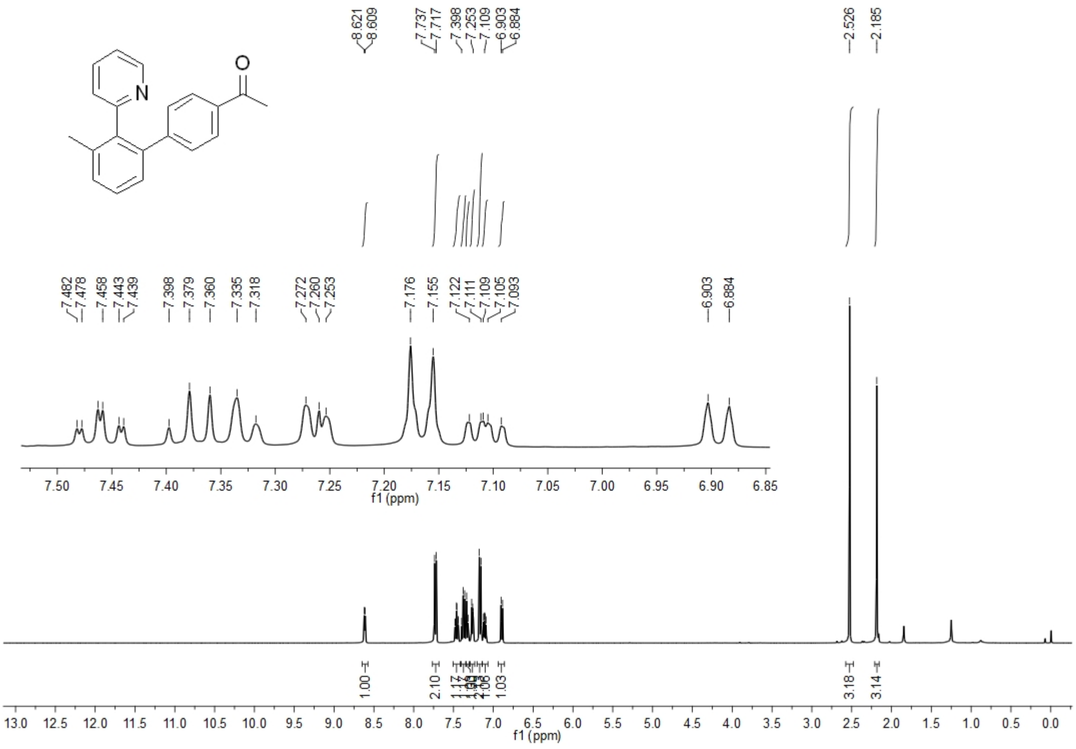
8.621
8.609
7.737
7.717
7.398
7.253
7.109
6.903
6.884
2.526
2.185
7.482
7.478
7.458
7.443
7.439
7.398
7.379
7.360
7.335
7.318
7.272
7.260
7.253
7.176
7.155
7.122
7.111
7.109
7.105
7.093
6.903
6.884
N
O
f1 (ppm)
1.00
2.10
1.03
3.18
3.14
f1 (ppm)

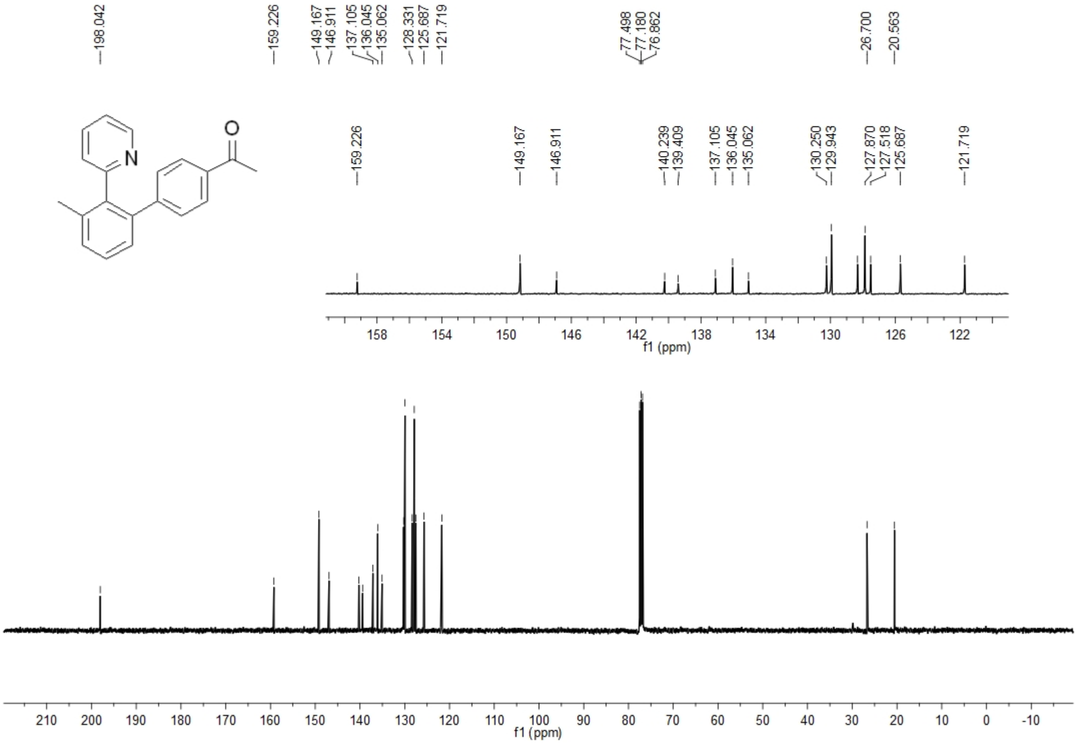
198.042
159.226
149.167
146.911
137.105
136.045
135.062
128.331
125.687
121.719
77.498
77.180
76.862
26.700
20.563
159.226
149.167
146.911
140.239
139.409
137.105
136.045
135.062
130.250
129.943
127.870
127.518
125.687
121.719
N
O
f1 (ppm)
f1 (ppm)

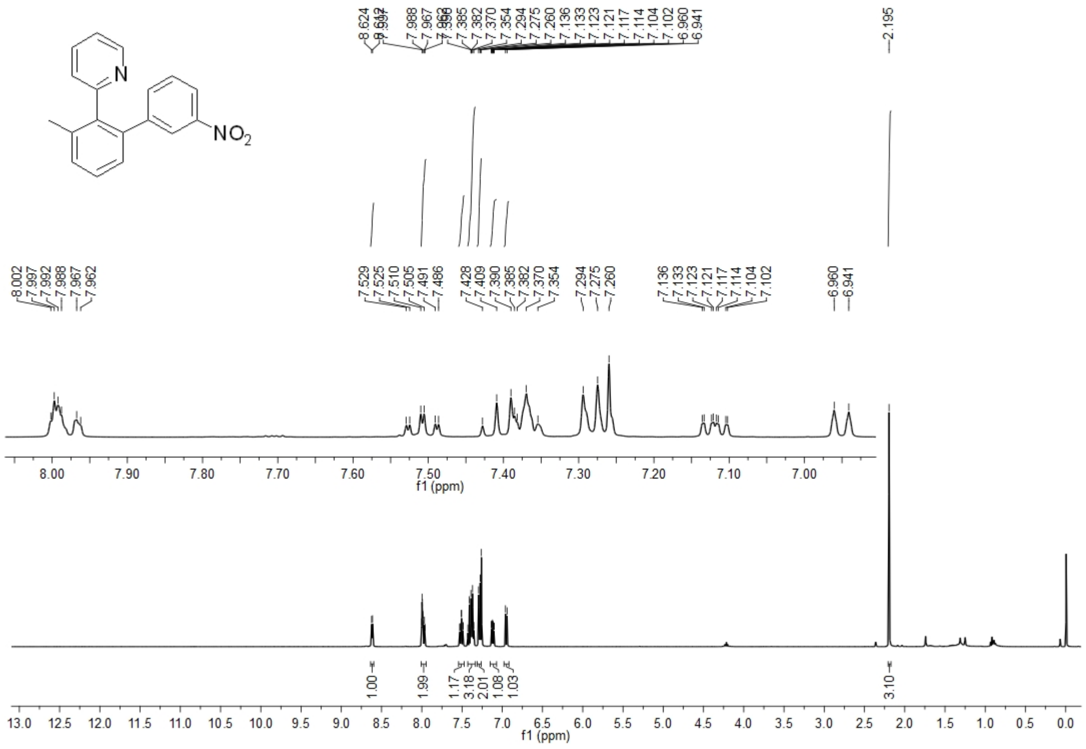

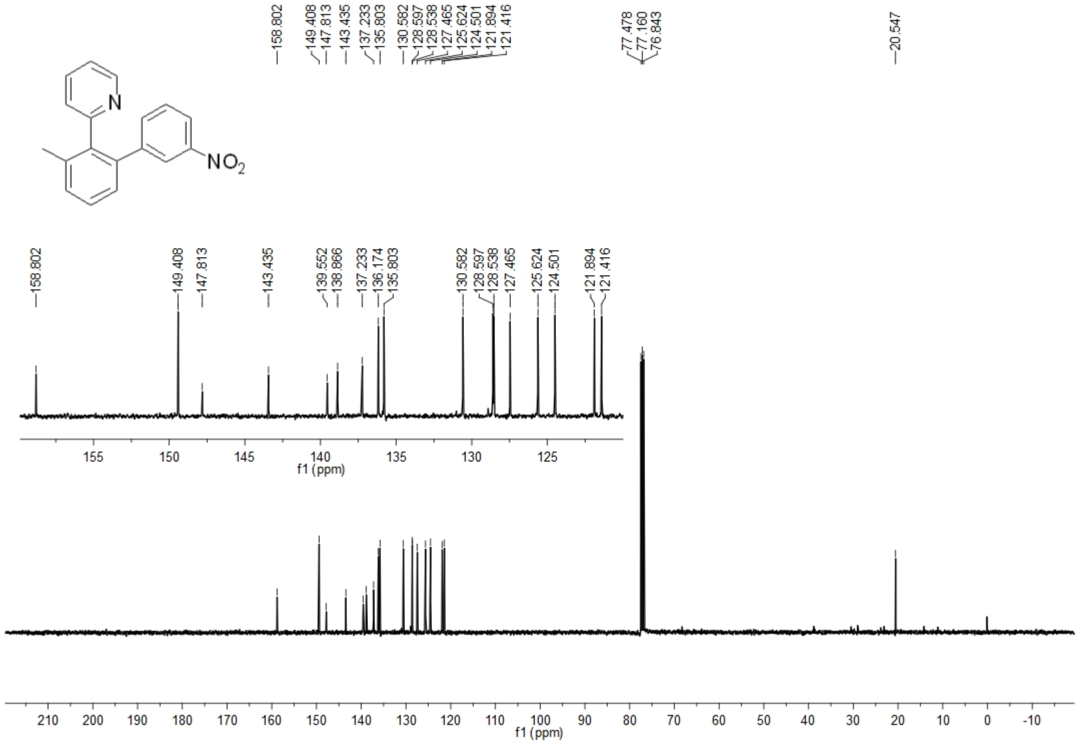

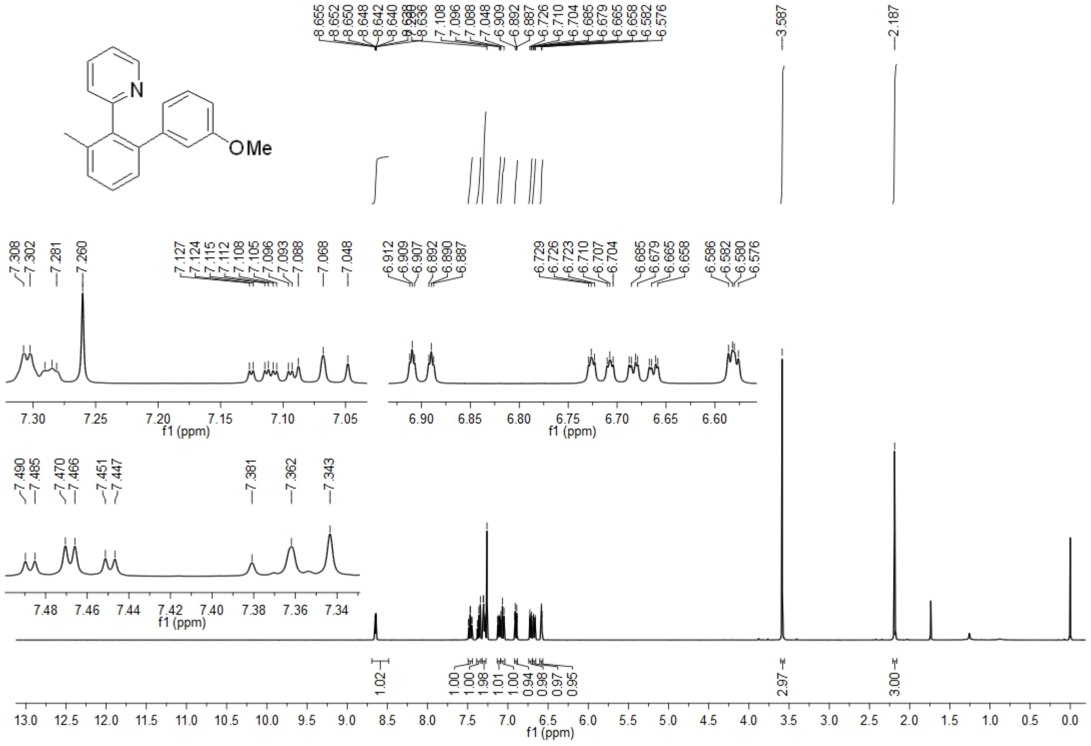
OMe
N
8.655 8.652 8.650 8.648 8.642 8.640 8.638 8.636 7.108 7.096 7.088 7.068 7.048 6.909 6.892 6.887 6.726 6.710 6.704 6.685 6.679 6.665 6.658 6.582 6.576
3.587
2.187
7.308 7.302 7.281 7.260
7.127 7.124 7.115 7.112 7.108 7.105 7.096 7.093 7.088 7.068 7.048
6.912 6.909 6.907 6.892 6.890 6.887
6.729 6.726 6.723 6.710 6.707 6.704 6.685 6.679 6.665 6.658 6.586 6.582 6.580 6.576
7.490 7.485 7.470 7.466 7.451 7.447 7.381 7.362 7.343
1.02 1.00 1.00 1.98 1.01 1.00 0.94 0.98 0.97 0.95 2.97 3.00
f1 (ppm)

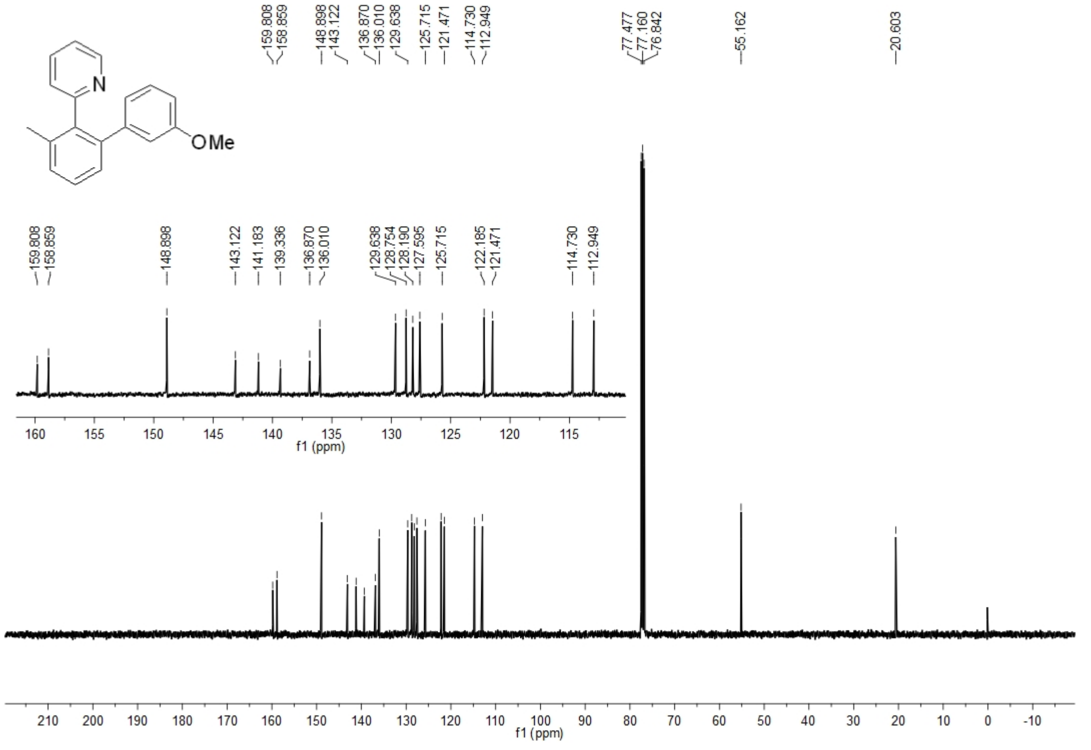
OMe
N
159.808 158.859 148.898 143.122 136.870 136.010 129.638 125.715 121.471 114.730 112.949
77.477 77.160 76.842
55.162
20.603
159.808 158.859 148.898 143.122 141.183 139.336 136.870 136.010 129.638 128.754 128.190 127.595 125.715 122.185 121.471 114.730 112.949
f1 (ppm)

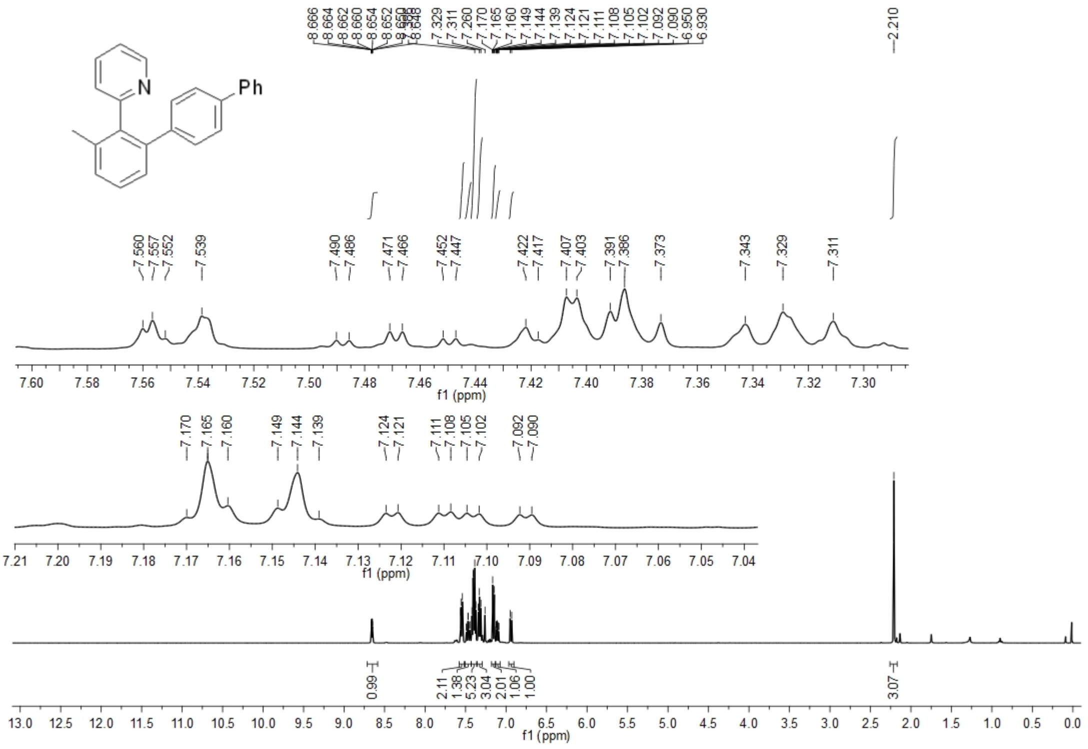

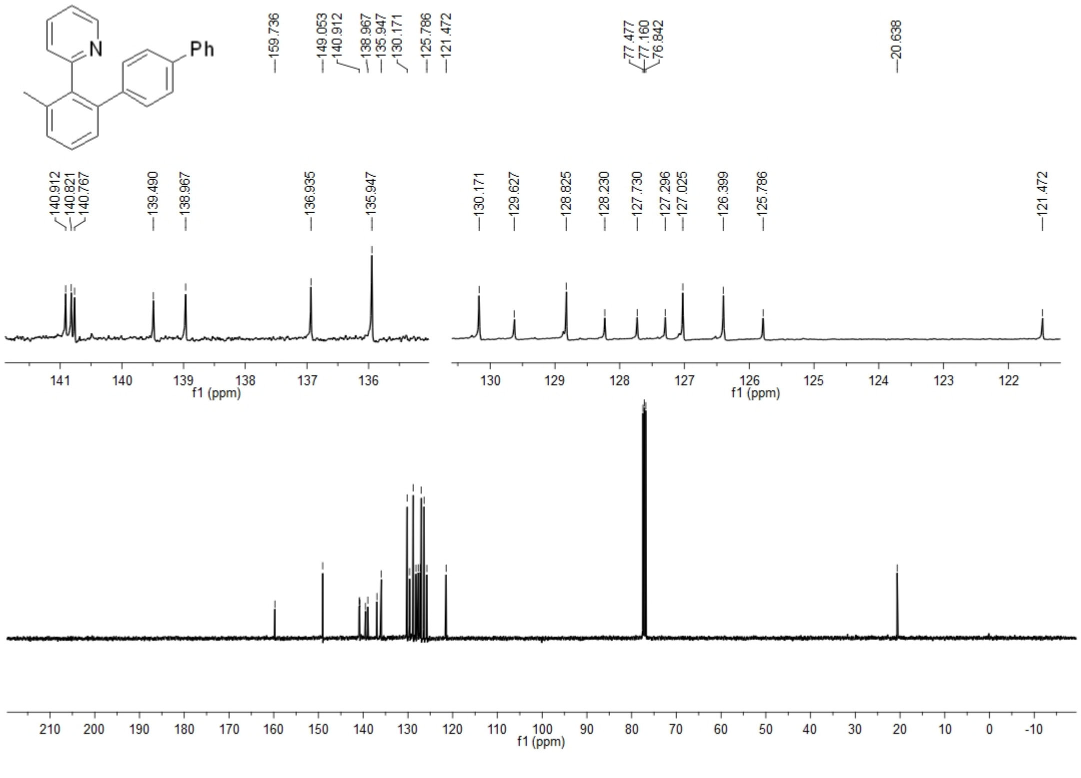

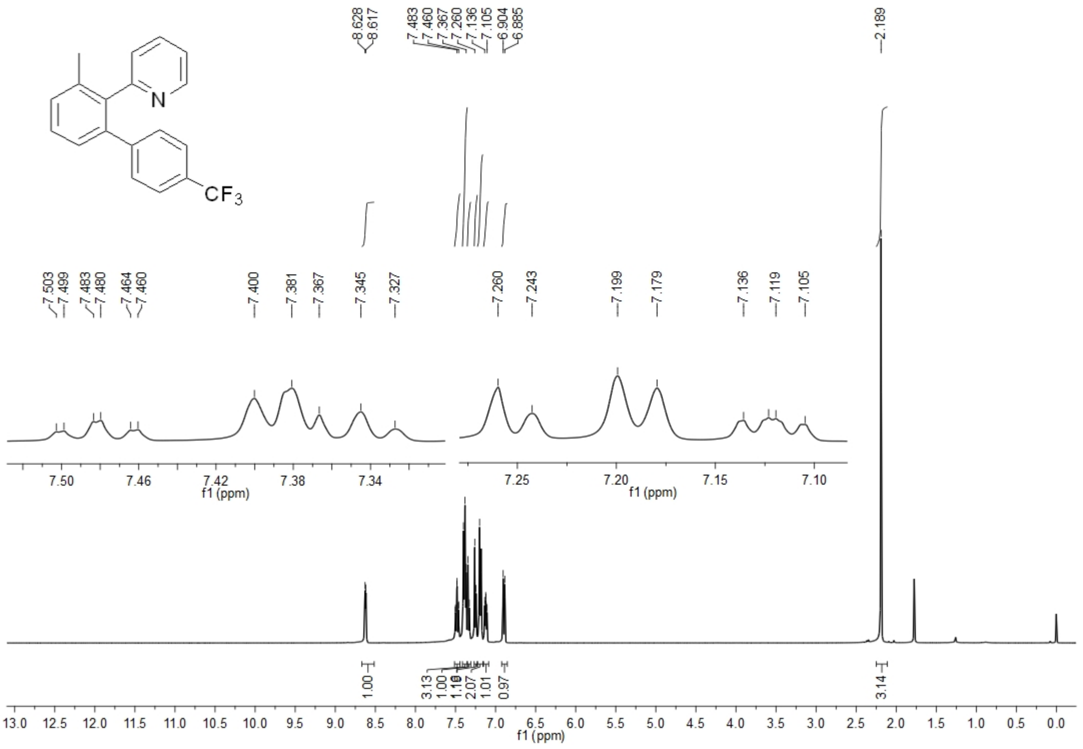
N
CF3
8.628
8.617
7.483
7.460
7.367
7.260
7.136
7.105
6.904
6.885
2.189
7.503
7.499
7.483
7.480
7.464
7.460
7.400
7.381
7.367
7.345
7.327
7.260
7.243
7.199
7.179
7.136
7.119
7.105
f1 (ppm)
1.00
3.13
1.00
2.07
1.01
0.97
3.14
f1 (ppm)

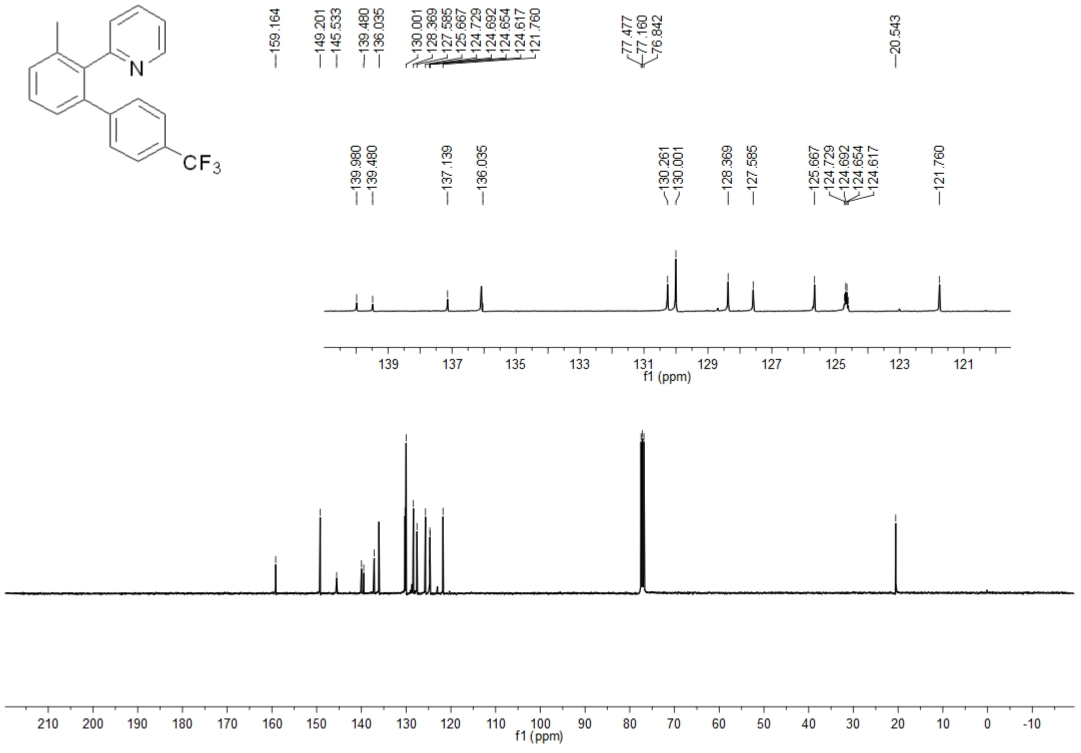
N
CF3
159.164
149.201
145.533
139.480
136.035
130.001
128.369
127.585
125.667
124.729
124.692
124.654
124.617
121.760
77.477
77.160
76.842
20.543
139.980
139.480
137.139
136.035
130.261
130.001
128.369
127.585
125.667
124.729
124.692
124.654
124.617
121.760
f1 (ppm)
f1 (ppm)

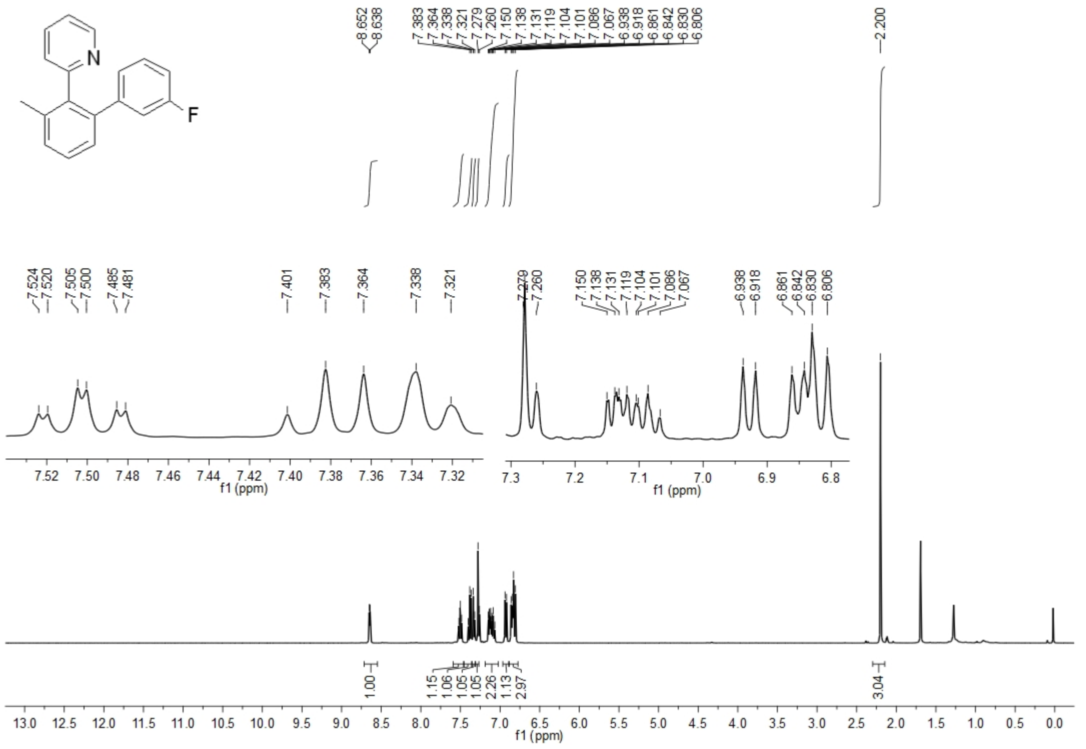

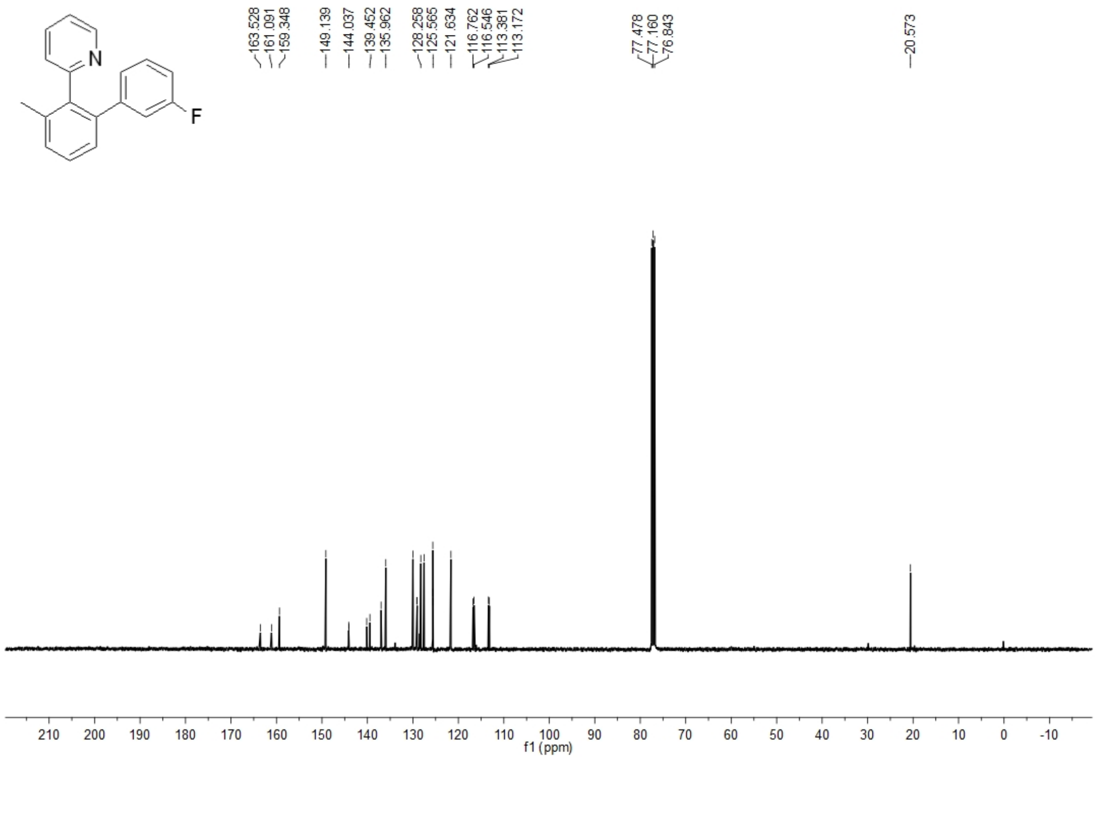

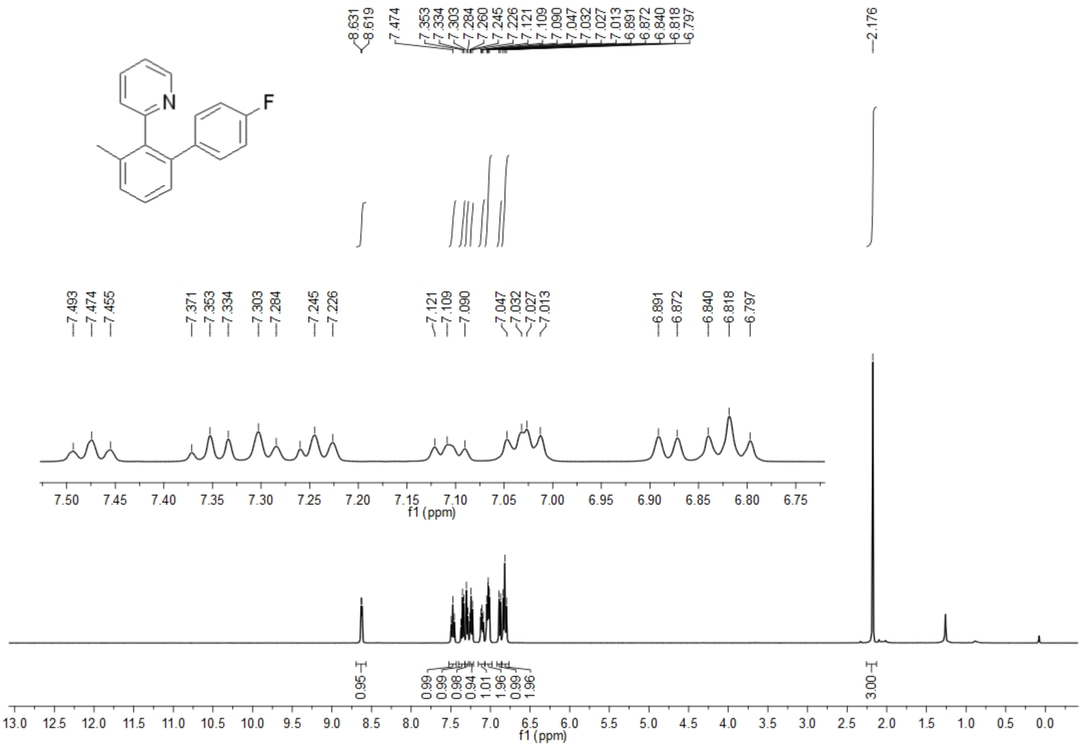

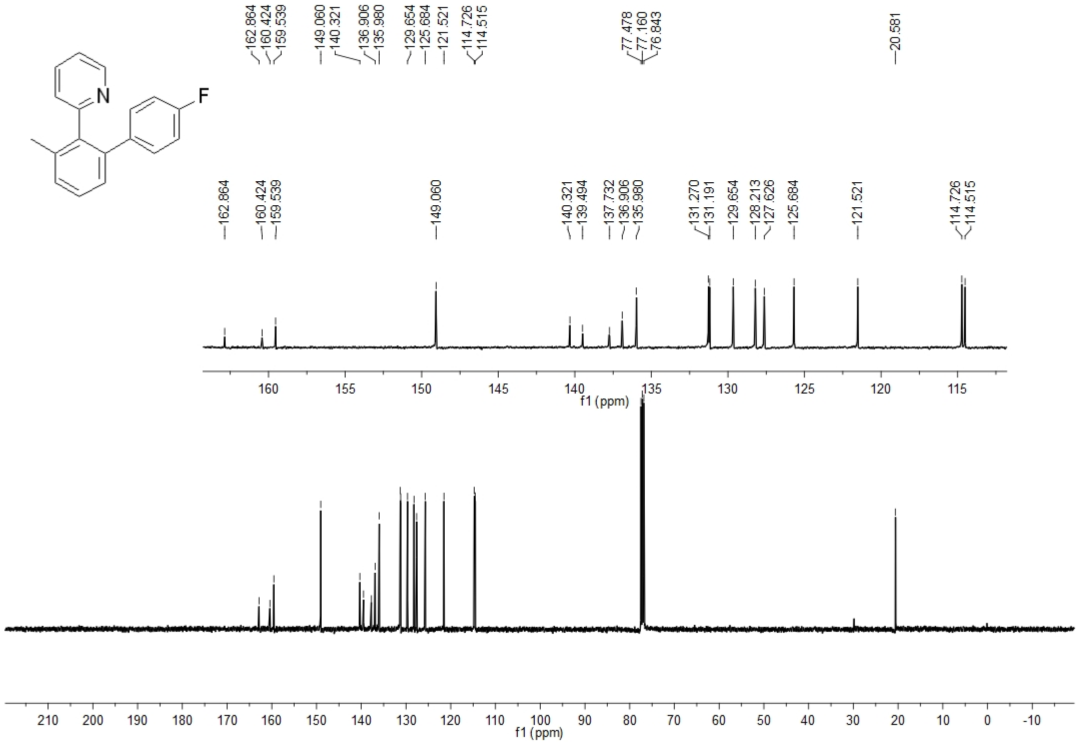

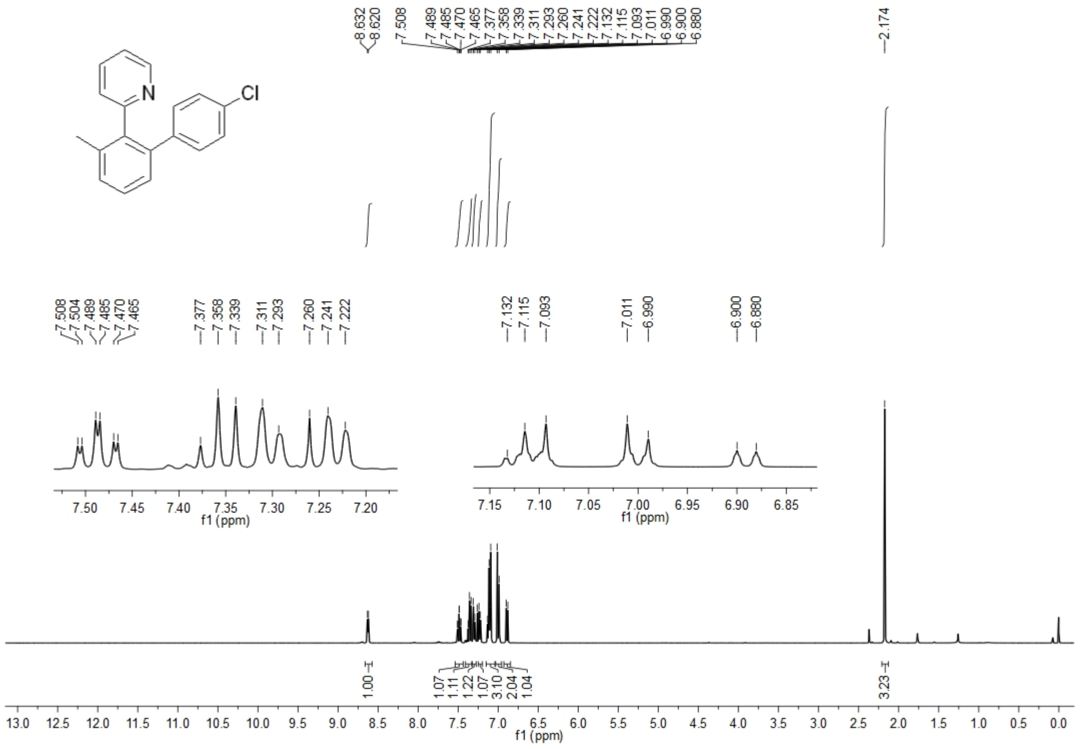

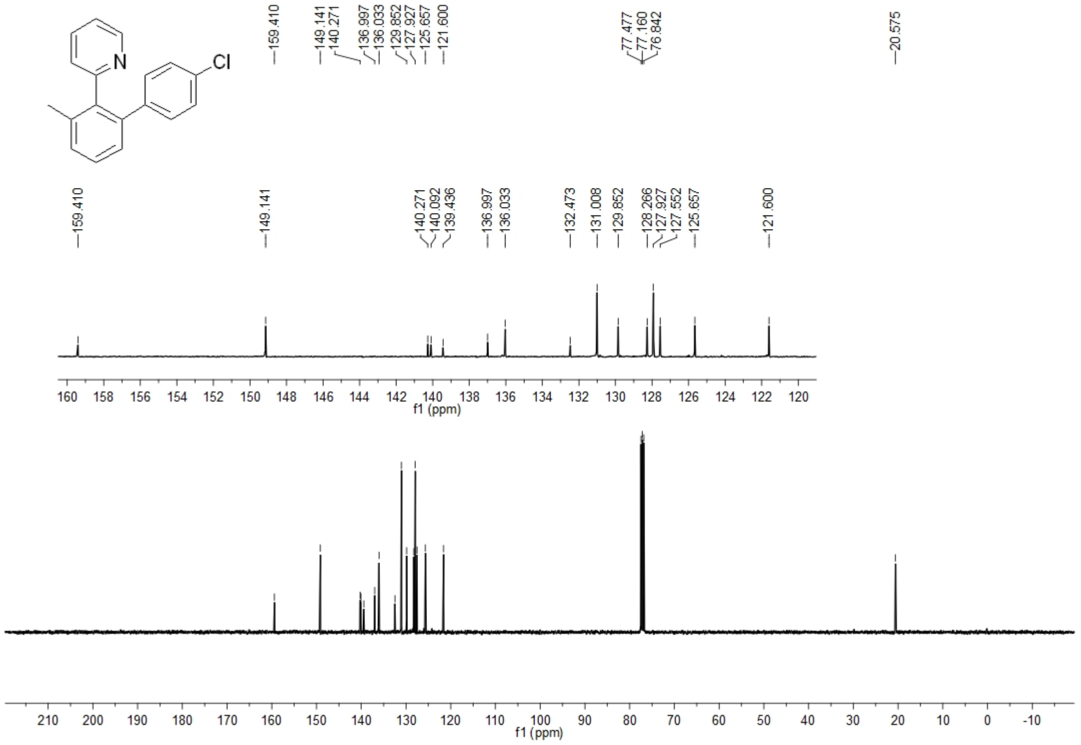

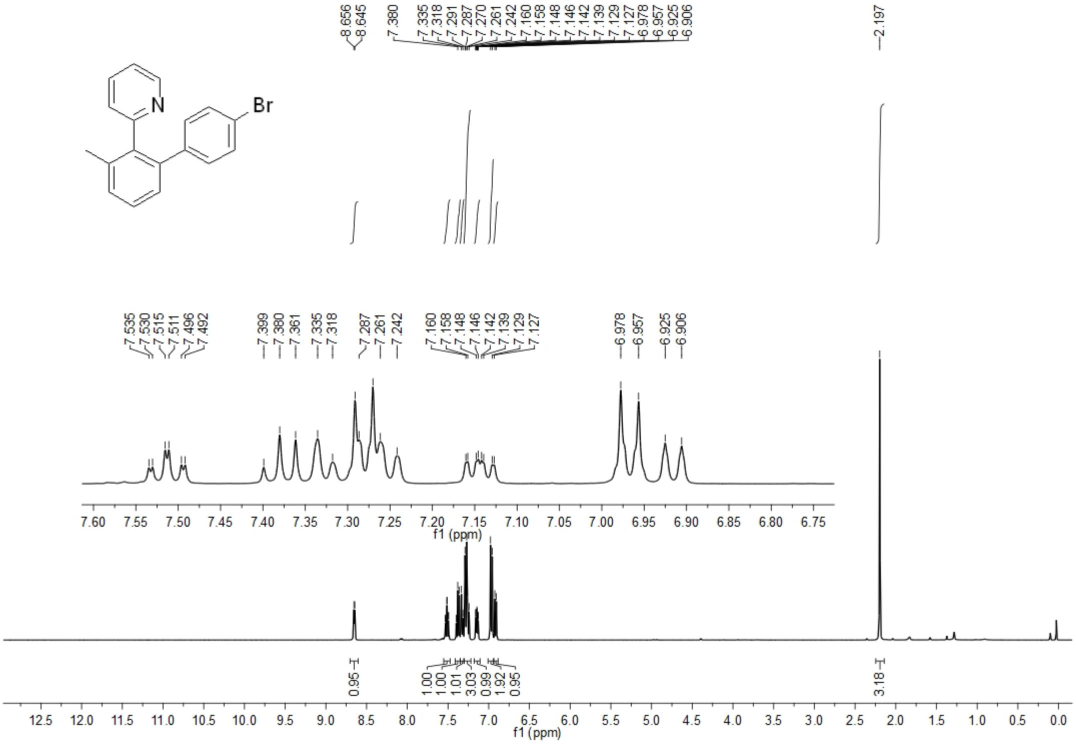
Br
N
8.656
8.645
7.380
7.335
7.318
7.291
7.287
7.270
7.261
7.242
7.160
7.158
7.148
7.146
7.142
7.139
7.129
7.127
6.978
6.957
6.925
6.906
2.197
f1 (ppm)
0.95
1.00
1.00
1.01
3.03
0.99
1.92
0.95
3.18

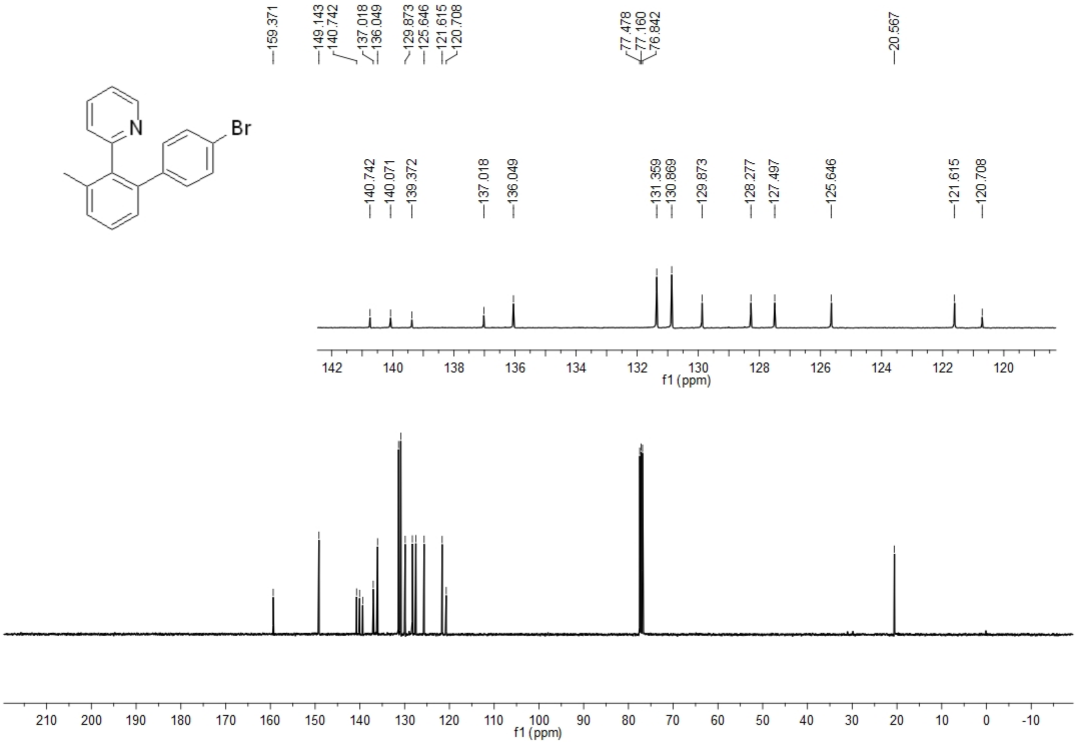
Br
N
159.371
149.143
140.742
137.018
136.049
129.873
125.646
121.615
120.708
77.478
77.160
76.842
20.567
140.742
140.071
139.372
137.018
136.049
131.359
130.869
129.873
128.277
127.497
125.646
121.615
120.708
f1 (ppm)

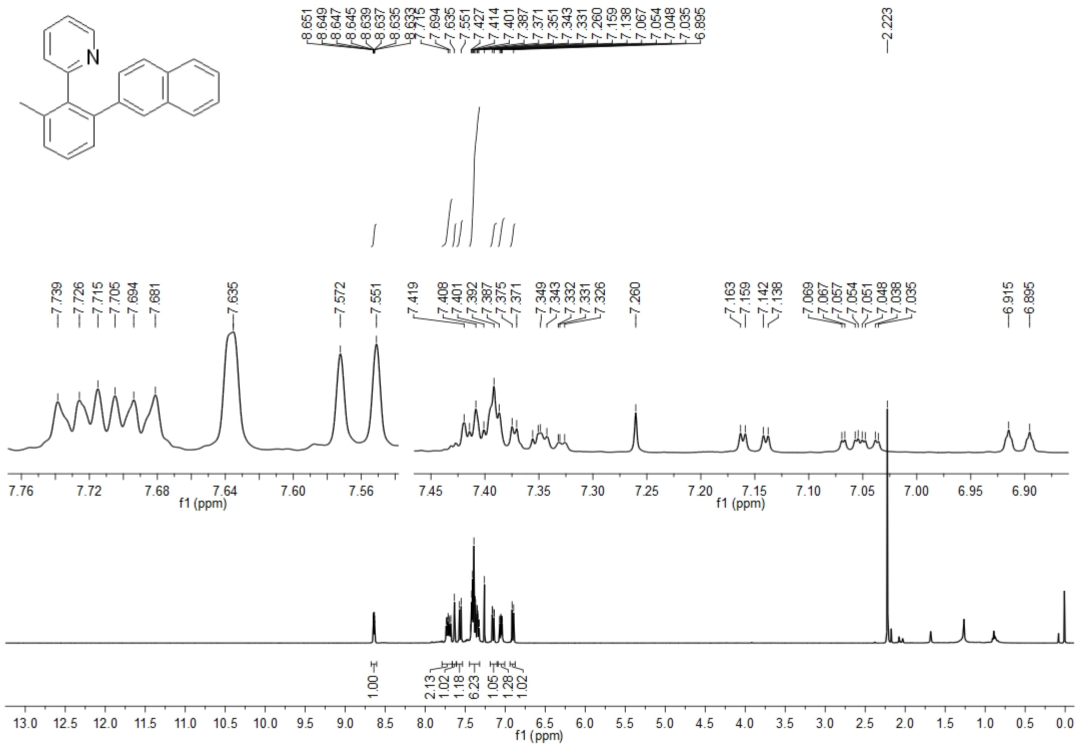

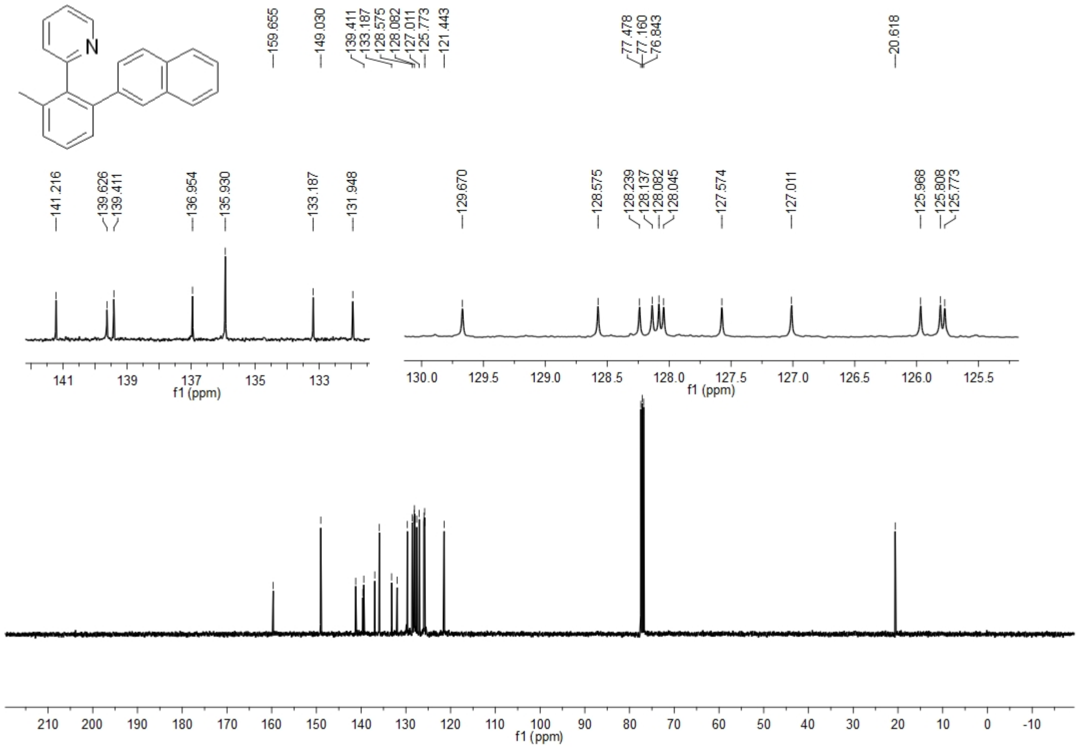

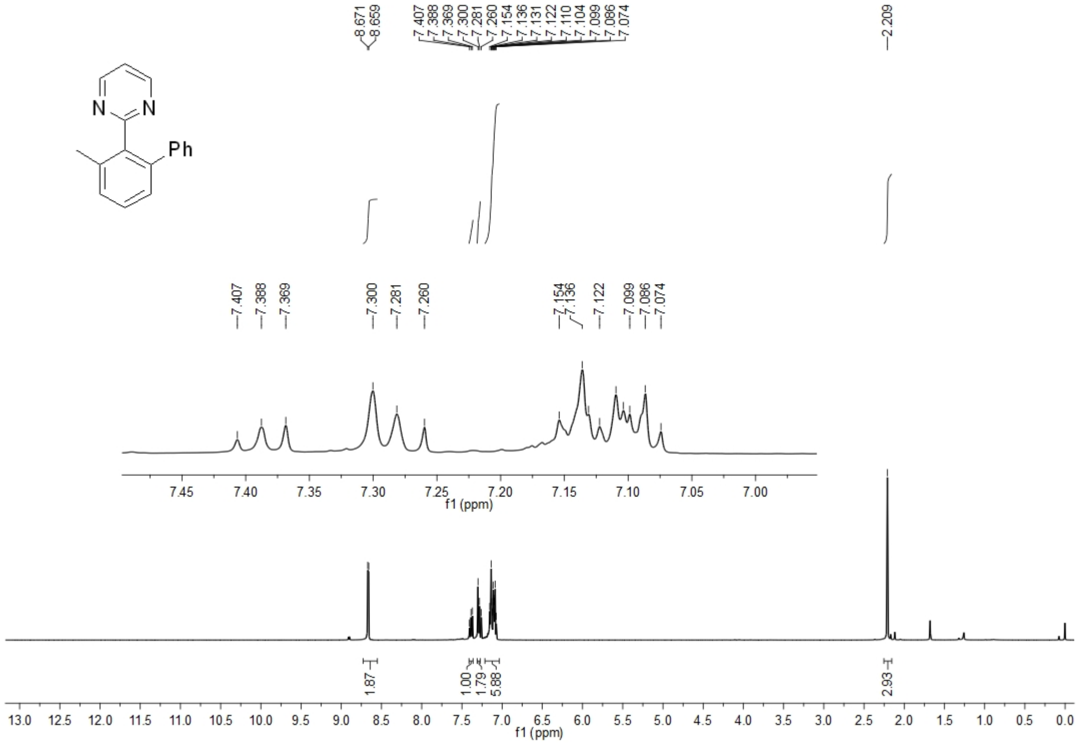
N
N
Ph
8.671
8.659
7.407
7.388
7.369
7.300
7.281
7.260
7.154
7.136
7.131
7.122
7.110
7.104
7.099
7.086
7.074
2.209
1.87
1.00
1.79
5.88
2.93
f1 (ppm)

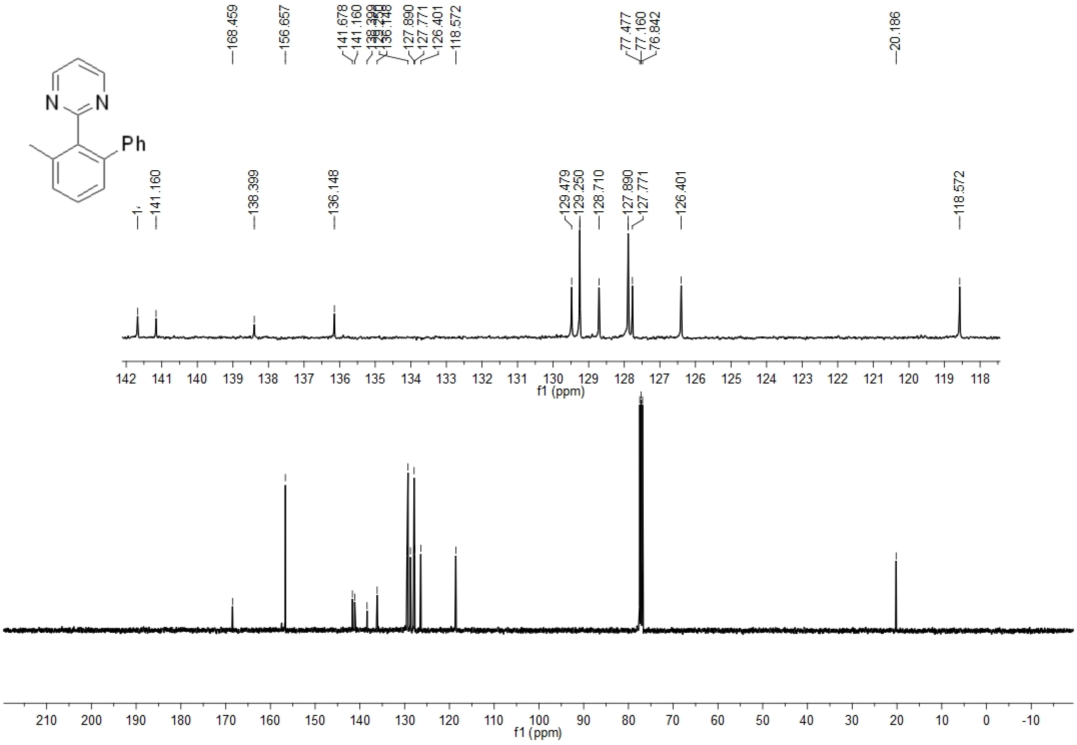
N
N
Ph
168.459
156.657
141.678
141.160
136.148
127.890
127.771
126.401
118.572
77.477
77.160
76.842
20.186
138.399
129.479
129.250
128.710
f1 (ppm)

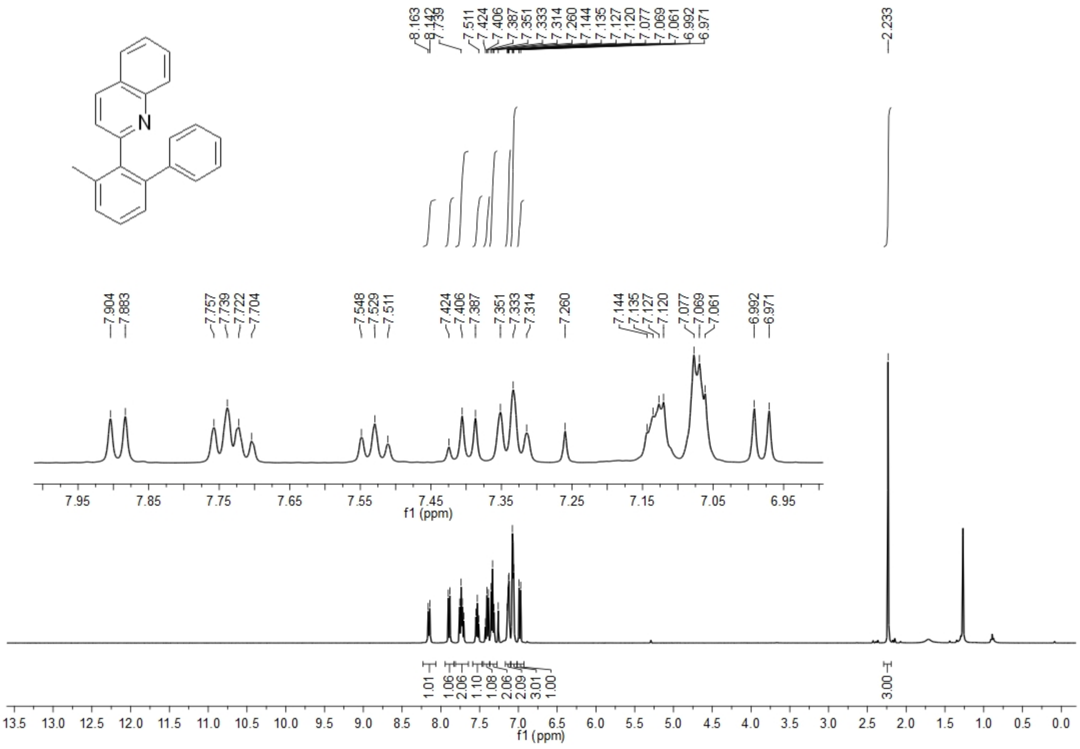

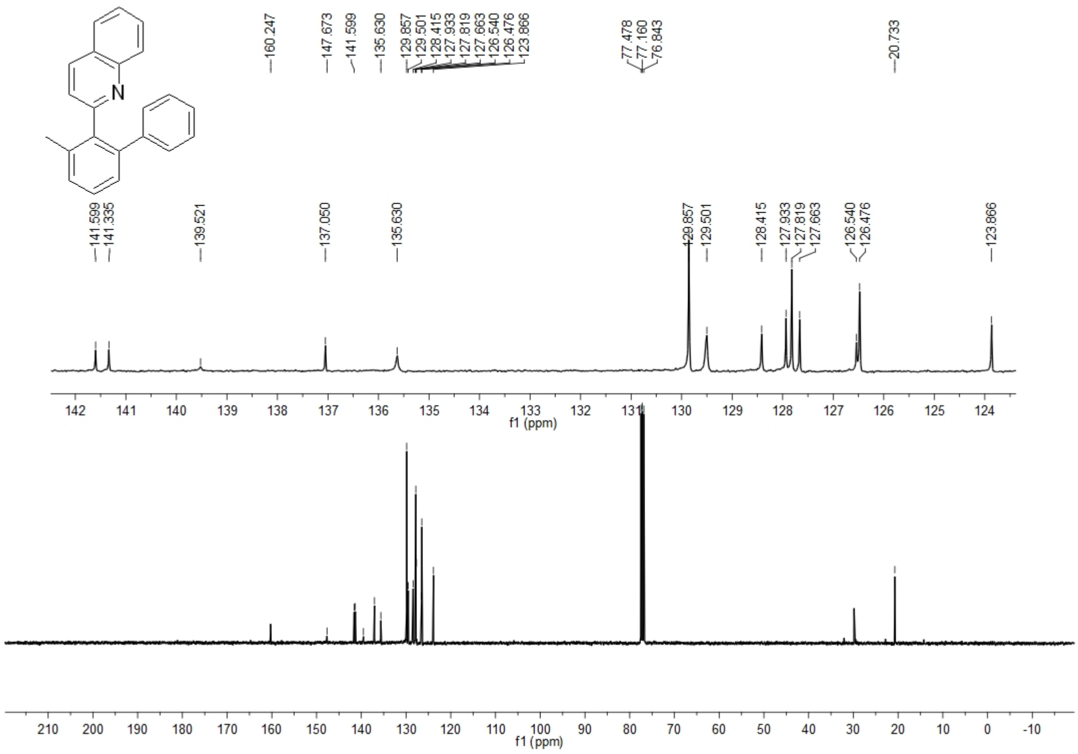

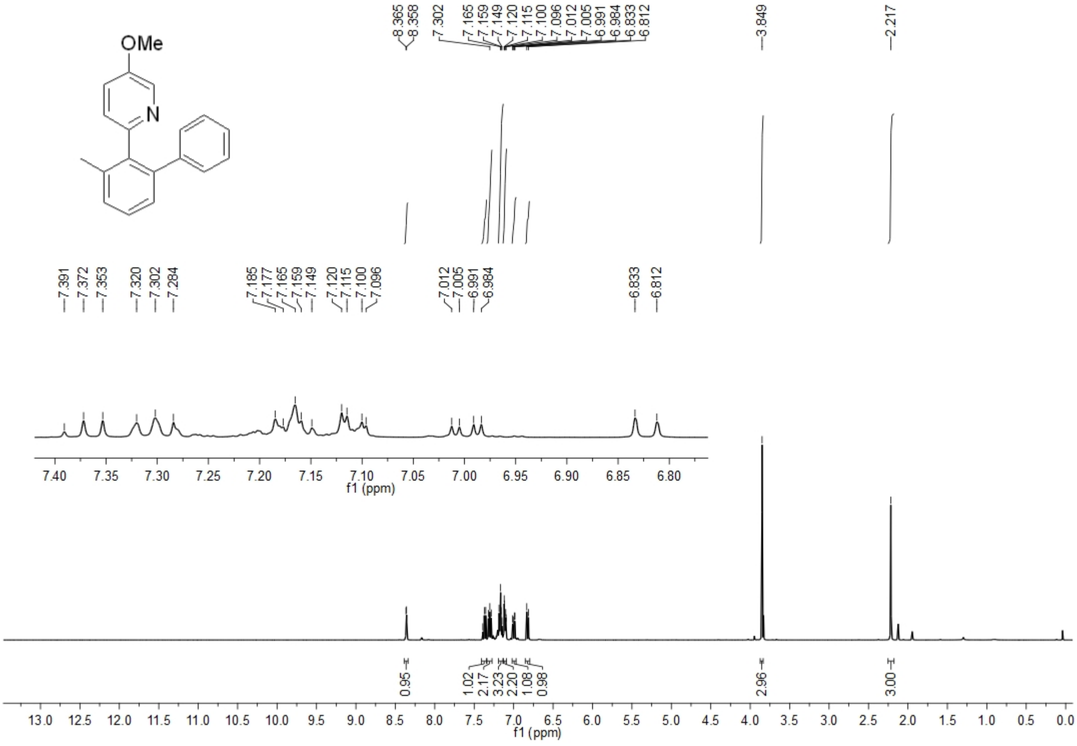

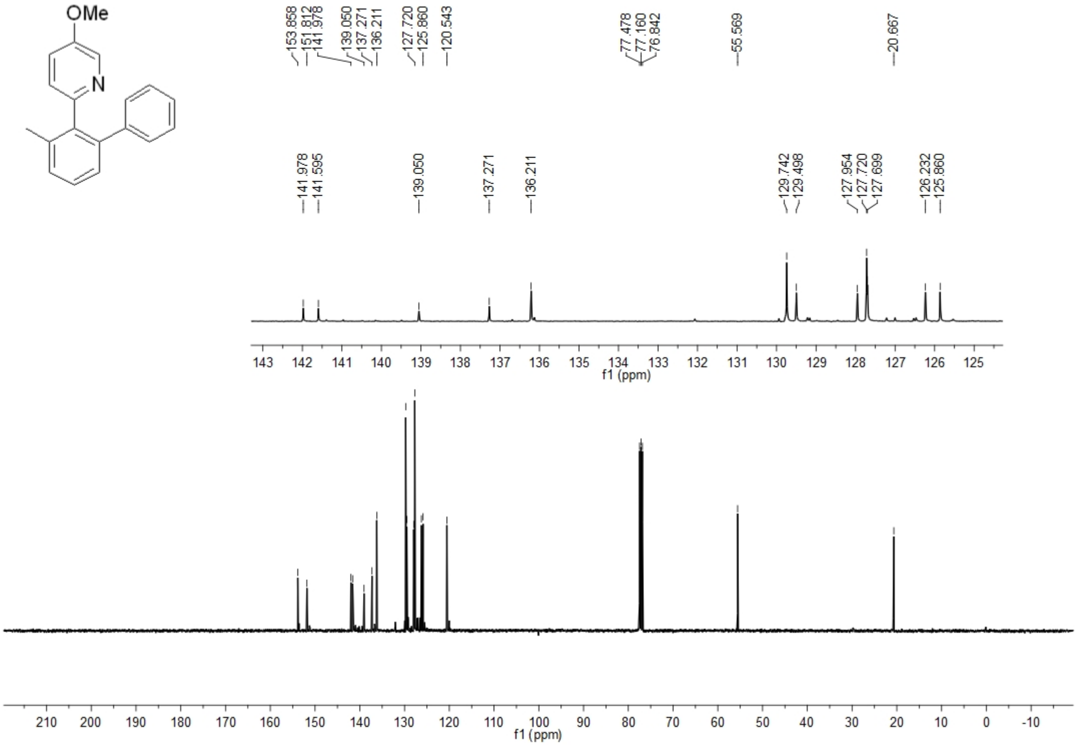

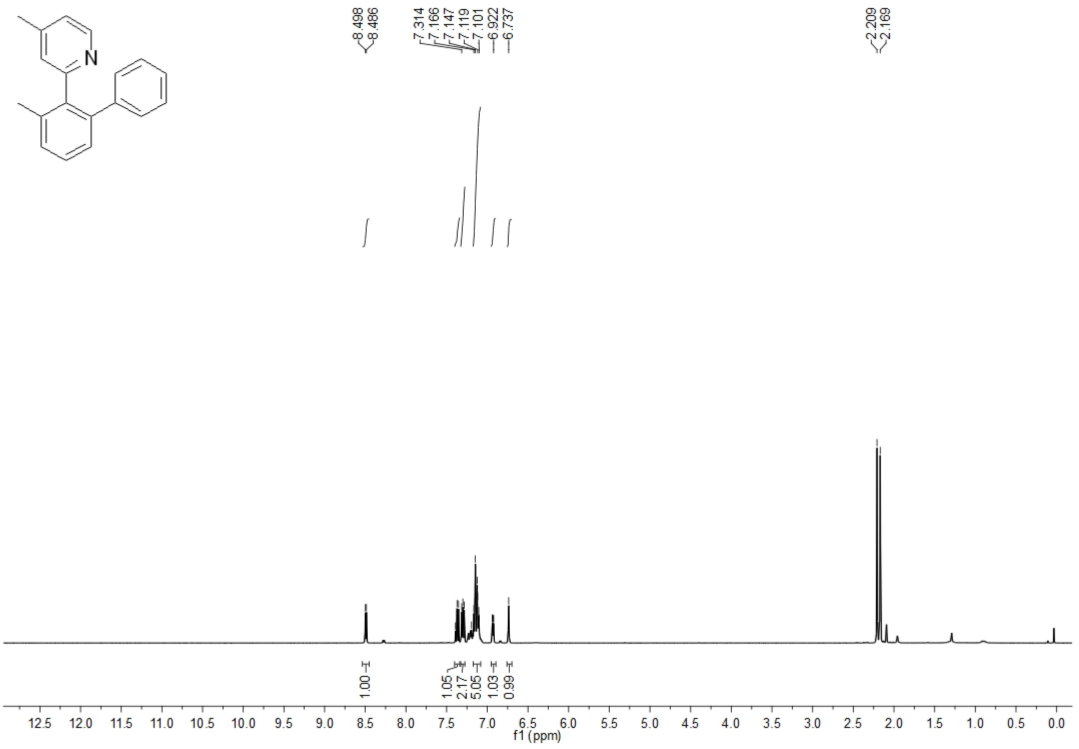
N
8.498
8.486
7.314
7.166
7.147
7.119
7.101
6.922
6.737
2.209
2.169
1.00
1.05
2.17
5.05
1.03
0.99
12.5 12.0 11.5 11.0 10.5 10.0 9.5 9.0 8.5 8.0 7.5 7.0 6.5 6.0 5.5 5.0 4.5 4.0 3.5 3.0 2.5 2.0 1.5 1.0 0.5 0.0
f1 (ppm)

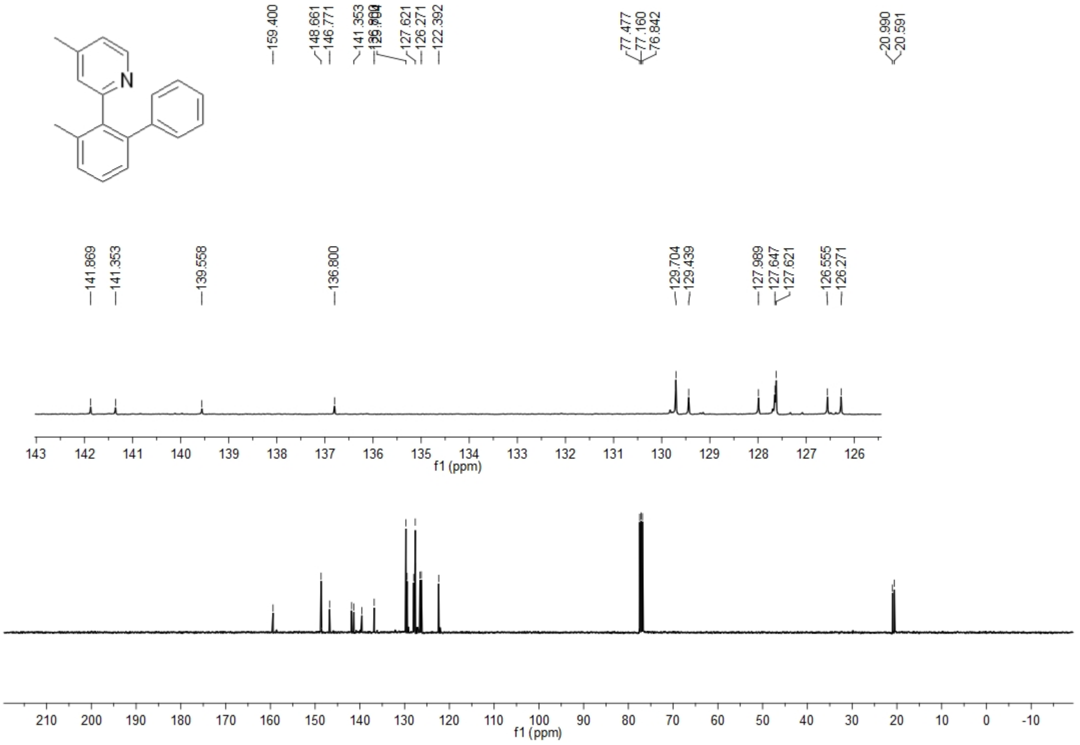
N
159.400
148.661
146.771
141.353
127.621
126.271
122.392
77.477
77.160
76.842
20.990
20.591
141.869
141.353
139.558
136.800
129.704
129.439
127.989
127.647
127.621
126.555
126.271
143 142 141 140 139 138 137 136 135 134 133 132 131 130 129 128 127 126
f1 (ppm)
210 200 190 180 170 160 150 140 130 120 110 100 90 80 70 60 50 40 30 20 10 0 -10
f1 (ppm)

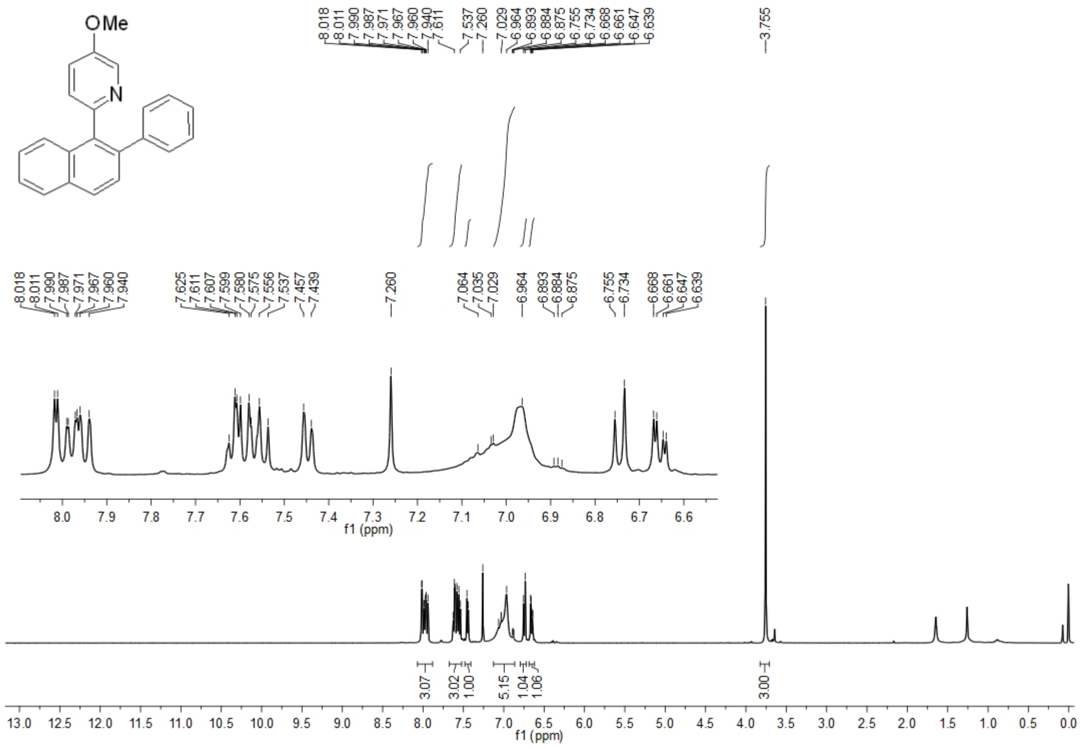

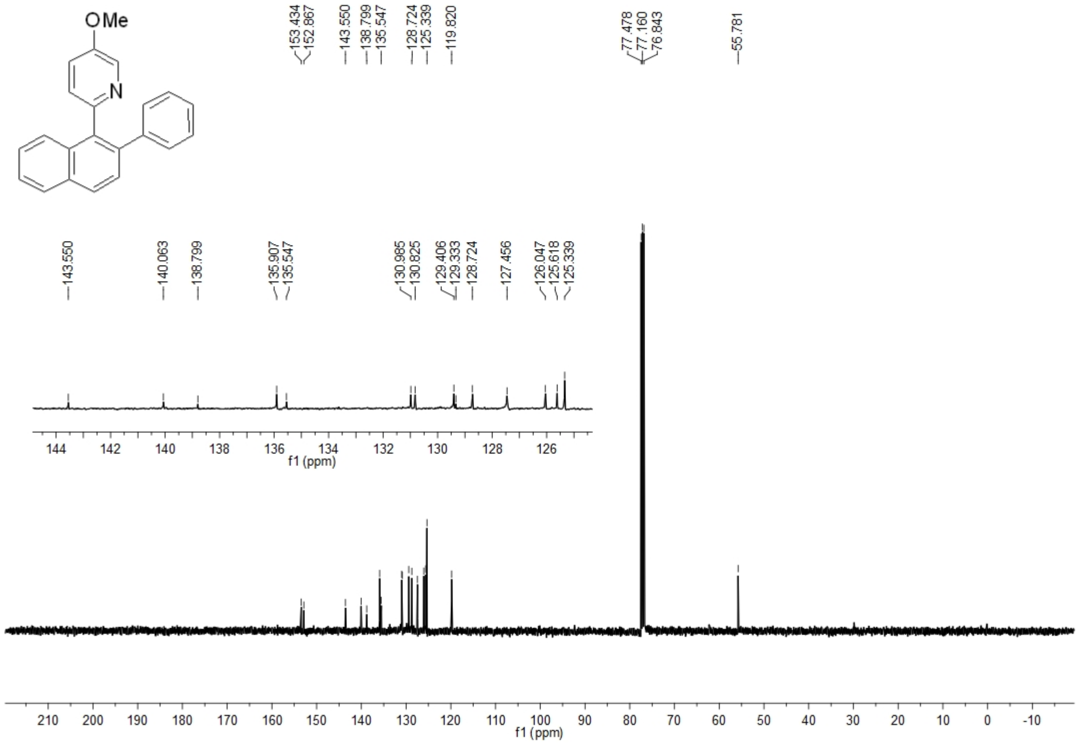

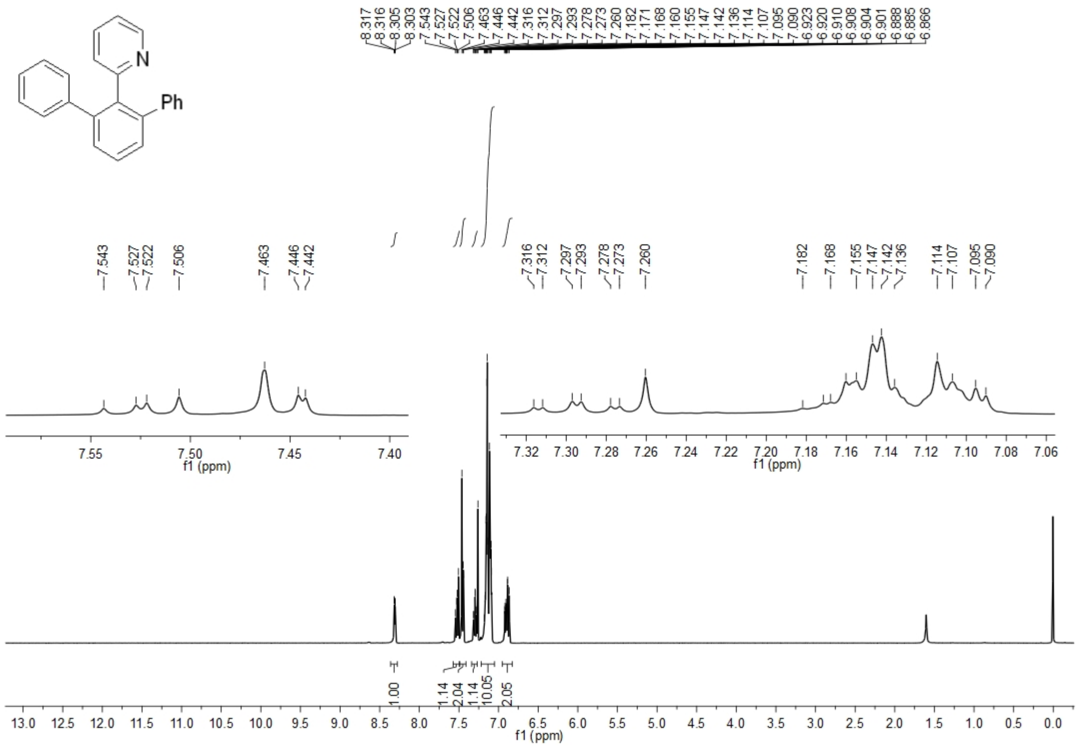

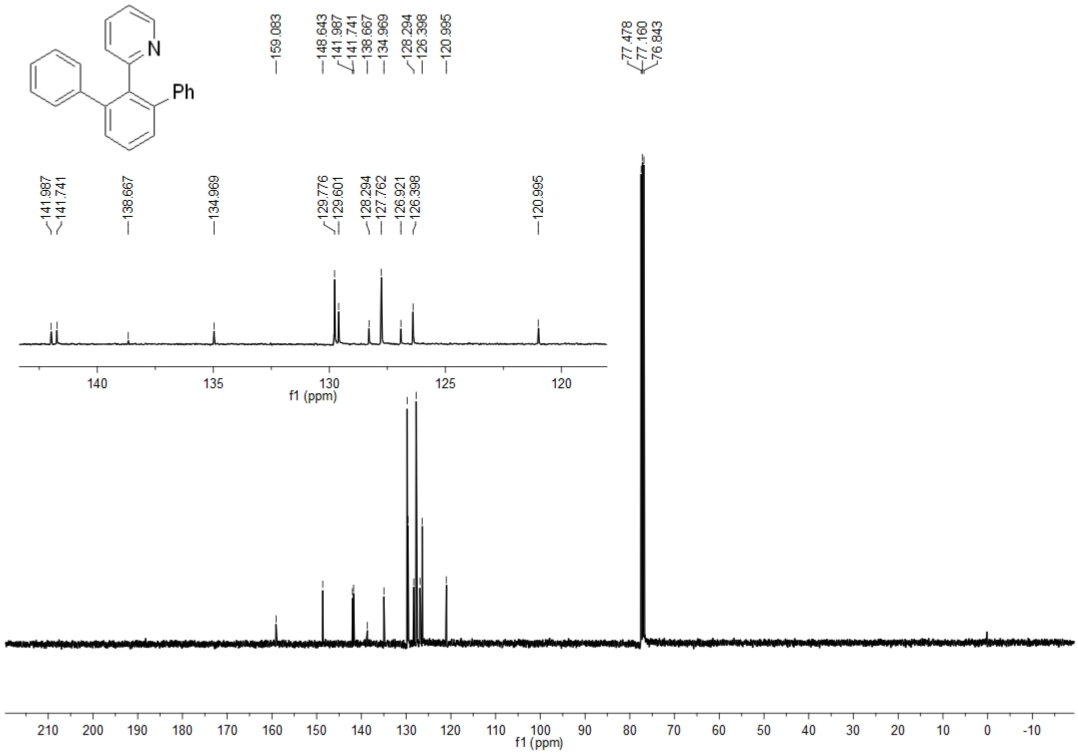

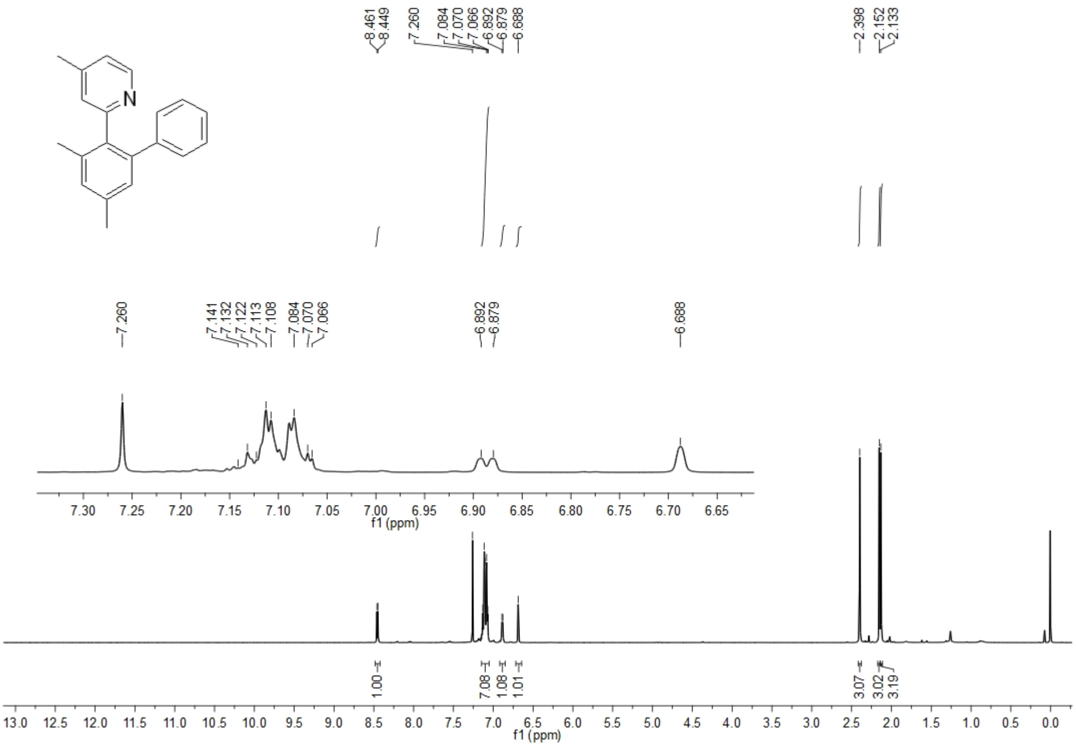
8.461
8.449
7.260
7.084
7.070
7.066
6.892
6.879
6.688
2.398
2.152
2.133
7.141
7.132
7.122
7.113
7.108
1.00
7.08
1.08
1.01
3.07
3.02
3.19
f1 (ppm)

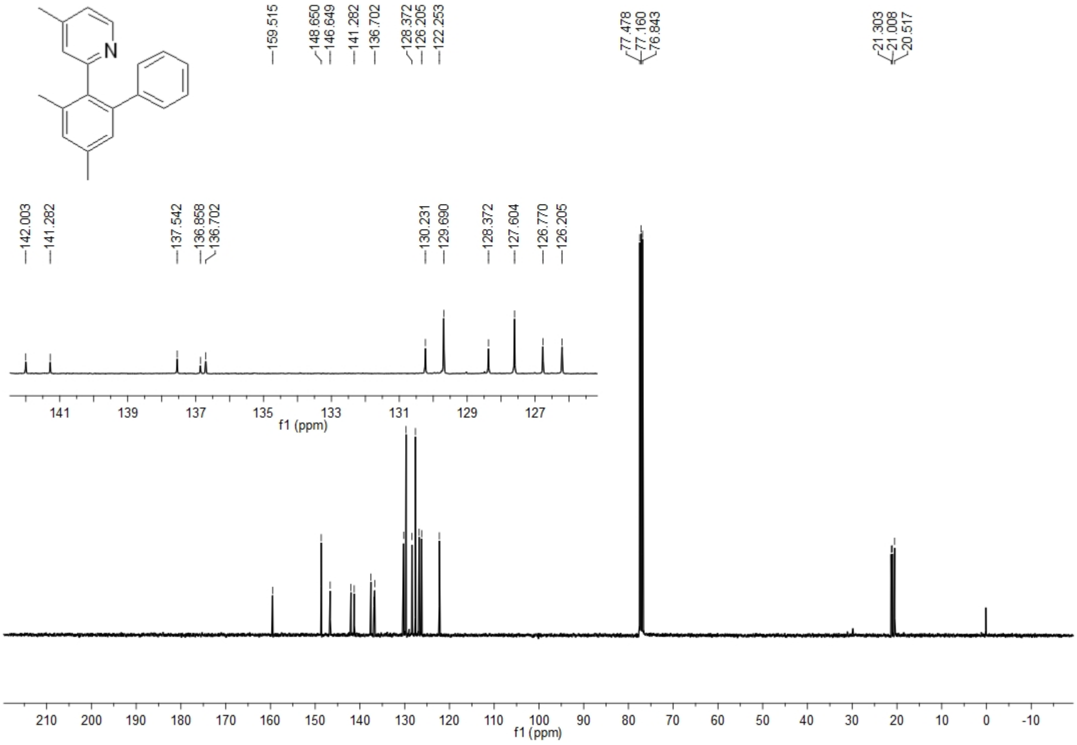
159.515
148.650
146.649
141.282
136.702
128.372
126.205
122.253
77.478
77.160
76.843
21.303
21.008
20.517
142.003
137.542
136.858
130.231
129.690
127.604
126.770
f1 (ppm)

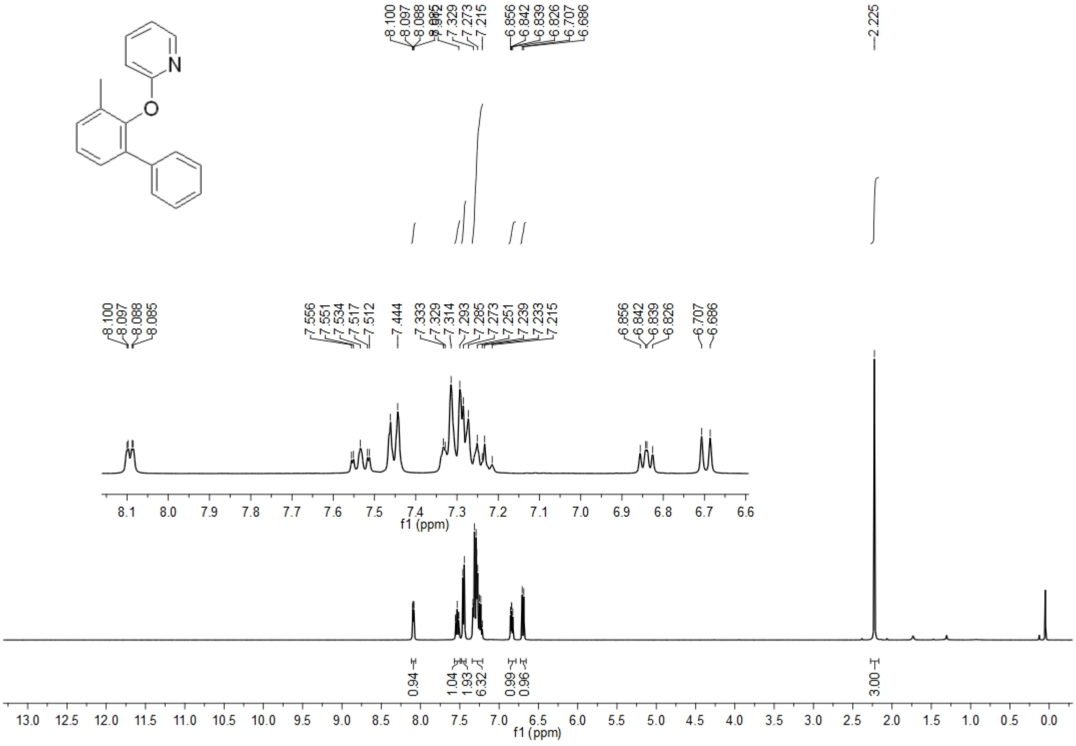
N
O
8.100
8.097
8.088
7.329
7.273
7.215
6.856
6.842
6.839
6.826
6.707
6.686
2.225
8.100
8.097
8.088
8.085
7.556
7.551
7.534
7.517
7.512
7.444
7.333
7.329
7.314
7.293
7.285
7.273
7.251
7.239
7.233
7.215
6.856
6.842
6.839
6.826
6.707
6.686
f1 (ppm)
0.94
1.04
1.93
6.32
0.99
0.96
3.00
f1 (ppm)

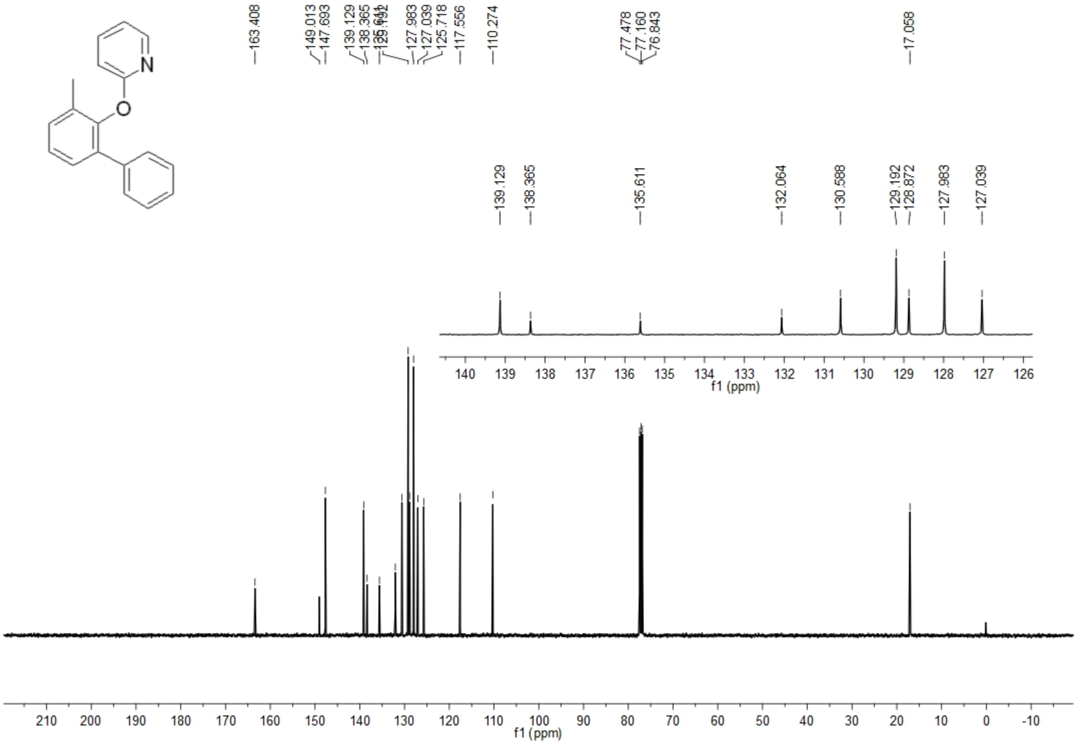
N
O
163.408
149.013
147.693
139.129
138.365
127.983
127.039
125.718
117.556
110.274
77.478
77.160
76.843
17.058
139.129
138.365
135.611
132.064
130.588
129.192
128.872
127.983
127.039
f1 (ppm)
f1 (ppm)

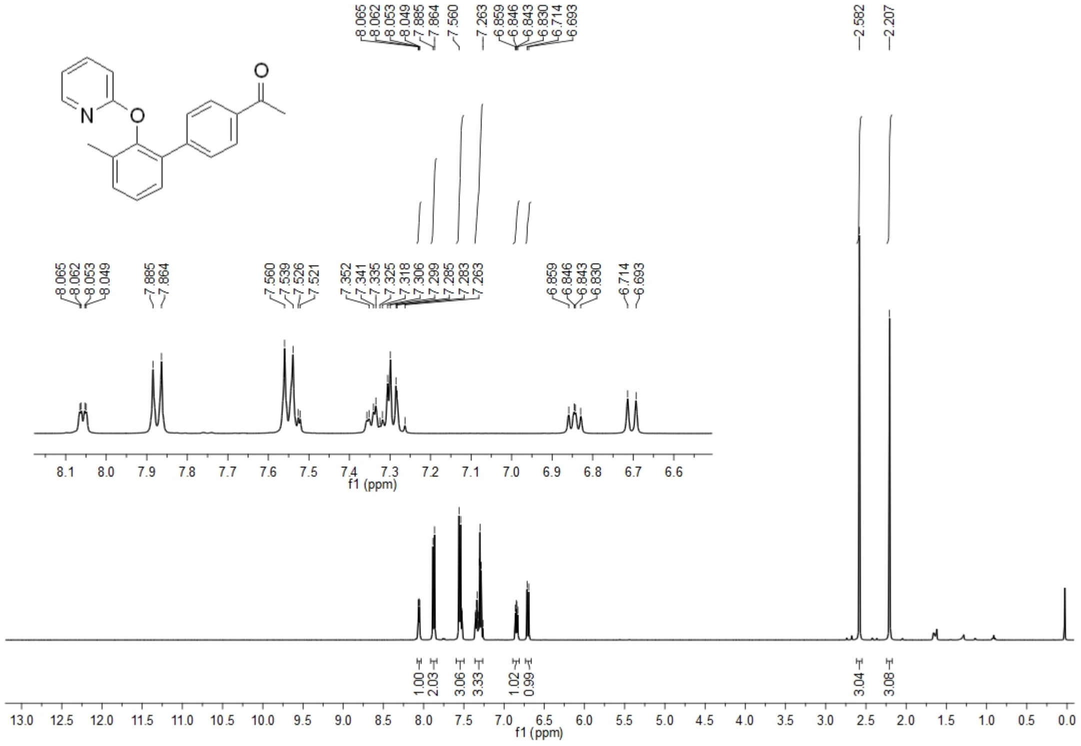

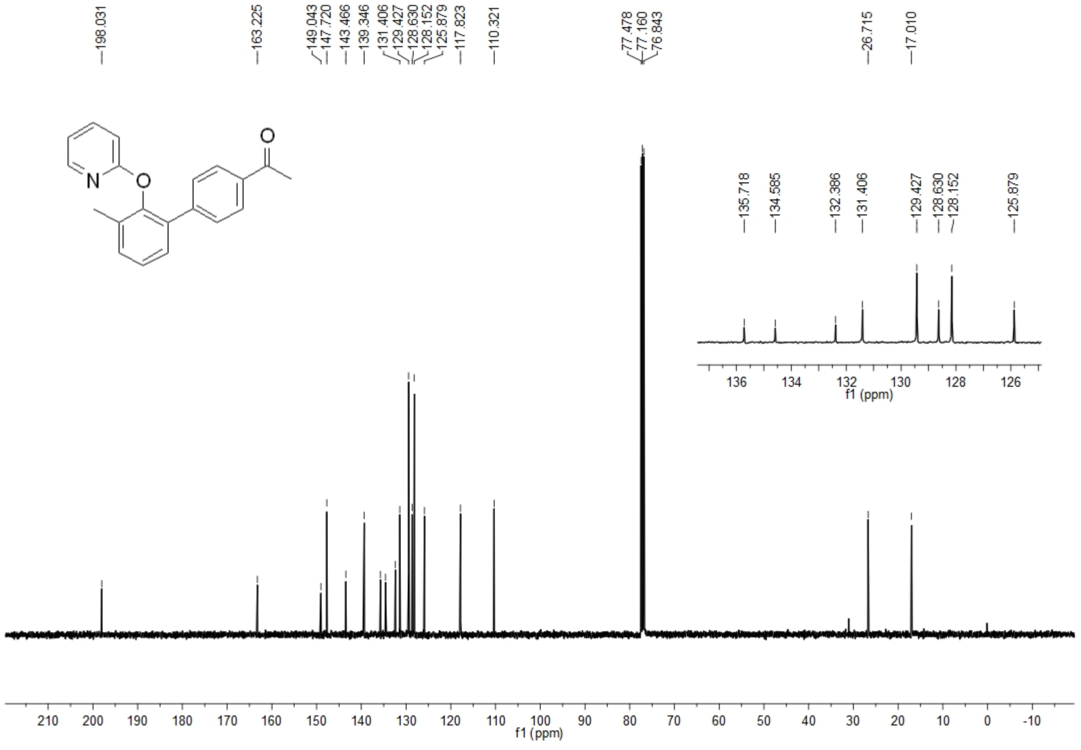

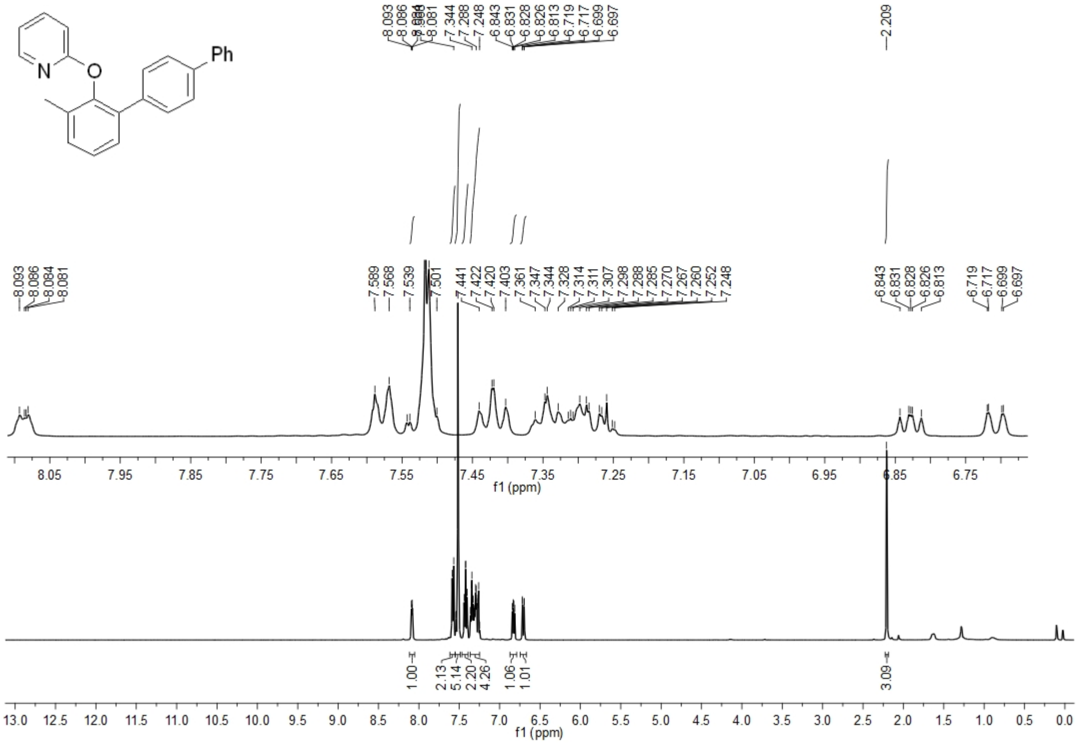

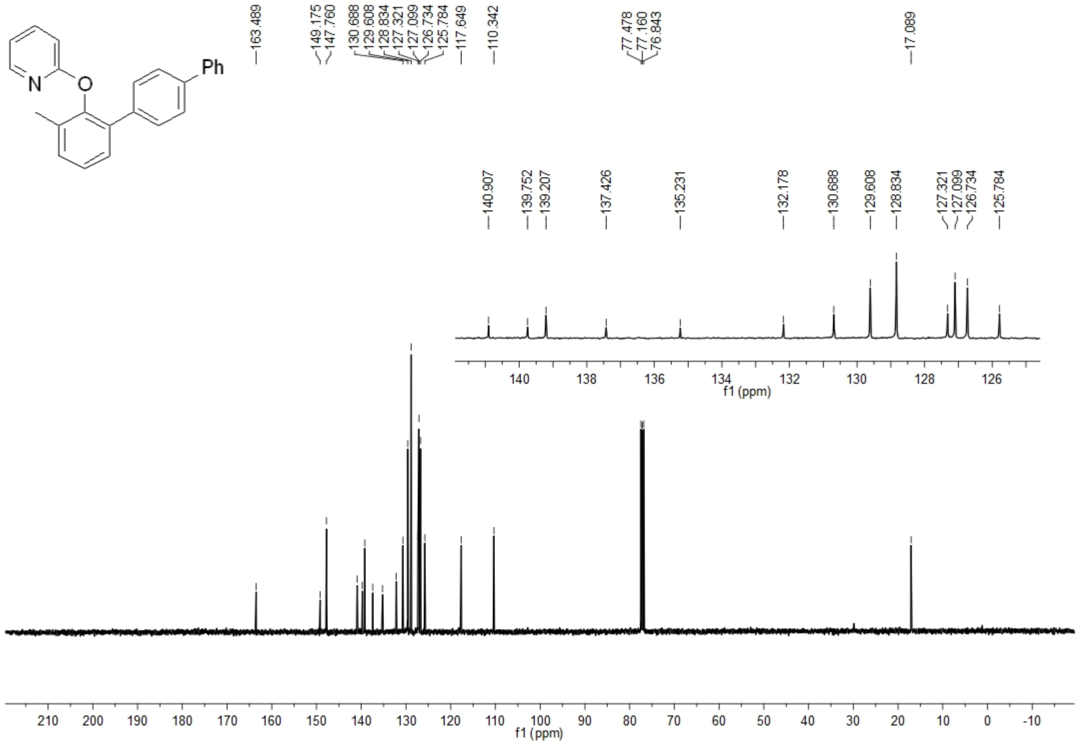

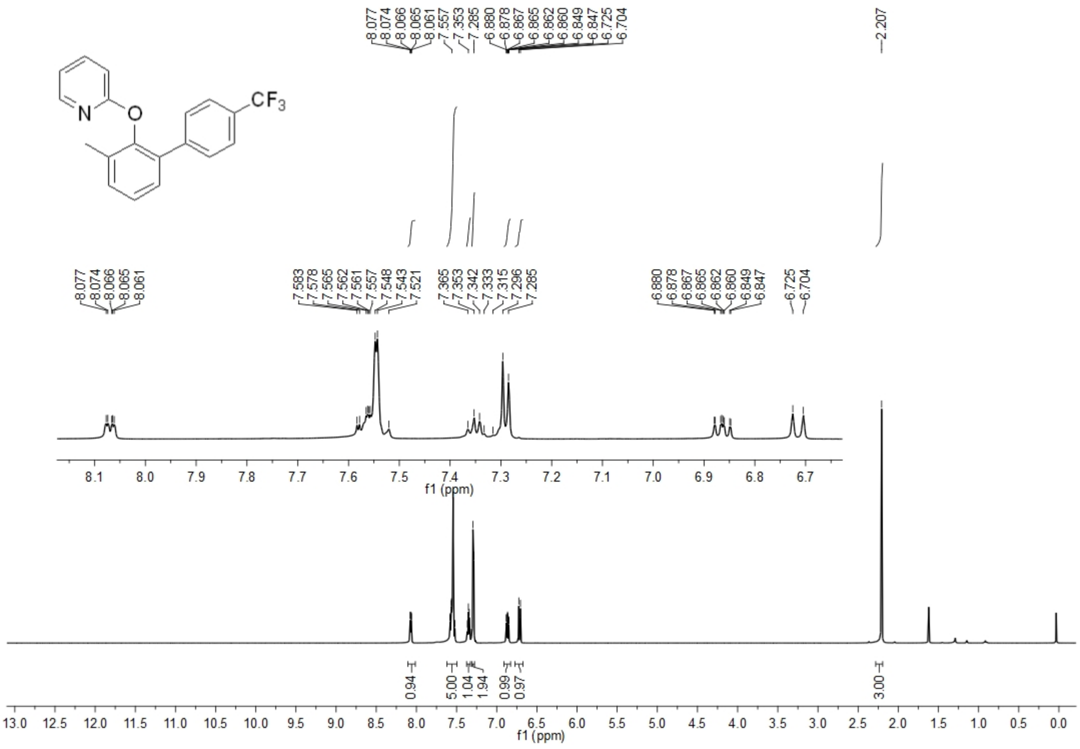

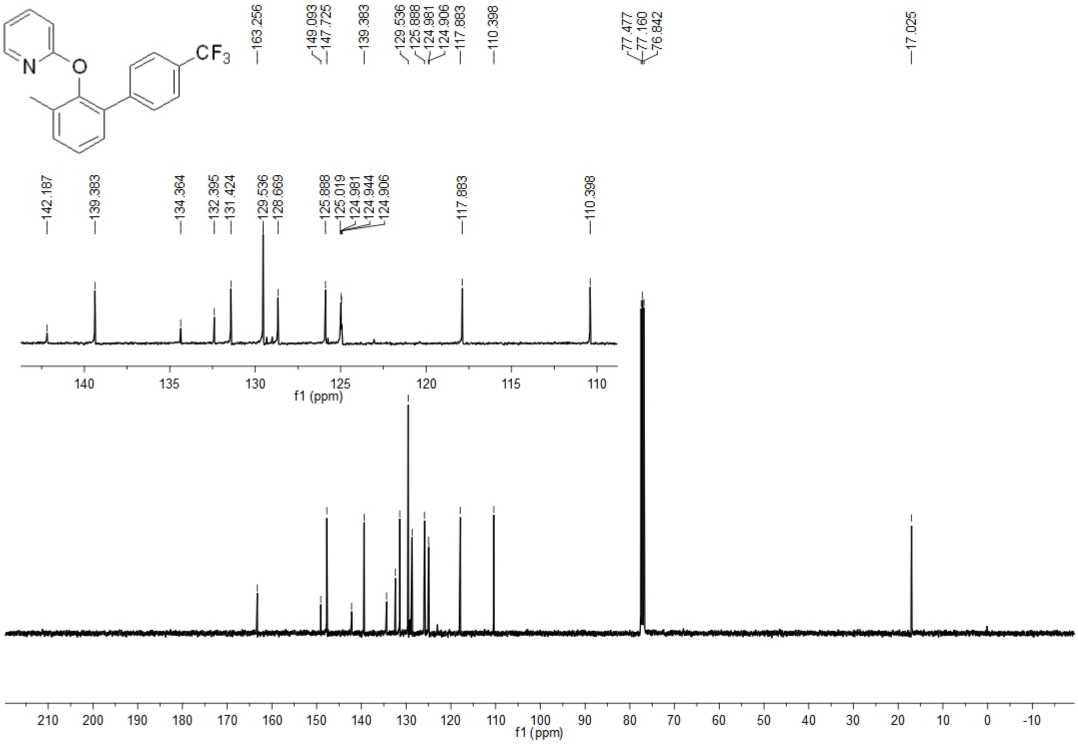

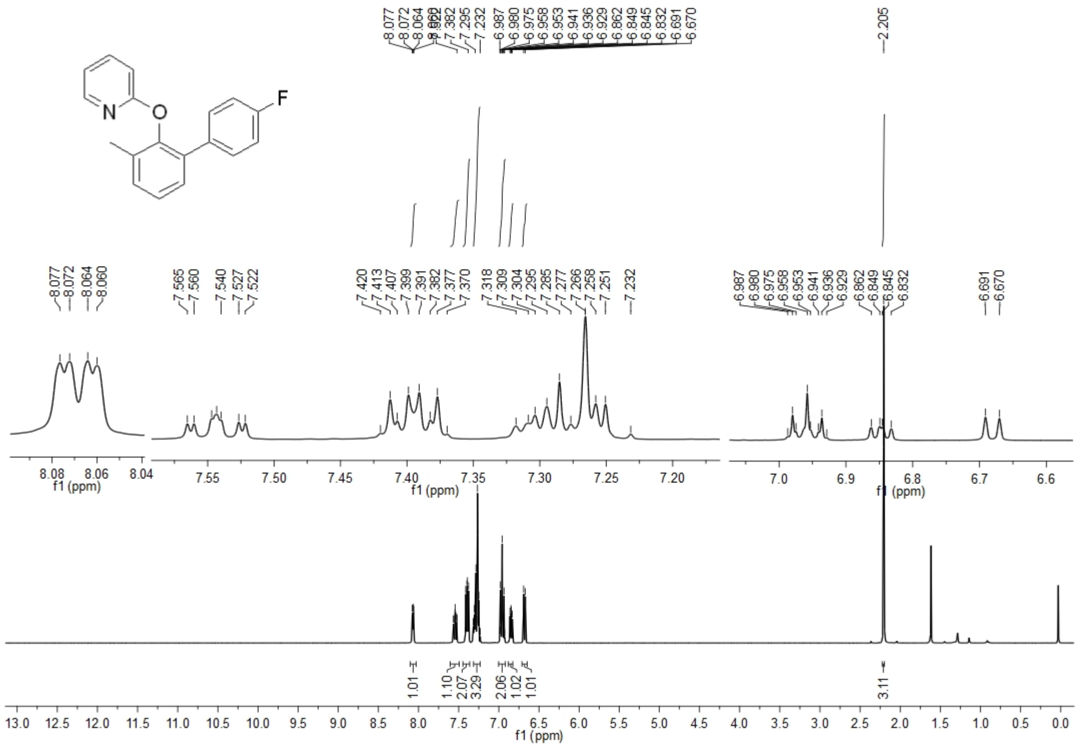

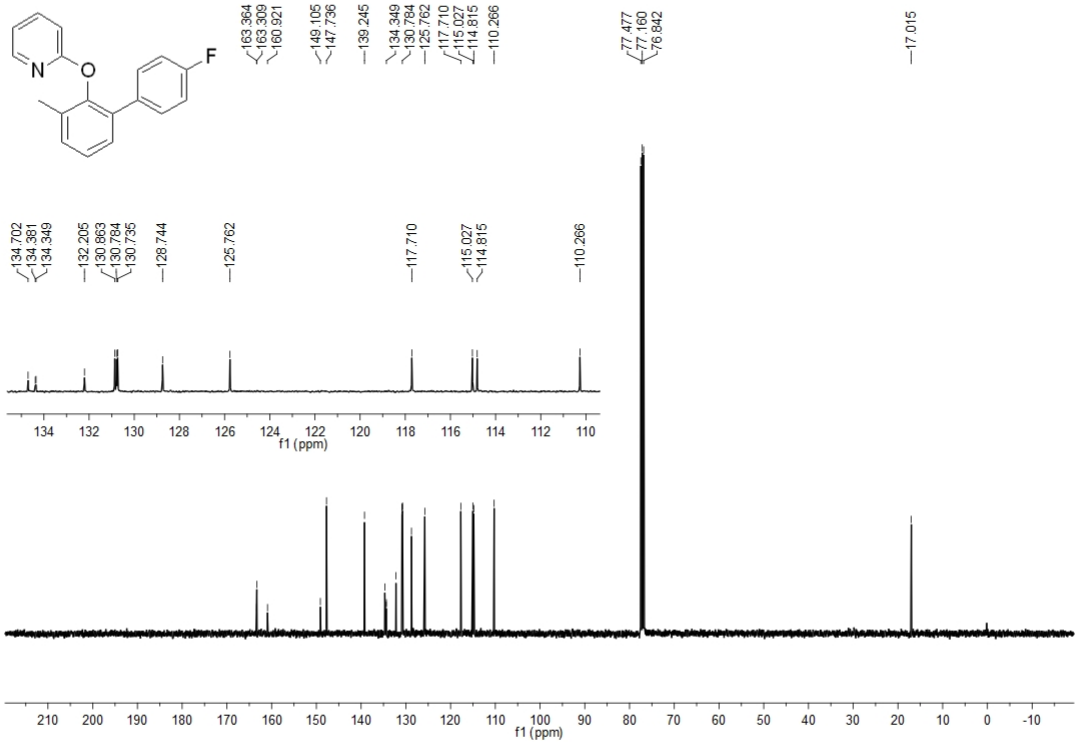

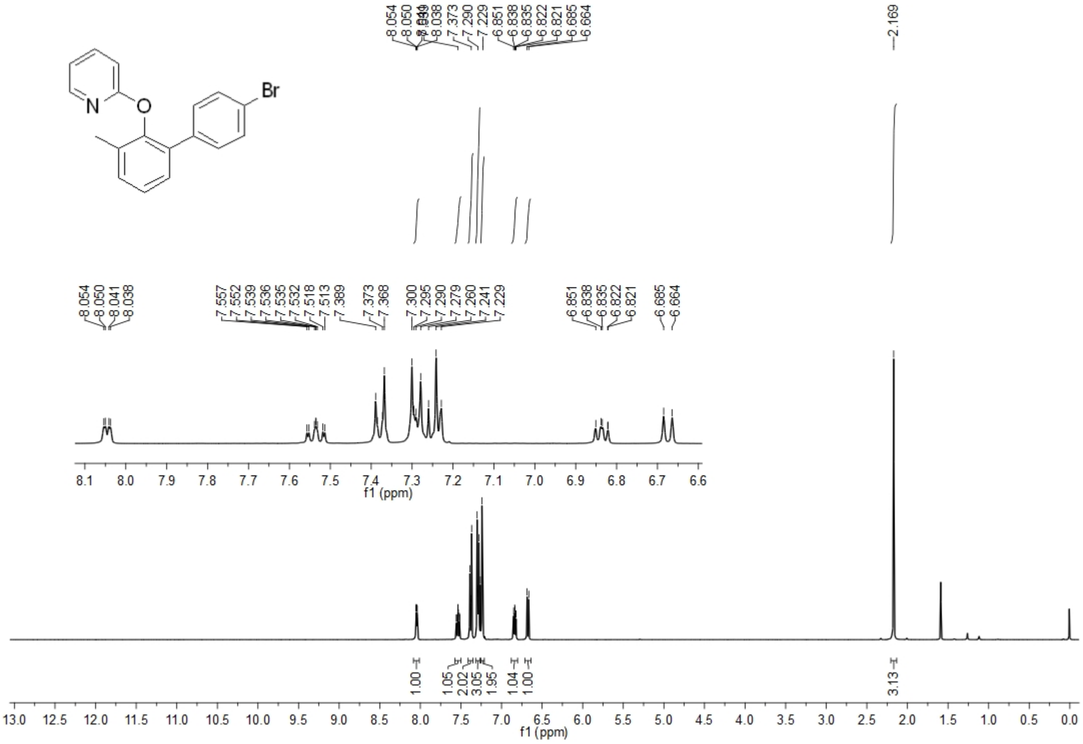
Br
N
O
8.054
8.050
8.038
7.373
7.290
7.229
6.851
6.838
6.835
6.822
6.821
6.685
6.664
2.169
8.054
8.050
8.041
8.038
7.557
7.552
7.539
7.536
7.535
7.532
7.518
7.513
7.389
7.373
7.368
7.300
7.295
7.290
7.279
7.260
7.241
7.229
6.851
6.838
6.835
6.822
6.821
6.685
6.664
f1 (ppm)
1.00
1.05
2.02
3.05
1.95
1.04
1.00
3.13
f1 (ppm)

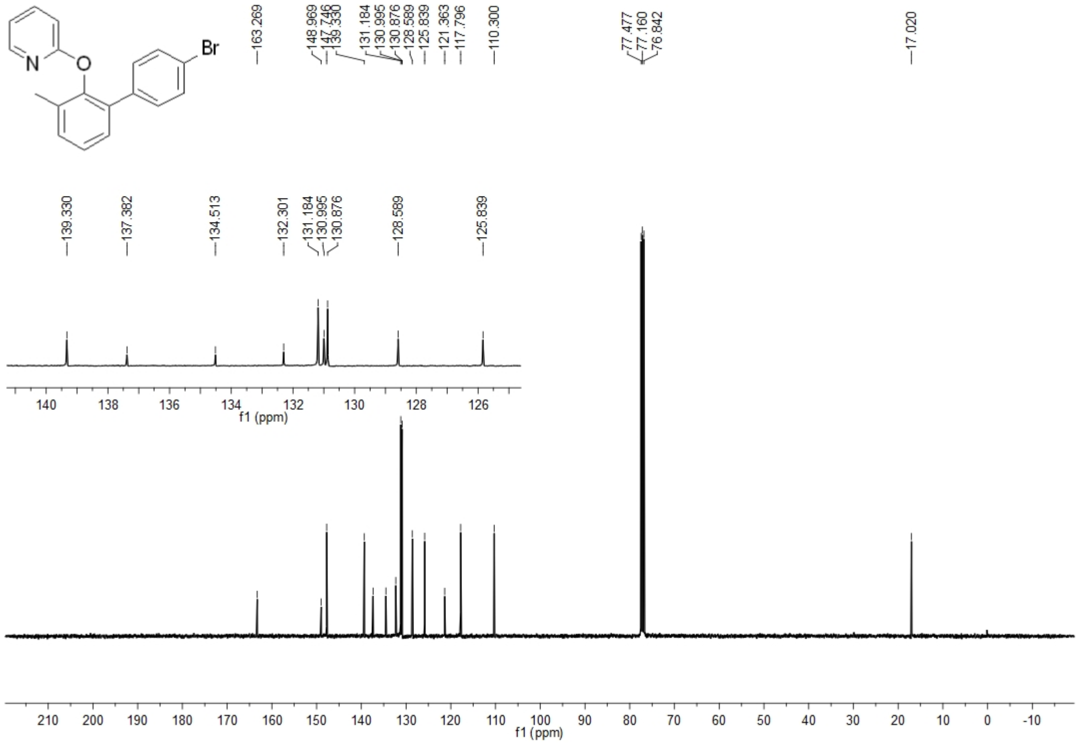
Br
N
O
163.269
148.969
147.746
139.330
131.184
130.995
130.876
128.589
125.839
121.363
117.796
110.300
77.477
77.160
76.842
17.020
139.330
137.382
134.513
132.301
131.184
130.995
130.876
128.589
125.839
f1 (ppm)
f1 (ppm)

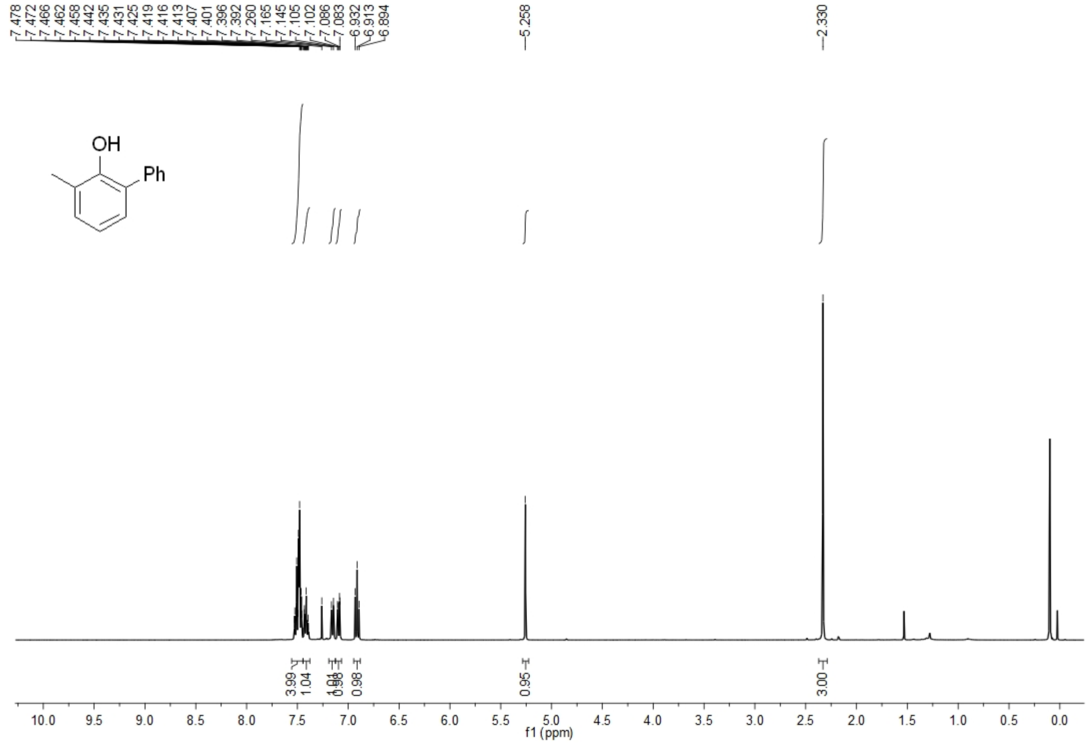

7.478
7.472
7.466
7.462
7.458
7.442
7.435
7.431
7.425
7.419
7.416
7.413
7.407
7.401
7.396
7.392
7.260
7.165
7.145
7.105
7.102
7.086
7.083
6.932
6.913
6.894
5.258
2.330
OH
Ph
3.99
1.04
1.01
0.98
0.98
0.95
3.00
10.0 9.5 9.0 8.5 8.0 7.5 7.0 6.5 6.0 5.5 5.0 4.5 4.0 3.5 3.0 2.5 2.0 1.5 1.0 0.5 0.0
f1 (ppm)
